# Recent Development of Novel Aminoethyl-Substituted Chalcones as Potential Drug Candidates for the Treatment of Alzheimer’s Disease

**DOI:** 10.3390/molecules28186579

**Published:** 2023-09-12

**Authors:** Pratibha Sharma, Manjinder Singh, Varinder Singh, Thakur Gurjeet Singh, Tanveer Singh, Sheikh F. Ahmad

**Affiliations:** 1 Chitkara College of Pharmacy, Chitkara University, Rajpura 140401, Punjab, Indiagurjeet.singh@chitkara.edu.in (T.G.S.); 2 Department of Pharmaceutical Sciences and Technology, Maharaja Ranjit Singh Punjab Technical University, Bathinda 151001, Punjab, India; 3 Department of Neuroscience and Experimental Therapeutics, College of Medicine, Texas A & M Health Science Center, College Station, TX 77807, USA; tanveersingh1988@gmail.com; 4 Department of Pharmacology and Toxicology, College of Pharmacy, King Saud University, Riyadh 11451, Saudi Arabia; fashaikh@ksu.edu.sa

**Keywords:** chalcone, Alzheimer’s disease, AChE, antioxidant, dementia, AGEs

## Abstract

No drug on the market, as a single entity, participates in different pathways involved in the pathology of Alzheimer’s disease. The current study is aimed at the exploration of multifunctional chalcone derivatives which can act on multiple targets involved in Alzheimer’s disease. A series of novel aminoethyl-substituted chalcones have been developed using in silico approaches (scaffold morphing, molecular docking, and ADME) and reported synthetic methods. The synthesized analogs were characterized and evaluated biologically using different in vitro assays against AChE, AGEs, and radical formation. Among all compounds, compound **PS-10** was found to have potent AChE inhibitory activity (IC_50_ = 15.3 nM), even more than the standard drug (IC_50_ = 15.68 nM). Further, the in vivo evaluation of **PS-10** against STZ-induced dementia in rats showed memory improvement (Morris Water Maze test) in rats. Also, **PS-10** inhibited STZ-induced brain AChE activity and oxidative stress, further strengthening the observed in vitro effects. Further, the molecular dynamic simulation studies displayed the stability of the **PS-10** and AChE complex. The novel aminoethyl-substituted chalcones might be considered potential multifunctional anti-Alzheimer’s molecules.

## 1. Introduction

Chalcone, a naturally occurring compound, is the precursor of flavonoids and isoflavonoids and an important constituent of various natural products [1,2]. Chalcone-based derivatives exhibit diverse biological effects, including anti-inflammatory [3,4,5] anticancer [6,7,8,9,10,11], antimalarial [12,13], antidiabetic [14,15,16], antioxidant [17,18], and antimicrobial [19,20]. They are also known for their actions on CNS such as acetylcholinesterase (AChE) and butyrylcholinestrase (BuChE), lipid peroxidation reduction, GABA modulation, and monoamine oxidase inhibition [21,22,23,24,25]. Due to this multi-functional potential, chalcone-based compounds have the ability to manage various complex brain diseases, including Alzheimer’s disease (AD).

AD involves the cascade of anomalies including Aβ aggregates, tau protein hyper-phosphorylation associated with cellular microtubules, and the generation of neurofibrillary tangles (NFTs). Consequently, these anomalies are responsible for several physiological problems such as synaptic damage and increased radical generation and inflammation. Apart from this, the central cholinergic neurons are also severely affected, leading to decreased acetylcholine levels. As a result, a number of physiological activities are affected, such as cognition, thinking ability, language, dependency, and memory functions [26,27]. Moreover, there is evidence proving the effect of oxidative impairment on the endorsement of amyloid aggregates and NFT formation in AD [28]. The large amounts of Fe^2+^ and Cu^2+^ in the brain hasten ROS formation, which further causes Aβ neurotoxicity. Also, the high levels of receptive carbonyls and oxygen radicals lead to AGEs, which cross-link and cause glycation of the tau and Aβ or proteins, ultimately inducing neuron cell death [29].

Although a number of potential targets leading to AD have been identified, only AChE inhibitors (AChEIs) could pave their way into clinics for managing AD. However, these drugs are effective symptomatically, but unable to avert the disease movement [30,31]. Aducanumab, a monoclonal-antibody-based medication that targets the oligomers as well as insoluble fibrils of Aβ plaques, was recently approved by the FDA. It is indicated for patients with mild cognitive impairment [32]. Despite the known multi-etiologies associated with AD, it has been observed that the majority of research is focused on developing molecules that could target one aspect of AD [33,34,35,36]. Such drugs that are single-target-oriented only enable a palliative treatment rather than curing or preventing neurodegenerative multifactorial AD. This could be one of the reasons for the limited success of AChEIs in clinical practice.

**Figure 1:**
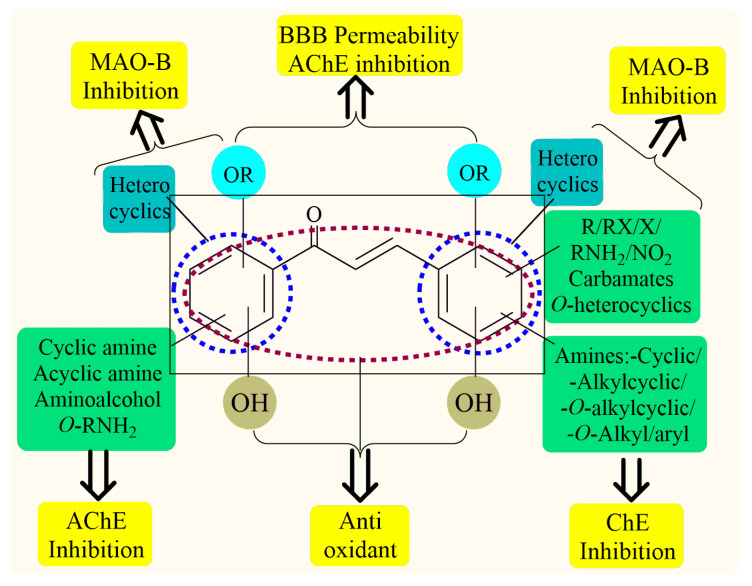
Chalcone-based multifunctional molecules with varied substitutions.

Thus, the development of molecules that have the ability to modulate different key targets of AD simultaneously seems to be the appropriate tool to tackle AD. Further, the compounds having multifunctional potential may have a higher value in terms of their potency due to synergism or limited side effects. This encouraged us to design and develop molecules that could act on different key areas of AD. The research on the development of chalcone-based multifunctional molecules having varied substituents in different positions has been accelerated in recent years (Figure 1) [21,22,23,24,25,26,37,38,39].

Various substitutions on the chalcone skeleton reflect the anti-Alzheimer’s potential in terms of AChE inhibition, MAO-B inhibition, amyloid aggregation inhibition, and antioxidant properties [20,22]. Therefore, chalcone-based novel analogs could be used and taken as a lead for the management of AD. From comprehensive literature analysis, it has been found that no chalcone-based multi-functional molecule is available in clinical practice to manage AD.

The current study involves the design, synthesis, and biological evaluation of chalcone or 1,3-diaryl-2-propen-1-one substituted with varied tertiary amines at ring-B via two carbon spacers. A novel series of aminoethyl-*O*-chalcone by-products were developed using Claisen Schmidt condensation [40,41] and employed for AChE, AGEs, and free radical inhibitory potential in vitro. The most potent developed molecule was further tested in vivo using an STZ-induced rat model of dementia. Also, brain biochemical estimations were performed to delineate the mechanism of action. 

## 2. Results and Discussion

### 2.1. Scaffold-Morphing-Based Bioisosteric Replacement

**Figure 2:**
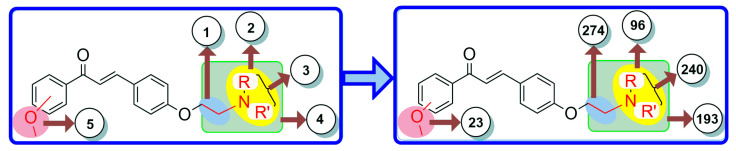
Five bioisosteric sites and the corresponding bioisosteres.

The bioisosteric replacement of chalcone was performed by manual design as well as with a web server (MolOpt) to generate corresponding analogs with their pharmacokinetics and pharmacodynamics. The manual design was based on certain substitutions like the presence of alkoxy groups essential for BBB permeation, and the incorporation of tertiary amines and chain elongation is beneficial for inhibiting AChE. As a result, five potential bioisosteric replacement sites were produced. A total of 826 molecules were generated by the bioisosteric replacement at these five sites (Figure 2). On the basis of synthetic accessibility, the top 40 analogs were used in the pharmacokinetic studies.

### 2.2. ADME Studies

The ADME studies of the top 40 molecules were carried out using SwissADME software (http://www.swissadme.ch/, accessed on 3 January 2021). The leading novel chalcone molecules were identified on the basis of their drug-like properties. All the screened molecules were drug-like molecules as per Lipinski’s rule of five (HB donor ≤ 5, HB acceptor ≤ 10, logPo/w < 5, MW < 500). These drug-like properties are critical for BBB (blood–brain barrier) permeability and, subsequently, the activity of CNS. These molecules displayed a crucial ADME profile with rationalized physicochemical and pharmacokinetic properties (Table 1). The parameters including TPSA, Log P consensus, and Log S ESOL indicated the polarity, lipophilicity, and solubility of the analogs. Further, all molecules exhibited high gastrointestinal absorption, ensuring their significant bioavailability. The anticipated ADME (Absorption, Distribution, Metabolism, and Excretion) profile indicated that all of the highest-ranking molecules possessed drug-like characteristics.

### 2.3. Molecular Docking Studies

AChE is composed of a long channel split into two parts: the peripheral anionic site (PAS) and the catalytic anionic site (CAS). Within the PAS, the amino acids Trp279, Arg289, and Phe330 play critical roles. Meanwhile, the CAS features the catalytic triad consisting of Ser200, Glu327, and His440, along with other significant amino acids like Trp84, Gly119, and Tyr121 [42]. Docking analysis for the screened chalcone molecules was carried out to investigate their interaction with the active gorge of the AChE. The **PS1** was also subjected to docking analysis in order to analyze the interactions of the common moiety with the AChE protein. All the molecules had good binding energies and crucial interactions with the amino acids at both sites (CAS and PAS) of the AChE enzyme. Interestingly, all the analogs have good docking scores as compared to the standard drug (−51.89 Kcal/mol). All of the molecules showed interactions with the His440 and Ser200 residues of the catalytic triad via Pi-alkyl interactions, C-H bond formation, or Van der Waals interactions, including key interactions with Trp84, Gly119, and Tyr121. These molecules also interacted via Phe330, Tyr130, Trp279, and Arg289 of PAS. However, **PS1** lags in terms of binding affinity (−31.90 Kcal/mol) toward the binding site, possibly due to the absence of amine groups and thus relatively smaller size. The binding energies of **PS4**, **PS5**, and **PS** (**8**–**10**) (−52.28 to −63.75 Kcal/mol) were better than the donepezil. Although **PS3**, **PS6**, and **PS7** showed similar binding energy levels (−51.01 to −51.43 Kcal/mol) as donepezil, they interacted with more of the key amino acids. The designed analogs showed significant interactions at both active subsites of AChE, namely PAS (Phe330, Tyr130, Trp279, and Arg289) and CAS (Trp84, Gly119, Tyr121). The docking analysis suggests that the presence of varied amines alters the compounds’ binding affinity with enzymes. The dimethyl-substituted analog (**PS2**) exerted the least binding energy (−49.39 Kcal/mol) compared to the other amine-substituted analogs. The leading analogs with isosteric replacements were identified (Table 2) and were subjected to synthesis and biological evaluations.

### 2.4. Binding Mode and Interactions of ***PS5*** and ***PS10*** with AChE

**Figure 3:**
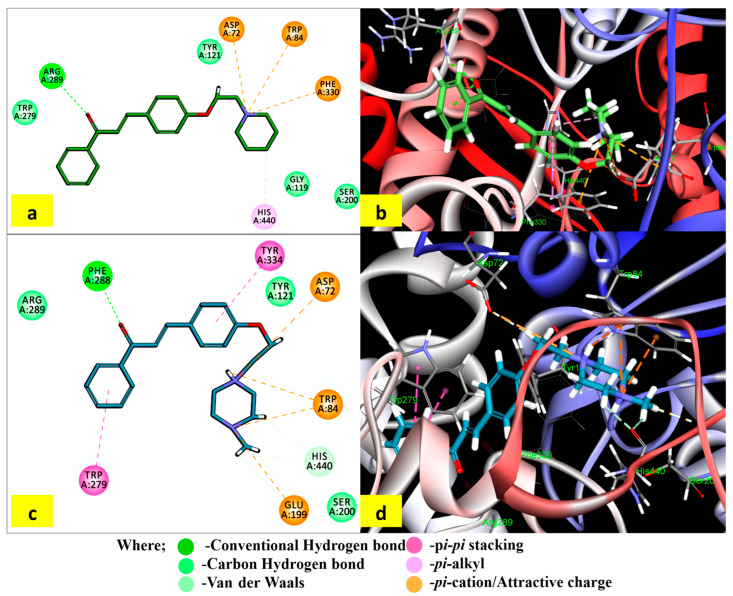
Interactions of compounds **PS5** and **PS10** within the binding site of AChE (1EVE): (**a**,**c**) 2D profile of different interactions of **PS5** and **PS10** with TcAChE, and (**b**,**d**) 3D profile of binding of **PS5** and **PS10** with TcAChE.

The interactions of the most potent in vitro AChE inhibitors (**PS5** and **PS10**) with the crucial residues are displayed in Figure 3**.** The binding of compound **PS5** extended from CAS to PAS in the active site gorge. The carbonylic oxygen formed an H-bond with Arg 289. The quaternary nitrogen of piperidine moiety displayed π–cationic interactions with Trp84, Phe330, and Asp72. The π–alkyl interaction was formed between the carbon atom of piperidine and His440. It additionally created a single hydrogen bond with Arg289 via carbonyl oxygen.

The compound **PS10** underwent pi–pi stacking interactions of both phenyl rings with Trp279 in the acyl binding pocket and Phe334 residue in the PAS, respectively. It formed an H-bond with Phe288 via carbonyl oxygen. Both quaternary nitrogen atoms of 4-methylipiperazine moiety displayed π–cationic interactions with Trp84, Asp72, and Glu199. The docking results revealed the binding capacity of selected molecules with the CAS and PAS residues through the formation of H-bonds; π–π (aromatic), π–cationic, and hydrophobic interactions; and the attractive charges with the AChE active gorge.

### 2.5. Chemistry

**Scheme 1:**
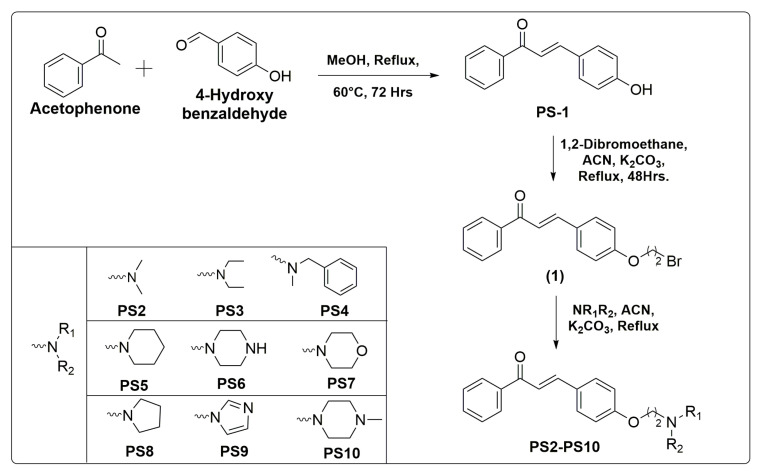
Synthesis of aminoethyl-*O*-chalcone derivatives **PS** (**1**–**10**) with reaction conditions and reagents.

The synthetic methodologies employed to synthesize intermediate (**1**) and final compounds **PS**(**1**–**10**) are given in Scheme 1. The compound **PS1** was synthesized according to the well-established Claisen Schmidt condensation reaction with slight modifications. Further, the prime intermediate (**1**) was synthesized by alkylation of **PS1** with 1,2-dibromoethane in acetonitrile. The incorporation of various secondary amines into intermediate (**1**) in the presence of K_2_CO_3_ in acetonitrile afforded target compounds **PS**(**2**–**10**). The reaction was monitored with TLC plates.

### 2.6. Biological Activity

#### 2.6.1. In Vitro AChE Inhibition Studies 

The ability of target compounds **PS**(**1**–**10**) to inhibit AChE was evaluated by the slightly modified Ellman’s method [43]. The objective of attaching different amines to the 1,3-diphenylprop-2-enone skeleton via an ethoxy spacer was to enhance the interaction of the designed synthesized chalcones with both the PAS and CAS of AChE. Interestingly, most of the tested compounds **PS**(**1**–**10**) had significant AChE inhibitory activity (Table 3). The compounds **PS10** and **PS5**, which had 4-methylpiperazine and piperidine amines attached to chalcone moiety, were found to be the most effective AChE inhibitors with IC_50_ values of 15.3 and 19.71 nM, respectively.

The resulting data showed that the terminal amino group significantly influenced the AChE inhibition. The six-membered cyclic amine-substituted derivatives **PS5**, **PS6**, **PS7**, and **PS10** (IC_50_ = 15.3–22.72 nM) showed better AChE activity than the five-membered cyclic amines **PS8** and **PS9** (IC_50_ = 32.72; 26.3 nM). Further, the aliphatic amine-substituted compounds **PS2**, **PS3**, and **PS4** showed moderate inhibition with IC_50_ values of 52.51, 47.5, and 48.8 nM. This may be due to improper fitting of the five-membered ring systems as well as the aliphatic amines inside the enzyme.

Particularly, the 4-methylpiperazine derivative (**PS10**) was the most potent AChE inhibitor, with IC_50_ = 15.3 nM, comparable to donepezil (IC_50_ = 15.68 nM) and more potent than the unsubstituted piperazine derivative (**PS6**, IC_50_ = 21.4 nM). The aliphatic amine-based derivatives were less effective across the series.

#### 2.6.2. AGE Formation Inhibitory Activity

The synthesized compounds markedly inhibited AGE formations in vitro (Table 3). The compound **PS1** (IC_50_ = 43.6 µM) was the most active among all and showed comparable inhibition to the positive control (IC_50_ = 44.3 µM), while the compounds **PS**(**2**–**4**), **PS6**, and **PS**(**8**–**9**) (IC_50_ = 64.51–69.39 µM) were inferior inhibitors compared to the positive control. The compound **PS10** with 4-methylpiperazine had better anti-glycating effects compared to the unsubstituted piperazine compound (**PS6**). The six-membered cyclic amines (**PS5, PS6**, **PS7**, and **PS10**) were more active than five-membered (**PS8** and **PS9**). The compounds with dimethyl amine (**PS2**), diethyl amine (**PS3**), and *N*-benzylmethylamine moiety (**PS4**) exhibited lower activity ranging from IC_50_ = 67.3 to 69.39 µM.

#### 2.6.3. In Vitro Antioxidant Activity

The antioxidant activity showed that all synthesized compounds exhibited significant radical scavenging activity owing to the presence of an α,β-unsaturated carbonyl system that extended the resonance effect from one ring to another (Table 3). **PS10** was reported as the most potent (EC_50_ = 22.83 nM) and comparable to the standard (EC_50_ = 21.7 nM). Further, **PS1** also exerted comparable free radical scavenging activity (EC_50_ = 22.41 nM) due to the added antioxidant property of the hydroxy group. The compounds **PS5** and **PS7** also showed good radical scavenging activity with EC_50_ values of 34.38 and 37.65 nM, respectively. The free –NH groups enhanced the free radical scavenging along with an α,β-unsaturated carbonyl system due to its electron-rich environment. The rest of the compounds exhibited moderate radical scavenging activity (EC_50_ = 46.7–49.85 nM).

#### 2.6.4. In Vivo Activity

The STZ model (i.c.v., 3 mg/kg) is commonly used to study AD in animals [44,45,46]. The intracerebroventricular injection of STZ, at two sub-diabetogenic doses, induces a range of symptoms including impaired cognition, metabolic and molecular changes, and neuropathological symptoms in rodents which are often found in AD subjects. STZ has been found to disrupt cellular energy metabolism and induce mitochondrial changes, resulting in the generation of ROS [47]. The ROS produced can cause damage to the myelin sheath and acetyl coenzyme-A in the hippocampus. As a result, there is an increase in AChE activity, leading to behavioral disturbances, as in Alzheimer’s disease [48,49,50,51]. Thus, this model is frequently used for AD interventions.

MWM is the most commonly used method to measure learning and spatial memory in rats [52,53]. MWM utilizes the natural swimming ability of rats [53,54]. As a result, during the retrieval phase, there is no induced stress that could affect the results. The MWM model is commonly used to test drugs that enhance memory and provide neuroprotection. Thus, it has been used to understand the effect of **PS10** on the memory of animals with induced cognitive impairment by means of restoring the ELT.

**Figure 4:**
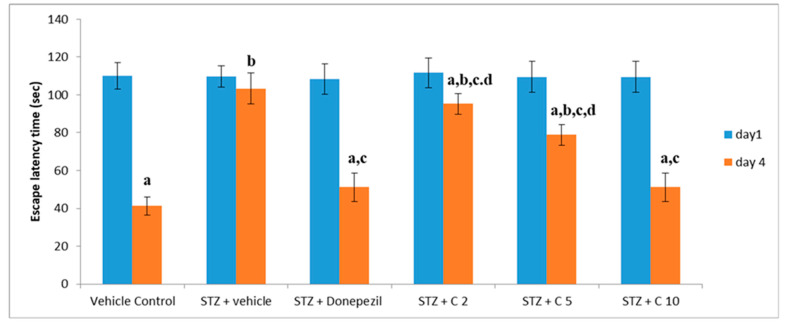
Evaluation of different interventions on ELT at days 1 and 4 employing MWM for memory evaluation. ^a^ *p* < 0.05 vs. day 1 escape latency of vehicle control group; ^b^ *p* < 0.05 vs. day 4 escape latency of vehicle control group; ^c^ *p* < 0.05 vs. day 4 escape latency of STZ + vehicle group; ^d^ *p* < 0.05 vs. day 4 escape latency of STZ + donepezil group.

**Figure 5:**
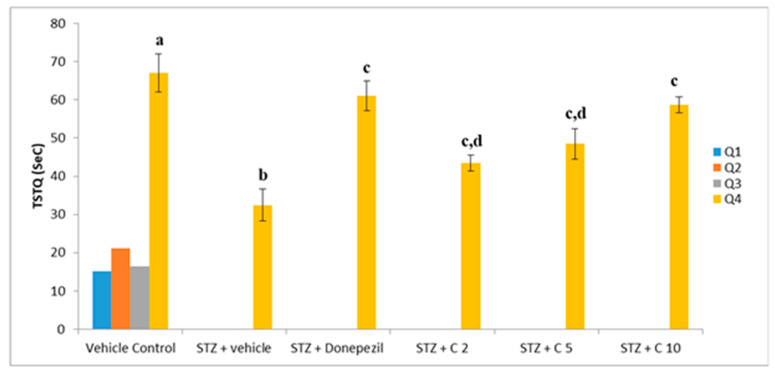
Effect of **PS10** for TSTQ by rat in the MWM. ^a^ *p* < 0.05 vs. q1, q2 and q3 of vehicle control group; ^b^ *p* < 0.05 vs. q4 of vehicle control group; ^c^ *p* < 0.05 vs. q4 of STZ + vehicle group; ^d^ *p* < 0.05 vs. q4 of STZ + donepezil group.

Previous studies have shown that STZ (i.c.v) administration impairs cognitive function in rodents [55]. Consistent with these findings, our study also found declined memory and learning ability in the STZ group than the control animals, which was significantly reversed by treatment with **PS10**. This beneficial effect of **PS10** may be attributed to the structural aspects of chalcone-based compounds, which have been reported as memory enhancers. The administration of **PS10** significantly decreased STZ-induced cognitive impairment by means of restoring the ELT (Figure 4) and TSTQ (Figure 5).

**Figure 6:**
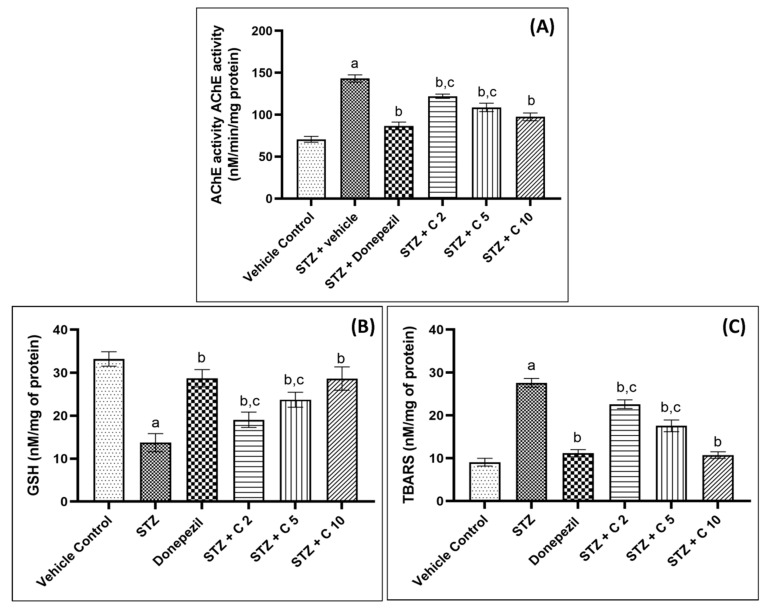
**PS10′**s effect on (**A**) brain AChE activity, (**B**) brain GSH level, and (**C**) brain TBARS levels of rats. ^a^ *p* < 0.05 vs. vehicle control; ^b^ *p* < 0.05 vs. STZ; ^c^ *p* < 0.05 vs. donepezil.

The central cholinergic system is critical for cognitive function, and AD patients often suffer from cholinergic hypofunction due to increased AChE activity and the degeneration of cholinergic neurons [55]. Animal studies have shown that the administration of STZ (i.c.v) increases brain AChE activity, which leads to cognitive impairment and memory deficits [56]. Similarly, in the current study, STZ-administered rats showed higher cerebral AChE activity, which was restored in **PS10**-treated groups (Figure 6). Flavonoids have been shown to inhibit AChE activity by binding strongly to the enzyme’s active sites through interactions with crucial amino acids of AChE, given in Table 2 [57,58,59,60]. Possibly, due to these interactions between **PS10** and amino acids of AChE, **PS10** was able to inhibit AChE activity.

Another crucial factor that contributes to the progression of AD is oxidative stress [61,62]. The brain is more vulnerable to oxidative damage compared to other organs due to its high energy demand, relatively lower level of antioxidant defense mechanisms, and high concentration of polyunsaturated fatty acids [62]. STZ injection generates free radicals that overwhelm the brain’s defensive antioxidant system (GSH) and cause lipid membrane oxidation, leading to the production of malondialdehyde [61,63,64]. Thus, in order to analyze the antioxidant property of **PS10**, the levels of TBARS and GSH were measured in the current investigation (Figure 6). STZ injection was found to increase TBARS and decrease GSH levels in the brain, but these effects were reversed by **PS10** treatment. Previously, antioxidant flavonoids have been reported to exhibit cognitive improvement and neuroprotective effects in a variety of neurological disorders [65,66]. Our study suggests that **PS10** possesses antioxidant properties that reduce lipid peroxidation and spare the antioxidant enzyme GSH, contributing to the memory improvement effects in STZ-induced animals.

### 2.7. Molecular Dynamic Simulation Analysis

**Figure 7:**
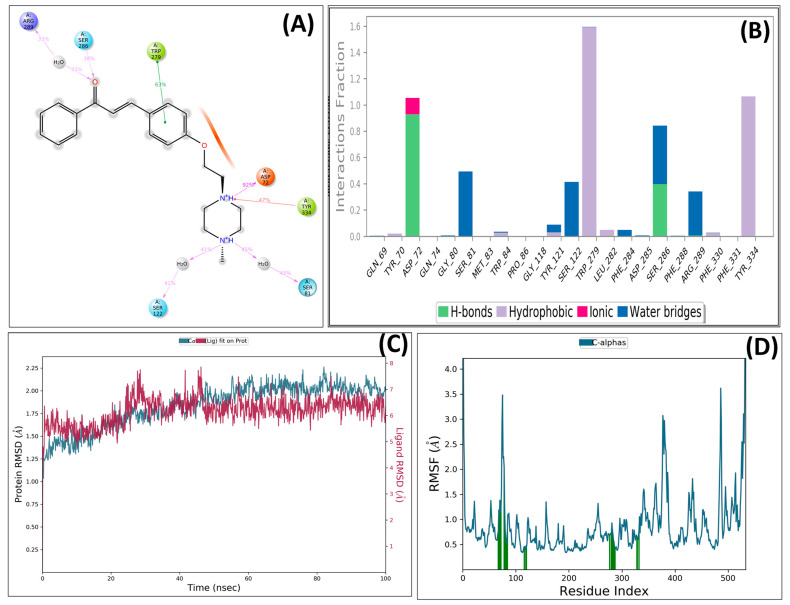
Detailed interactions of **PS10** with AChE enzyme: (**A**) post-MD interactions (H-bond, hydrophobic, and ionic) of **PS-10** with AChE; (**B**) AChE–**PS10** contacts summary during 100 ns MD simulation; (**C**) RMSD plot; (**D**) RMSF plot interacting protein residues.

The stability of the ligand–protein complex was analyzed using a simulation-based interaction protocol. The MD simulation involved the interactions between ligand and protein in dynamic motion and assessed the induced conformational changes at the AChE pocket by ligand binding. The **PS-10** and AChE complex was employed for MD studies. The important interactions were retained throughout the MD simulations with slightly different patterns (Figure 7). Compound **PS-10** interacted with the key residues (Asp72, Arg289, Trp279, and Tyr334) in the active site via ionic and hydrophobic interactions. It formed an H-bond with Asp72 via carbonyl oxygen.

After the MD simulation, the trajectory of the compound was determined by means of RMSD. The RMSD plot indicated that the docked complex remained stable, with minor fluctuations within 1 °A throughout the simulation-induced cognitive impairment by means of restoring **PS10** against AChE. Further, root mean square fluctuation (RMSF) plots for protein were also obtained (Figure 7). The vertical green bars represent the interacting protein residues with ligands during simulation.

## 3. Material and Methods

### 3.1. Scaffold Morphing

Scaffold morphing, a technique for drug designing, has been used for the design of novel chalcone molecules. It provides rationalized structural modifications within the parent molecule with improved physicochemical properties [67,68]. This study was carried out using the web server MolOpt (https://xundrug.cn/molopt accessed on 5 January 2021) recently developed web tool for scaffold morphing for bioisosteric transformation [69]. The transformation involves the replacement of the functional groups or other parts of the molecule with their bioisosteres on key molecules. By manually designing and ‘data mining’ an inbuilt module of MolOpt, the five replaceable sites of the designed chalcone compound were generated. The molecules that were produced were evaluated to determine if they could be feasibly synthesized. The feasibility of synthesis was rated from 1 (very easy) to 10 (very difficult), and a threshold of 2.5 was used to screen the molecules. The best molecules were then subjected to pharmacokinetic and molecular docking analyses using computational methods.

### 3.2. Prediction of In Silico Pharmacokinetic Properties

**Figure 8:**
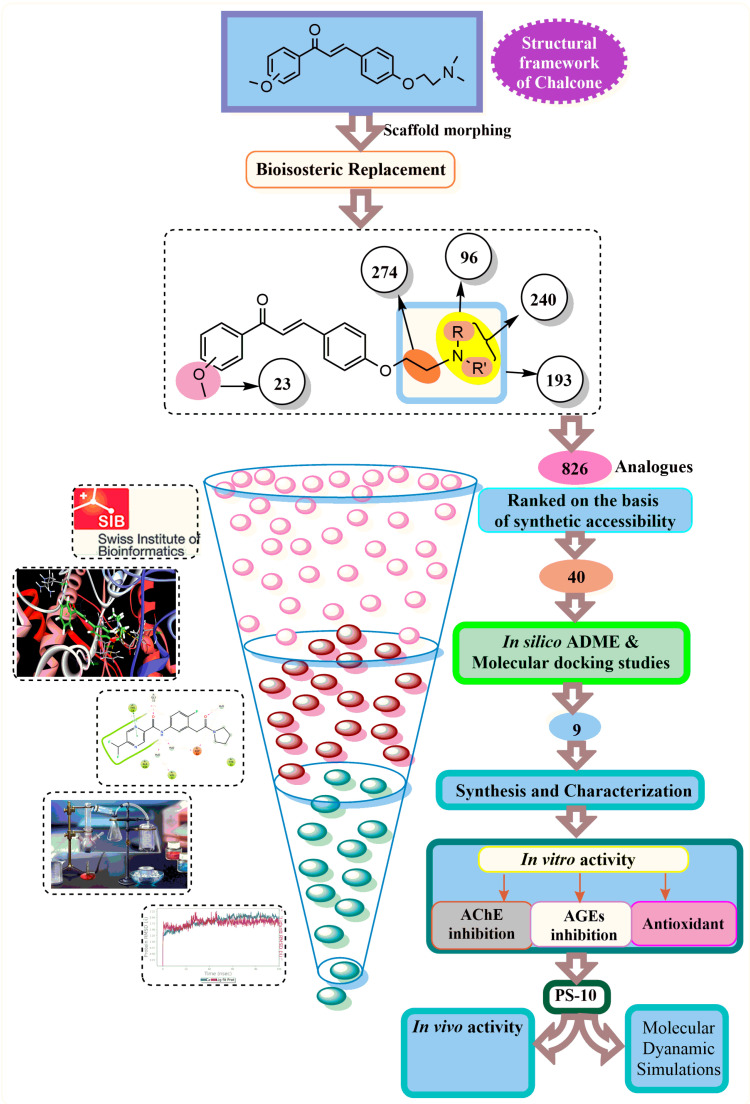
Various steps involved in the current study.

The kinetic profiles of designed chalcone molecules, i.e., ADME, were predicted using SwissADME, a free web application for analyzing the pharmacokinetic profile of a molecule [70]. Various parameters such as physicochemical properties, lipophilicity, and solubility patterns are predicted on the basis of topological polar surface area (TPSA), consensus logP, and ESOL LogS; additionally, Lipinski’s rule of five (molecular weight and H-bond modulator) for the drug-likeness assessment was considered [71]. The gastrointestinal absorption and brain permeability of the target molecules were also assessed [72,73]. The details of the current study are outlined in Figure 8.

### 3.3. Molecular Docking

Biovia Discovery Studio software (2019) was used to carry out the molecular docking experiments. To begin the process, the 3D X-ray crystal structure of acetylcholinesterase (AChE) with a PDB ID of 1EVE (2.50 Å) was obtained from the Protein Data Bank (www.rcsb.org, accessed on 10 January 2021). The structures were then preprocessed, prepared, and optimized using the ‘Macromolecule’ module. The protein preprocessing was carried out by the removal of water molecules and heteroatoms, and protonation using the ‘Add Polar’ option. Next, the preparation and generation of binding sites around the co-crystallized ligands was achieved using the ‘Define and Edit Binding Site’ tool. The SMILES notations and structures of the molecules and donepezil were prepared using MarvinSketch and ChemDraw 16.0 software. Ligands were prepared and minimized using the ‘Full Minimization’ of the ‘Small Molecules’ module. The docking analysis was performed using the ‘Dock Ligands (CDOCKER)’ protocol. The native ligand, donepezil (standard drug), was also re-docked at the active site of the AChE, and we compared the interaction behavior with the designed molecules. The molecular interactions of docked complexes of all the molecules with AChE were analyzed by visual inspection. The preparation and generation of binding sites around the co-crystallized ligands were dependent on the docking scores of AChE amino acids.

### 3.4. Chemistry

All chemicals were taken from marketable suppliers and were used as such. A magnetic stirrer, hot plate (Remi), and rotary evaporator (Perfit) were used for solvent evaporation and recovery. The progression and completion of all the chemical reactions were checked using TLC. Silica columns were employed to purify synthesized compounds followed by melting point determination.

General procedure for the synthesis of (**PS1**): The mole equivalents of acetophenone and *p*-hydroxybenzaldehyde were stirred with reflux at 60 °C for 72 h in methanol under mildly acidic conditions to afford **PS1**. The crude product was treated with water and dried. The compound was recrystallized with alcohol (Scheme 1).

General procedure for the synthesis of **PS-1**: 3-(4-Hydroxyphenyl)-1-phenylprop-2-en-1-one (2 mmol) (**PS-1**) and 1,2-dibromoethane (2 mmol) were stirred in acetonitrile in the presence of anhydrous potassium carbonate (10 mmol). The solvent was evaporated and the crude product was thoroughly washed with water, and dried. Then, intermediate **1** was purified on a silica column using hexane:ethylacetate (8:2) as a solvent system.

General procedures for the synthesis of final compounds **PS-2** to **PS-10**: To a solution of intermediate **1** (0.5 mmol) in acetonitrile, different amines (1.0 mmol) and anhydrous K_2_CO_3_ (2.5 mmol) were added. After stirring for 48 h, the solvent was removed under vacuum and the crude product was washed with water, filtered, and dried, and then purified on silica columns using CHCl_3_:MeOH (9:1) as a solvent system. The various spectra’s can be found in Supplementary Materials.

*3-(4-Hydroxyphenyl)-1-phenylprop-2-en-1-one* *(***PS-1***)*: Solid crystalline, yellow, yield 70%, m.p.: 187–189 °C; ^1^H NMR (500 MHz, CDCl_3_, δ ppm): 8.01–7.99 (2H, m, *ArH*), 7.79–7.76 (1H, d, *J* = 15.65 Hz, -CH), 7.58–7.55 (3H, d, m, *ArH*), 7.51–7.48 (2H, t, *J*_1_ = 7.85, *J*_2_ = 7.25 Hz, *ArH*), 7.42–7.39 (1H, d, *J* = 15.65 Hz, -CH), 6.90–6.88 (2H, m, *ArH*), 5.52 (1H, brs, OH). ^13^C NMR (500 MHz, DMSO, δ ppm): 188.87, 160.07, 144.40, 137.86, 132.6, 130.85, 128.54, 128.18, 125.62, 118.35, 115.71; IR(KBr): 3500–3100 cm^−1^ (-OH str, m); 1647 cm^−1^ (C=O, s); 1591 cm^−1^ (Ar C=C, s). MS (ESI) *m*/*z* = 225.11 (M + H)^+^. R_f_ value: 0.42 (hexane:ethylacetate, 7:3).

*3-(4-(2-Bromoethoxy)phenyl)-1-phenylprop-2-en-1-one* *(***1***)*: Solid crystalline white, yield 70%, m.p.: 86–87 °C; ^1^H NMR (500 MHz, CDCl_3_, δ ppm): 8.01–7.99 (2H, d, *J* = 7.8 Hz, *ArH*), 7.78–7.75 (1H, d, J =12.52 Hz, -CH), 7.62–7.55 (3H, m, *ArH*), 7.51–7.48 (2H, t, *J*_1_ =7.75, *J*_2_ = 7.35 Hz, *ArH*), 7.44–7.40 (1H, d, *J* = 15.65 Hz, -CH), 6.95–6.93 (2H, d, *J* = 8.7 Hz, *ArH*), 4.34–4.31 (2H, t, *J*_1_ = *J*_2_ = 6.25 Hz, -CH_2_), 3.66–3.63 (2H, t, *J*_1_ = *J*_2_ = 6.25 Hz, -CH_2_), ^13^C NMR (500 MHz, CDCl_3_, δ ppm): 190.61, 160.58, 144.62, 138.49, 132.54, 130.26, 128.57, 128.43, 127.97, 120.12, 114.94, 65.02, 29.96; IR(KBr, cm^−1^): 3059 cm^−1^ (=C-H str, m); 1645 cm^−1^ (C=O) (s); 1605 cm^−1^ (Ar C=C str, m); MS (ESI) *m*/*z* = 331.03 (M + H)^+^. R_f_ value: 0.38 (hexane:ethylacetate, 8:2).

*3-(4-(2-(Dimethylamino)ethoxy)phenyl)-1-phenylprop-2-en-1-one* *(***PS-2***)*: Intermediate **1** was treated with dimethylamine according to the general procedure to give the desired product **PS-2** as yellow, solid, yield 60%, m.p.: 92–95 °C; IR(KBr, cm^−1^): 1655 cm^−1^ (C=O, s); 1596 cm^−1^ (Ar C=C str, s), 1300–1100 cm^−1^ (C-N, m); ^1^H NMR (500 MHz, CDCl_3_, δ ppm): 7.90–7.89 (2H, d, *J* = 7.82, 1.35 Hz, *ArH*), 7.68–7.65 (1H, d, *J* = 15.65 Hz, -CH), 7.49–7.43 (3H, m, *ArH*), 7.40–7.36 (2H, m, *ArH*), 7.32–7.29 (1H, d, *J* = 15.65 Hz, -CH), 6.85–6.83 (2H, d, *J* = 8.7 Hz, *ArH*), 4.02–3.98 (2H, m, -CH_2_), 2.64–2.62 (2H, t, *J* = 5.65, 5.7 Hz, -CH_2_), 2.23–2.21 (m, 6H, N(CH_3_)_2_), ^13^C NMR (500 MHz, CDCl_3_, δ ppm): 190.62, 158.65, 144.45, 138.46, 132.27, 130.24, 128.44, 128.33, 127.22, 119.81, 114.73, 66.48, 61.63, 46.12; MS (ESI) *m*/*z* = 296.16 (M + H)^+^. R_f_ value: 0.62 CHCl_3_:MeOH, 9:1).

*3-(4-(2-(Diethylamino)ethoxy)phenyl)-1-phenylprop-2-en-1-one* *(***PS-3***)*: Intermediate **1** was treated with diethylamine according to the general procedure to give the desired product **PS-3** as light yellow, solid, yield 55%, m.p.: 89–91 °C; IR(KBr, cm^−1^): 1655 cm^−1^ (C=O, s); 1590 cm^−1^ (Ar C=C str, s), 1300–1100 cm^−1^ (C-N, m); ^1^H NMR (500 MHz, CDCl_3_, δ ppm): 7.93–7.92 (2H, d, *J* = 7.4 HZ, *ArH*), 7.72–7.68 (1H, d, *J* = 15.7 Hz, -CH), 7.55–7.47 (3H, m, *ArH*), 7.43–7.31 (3H, m, *ArH*, -CH), 6.86–6.85 (2H, d, *J* = 8.55 Hz, *ArH*), 4.04–4.02 (2H, t, *J*_1_ = *J*_2_ = 6.25 Hz, -CH_2_), 2.85–2.83 (2H, t, *J*_1_ = *J*_2_ = 6.1 Hz, -CH_2_), 2.62–2.57 (4H, q, *J*_1_ = *J*_2_ = *J*_3_ = 7.1 Hz, 2(-CH_2_)), 1.02–1.00 (6H, t, *J*_1_ = *J*_2_ = 7.1 Hz, 2(-CH_3_)); ^13^C NMR (500 MHz, CDCl_3_, δ ppm): 190.61, 160.58, 144.12, 138.44, 132.34, 130.24, 128.54, 128.33, 127.21, 119.79, 114.71, 66.11, 61.62, 46.12, 15.06; MS (ESI) *m*/*z* = 324.19 (M + H)^+^. R_f_ value: 0.56 (CHCl_3_:MeOH, 9:1).

*3-(4-(2-(Benzyl(methyl)amino)ethoxy)phenyl)-1-phenylprop-2-en-1-one* *(***PS-4***)*: Intermediate **1** was treated with N-benzylmethylamine according to the general procedure to give the desired product **PS-4** as yellow, solid, yield 55%, m.p.: 115–117 °C; IR(KBr, cm^−1^): 1655 cm^−1^ (C=O, s); 1590 cm^−1^ (Ar C=C str, s), 1300–1100 cm^−1^ (C-N, m); ^1^H NMR (500 MHz, CDCl_3_, δ ppm): 8.01–7.99 (2H, d, *J* = 7.35, 1.35 Hz, *ArH*), 7.79–7.76 (1H, d, *J* = 15.5 Hz, -CH), 7.62–7.55 (3H, m, *ArH*), 7.52–7.45 (2H, m, *ArH*), 7.44–7.39 (1H, d, *J* = 15.45 Hz, -CH), 7.34–7.30 (3H, m, *ArH*), 7.27–7.25 (2H, d, *J* = 8.15 Hz, *ArH*), 6.92–6.90 (2H, d, *J* = 8.15 Hz, *ArH*), 4.14–4.12 (2H, t, *J* = 6.25 Hz, -CH_2_), 3.63 (2H, s, -CH_2_), 2.87–2.84 (2H, t, *J* = 5.75 Hz, -CH_2_), 2.36 (3H, s, -CH_3_); ^13^C NMR (500 MHz, CDCl_3_, δ ppm): 190.55, 160.94, 144.74, 138.54, 138.34, 130.37, 130.24, 129.12, 128.58, 128.44, 128.33, 127.22, 119.81, 117.13, 115.01, 66.48, 62.68, 55.56, 42.95; MS (ESI) *m*/*z* = 372.19 (M + H)^+^; R_f_ value: 0.51 (CHCl_3_:MeOH, 9:1).

*1-Phenyl-3-(4-(2-(piperidin-1-yl)ethoxy)phenyl)prop-2-en-1-one* *(***PS-5***)*: Intermediate **1** was treated with N-benzylmethyl amine according to the general procedure to give the desired product **PS-5** as light brown, solid, yield 63%, m.p.: 89–91 °C; IR(KBr, cm^−1^): 1655 cm^−1^ (C=O, s); 1590 cm^−1^ (Ar C=C str, s), 1300–1100 cm^−1^ (C-N, m); ^1^H NMR (500 MHz, CDCl_3_, δ ppm): 8.02–7.99 (2H, m, *ArH*), 7.79–7.76 (1H, d, *J* = 15.65 Hz, -CH), 7.63–7.55 (3H, m, *ArH*), 7.51–7.48 (2H, m, *ArH*), 7.42–7.39 (1H, d, *J* = 15.65 Hz, -CH), 6.94–6.93 (2H, d, *J* = 8.75, *ArH*), 4.18–4.15 (2H, t, *J* = 6 Hz, -CH_2_), 2.83–2.81 (2H, t, *J* = 5.9 Hz, -CH_2_), 2.55 (4H, s, 2(CH_2_)), 1.65–1.46 (6H, m, 3(CH_2_)); ^13^C NMR (500 MHz, CDCl_3_, δ ppm): 190.61, 160.90, 144.71, 138.54, 132.55, 130.22, 128.57, 128.43, 127.71, 119.85, 115.04, 66.04, 57.72, 55.05, 25.79, 24.06; MS (ESI) *m*/*z* = 336.20 (M + H)^+^; R_f_ value: 0.51 (CHCl_3_:MeOH, 9:1).

*1-Phenyl-3-(4-(2-(piperazin-1-yl)ethoxy)phenyl)prop-2-en-1-one* *(***PS-6***)*: Intermediate **1** was treated with piperazine according to the general procedure to give the desired product **PS-6** as light yellow, solid, yield 65%, m.p: 96–99 °C; IR(KBr, cm^−1^): 1655 cm^−1^ (C=O, s); 1596 cm^−1^ (Ar C=C str, s), 1300–1100 cm^−1^ (C-N, m); ^1^H NMR (500 MHz, CDCl_3_, δ ppm): 8.01–7.99 (2H, m, *ArH*), 7.88–7.86 (1H, d, *J* = 11.95 Hz, -CH), 7.80–7.74 (2H, m, *ArH*), 7.52–7.47 (2H, m, *ArH*), 7.18–7.14 (1H, m, *ArH*), 7.00–6.98 (1H, d, *J* = 8.7 Hz, -CH), 6.94–6.92 (2H, d, *J* = 8.7, *ArH*), 4.16–4.14 (2H, t, *J* = 5.8 Hz, -CH_2_), 2.93–2.91 (4H, t, *J* = 4.7 Hz, 2(CH_2_)), 2.82–2.80 (2H, t, *J* = 5.8 Hz, -CH_2_), 2.77–2.75 (4H, t, *J* = 5.7 Hz, 2(CH_2_)), 2.65 (1H, s, NH); ^13^C NMR (500 MHz, CDCl_3_, δ ppm): 190.60, 160.88, 144.68, 138.51, 132.57, 130.23, 128.54, 128.42, 127.74, 119.87, 115.04, 66.00, 57.68, 54.89, 45.97; MS (ESI) *m*/*z*= 337.19 (M + H)^+^; R_f_ value: 0.25 (CHCl_3_:MeOH, 9:1).

*3-(4-(2-Morpholinoethoxy)phenyl)-1-phenylprop-2-en-1-one* *(***PS-7***)*: Intermediate **1** was treated with morpholine according to the general procedure to give the desired product **PS-7** as light pink, solid, yield 55%, m.p.: 104-106 °C; IR(KBr, cm^−1^): 1655 cm^−1^ (C=O, s); 1591 cm^−1^ (Ar C=C str, s), 1300–1100 cm^−1^ (C-N, m); ^1^H NMR (500 MHz, CDCl_3_, δ ppm): 8.01–8.00 (2H, d, *J* = 6.45 Hz, *ArH*), 7.79–7.76 (1H, d, *J* = 15.65 Hz, -CH), 7.62–7.55 (3H, m, *ArH*), 7.51–7.48 (2H, t, *J* = 7.55, 7.35 Hz, *ArH*), 7.44–7.39 (1H, d, *J* = 15.65 Hz, -CH), 6.94–6.93 (2H, d, *J* = 8.55 Hz, *ArH*), 4.17–4.15 (2H, t, *J* = 5.55 Hz, -CH_2_), 3.75–3.73 (4H, t, *J* = 4.4 Hz, 2(CH_2_)), 2.84–2.82 (2H, t, *J* = 5.55 Hz, -CH_2_), 2.59 (4H, s, 2(CH_2_)); ^13^C NMR (500 MHz, CDCl_3_, δ ppm): 190.58, 160.78, 144.61, 138.51, 132.58, 130.23, 128.58, 128.43, 127.83, 119.93, 115.10, 66.88, 65.95, 57.51, 54.10; MS (ESI) *m*/*z* = 338.20 (M + H)^+^; R_f_ value: 0.36 (CHCl_3_:MeOH, 9:1).

*1-Phenyl-3-(4-(2-(pyrrolidin-1-yl)ethoxy)phenyl)prop-2-en-1-one* *(***PS-8***)*: Intermediate **1** was treated with pyrrolidine according to the general procedure to give the desired product **PS-8** as dark brown, solid crystalline, yield 50%, m.p: 105-107 °C; IR(KBr, cm^–1^): 1655 cm^–1^ (C=O, s); 1591 cm^–1^ (Ar C=C str, s), 1300–1100 cm^–1^ (C-N, m); ^1^H NMR (500 MHz, CDCl_3_, δ ppm): 7.65–7.50 (3H, m, *ArH*, -CH), 7.44–7.40 (2H, t, *J* = 7.8 Hz, *ArH*), 7.37–7.32 (1H, m, *ArH*), 7.21–7.19 (2H, d, *J* = 8.55 Hz, *ArH*), 6.99–6.97 (2H, d, *J* = 8.6 Hz, *ArH*) or 6.99–6.94 (2H, m, *ArH*), 6.71–6.68 (1H, d, *J* = 14.35 Hz, -CH), 4.17–4.14 (2H, t, *J* = 5.9 Hz, CH_2_), 2.95–2.92 (2H, t, *J* = 6 Hz, CH_2_), 2.80–2.56 (4H, m, 2(CH_2_)), 1.95–1.76 (4H, m, 2(CH_2_)); ^13^C NMR (500 MHz, CDCl_3_, δ ppm): 199.49, 158.32, 148.56, 142.73, 135.48, 130.16, 128.76, 127.41, 126.19, 125.14, 114.88, 67.00, 55.04, 54.66, 23.49; MS (ESI) *m*/*z* = 322.18 (M + H)^+^; R_f_ value: 0.51 (CHCl_3_:MeOH, 9:1).

*3-(4-(2-(1H-Imidazol-1-yl)ethoxy)phenyl)-1-phenylprop-2-en-1-one* *(***PS-9***)*: Intermediate **1** was treated with pyrrolidine according to the general procedure to give the desired product **PS-9** as light yellow, solid, yield 70%, m.p.: 93–96 °C; IR(KBr, cm^−1^): 1655 cm^−1^ (C=O, s); 1591 cm^−1^ (Ar C=C str, s), 1300–1100 cm^−1^ (C-N, m); ^1^H NMR (500 MHz, CDCl_3_, δ ppm): 8.02–7.99 (2H,m, *ArH*), 7.80–7.77 (1H, d, *J* = 15.55 Hz, CH), 7.62–7.61 (2H, d, *J* = 8.7 Hz, *ArH*), 7.59–7.56 (1H, t, *J* = 7.5 Hz, *ArH*), 7.51–7.48 (2H, t, *J* = 7.75, 7.3 Hz, *ArH*), 7.44–7.40 (1H, dd, *J* = 15.45, 7.3 Hz, -CH), 7.09–7.05 (1H, d, *ArH*), 7.05–7.03 (1H, m, *ArH*), 7.00–6.98 (2H, d, *J* = 8.7 Hz, *ArH*), 6.90–6.89 (1H, d, *J* = 8.7 Hz, *ArH*), 4.37–4.35 (2H, t, *J* = 4.95, 5.1 Hz, -CH_2_), 4.27–4.25 (2H, t, *J* = 5.1 Hz, -CH_2_); ^13^C NMR (500 MHz, CDCl_3_, δ ppm): 190.58, 160.59, 144.50, 138.48, 138.40, 132.72, 130.27, 128.60, 128.17, 128.07, 120.40, 120.14, 117.13, 115.10, 66.53, 46.45; MS (ESI) *m*/*z* = 319.14 (M + H)^+^; R_f_ value: 0.48 (CHCl_3_:MeOH, 9:1).

*3-(4-(2-(p-Methylpiperazin-1-yl)ethoxy)phenyl)-1-phenylprop-2-en-1-one* *(***PS-10***)*: Intermediate **1** was treated with *N*-methylpiperazine according to the general procedure to give the desired product **PS-10** as white, solid, yield 60%, m.p.: 138-141 °C; IR(KBr, cm^−1^): 1655 cm^−1^ (C=O, s); 1590 cm^−1^ (Ar C=C str, s), 1300–1100 cm^−1^ (C-N, m); ^1^H NMR (500 MHz, CDCl_3_, δ ppm): 8.02–7.99 (2H,m, *ArH*), 7.80–7.76 (1H, dd, *J* = 15.65, 6.15 Hz, -CH), 7.63–7.55 (3H, m, *ArH*), 7.51–7.48 (2H, m, *ArH*), 7.44–7.39 (1H, dd, *J* = 9.6, 6 Hz, -CH), 7.00–6.93 (2H, dd, *J* = 8.7 Hz, *ArH*), 6.90–6.89 (1H, d, *J* = 8.7 Hz, *ArH*), 4.16–4.14 (2H, t, *J* = 5.8 Hz, -CH_2_), 2.85–2.83 (2H, t, *J* = 5.8, 5.01 Hz, -CH_2_), 2.64 (4H, s, -2(CH_2)_), 2.51 (4H, s, -2(CH_2)_), 2.31 (3H, s, CH_3_); ^13^C NMR (500 MHz, CDCl_3_, δ ppm): 190.59, 160.87, 144.66, 138.53, 132.56, 130.26, 128.59, 128.43, 127.76, 119.88, 115.04, 66.15, 57.01, 55.00, 53.50, 45.94; MS (ESI) *m*/*z* = 351.21 (M + H)^+^; R_f_ value: 0.24 (CHCl_3_:MeOH, 9:1).

### 3.5. Biological Activity

#### 3.5.1. In Vitro Studies

##### In Vitro AChE Inhibition

The inhibitory potential of synthesized compounds was evaluated for brain AChE by the widely used Ellman et al. process [43,57,58]. The method involved using spectroscopic investigation at 450 nm. All the compounds with different concentrations were evaluated in triplicate including donepezil (standard AChE inhibitor).

##### AGE Inhibitory Activity

The synthesized compounds were assayed by the spectrofluorometric method for inhibition of glucose-mediated protein glycation. All the compounds and aminoguanidine (positive control) with different concentrations were evaluated in triplicate [74].

##### Free Radical Scavenging Activity

The activity was tested using the spectrophotometric Blois method [75].

#### 3.5.2. In Vivo Studies

##### Animals

Wistar rats (200−250 g, either sex) were used for in vivo evaluation. The protocol was approved by IAEC, Chitkara University, Punjab, India (approval number: IAEC/CCP/22/01/PR-09).

##### Dementia Induction

To induce cognitive impairment in rats, solutions of Streptozotocin (STZ, 3 mg/kg) in artificial cerebro-spinal fluid (ACSF) were given as i.c.v. (intracerebroventricular) injections. The STZ injections were administered bilaterally on days 1 and 3 of the experimental protocol. The amount of STZ injected was adjusted to ensure that 10 µL was delivered to each site. A control group of rats was given i.c.v. injections of ACSF [76].

##### Experimental Groups

**Figure 9:**
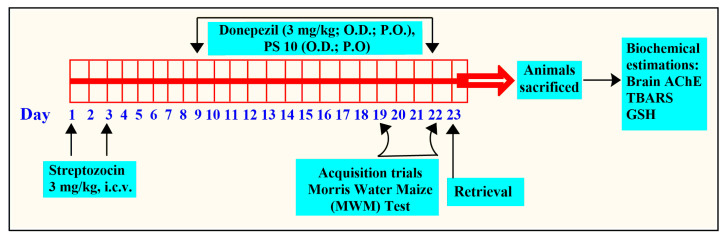
The flow of experimental protocol for in vivo studies.

The rats used in the study were divided into six groups (n = 6). The treatment and cognitive investigation in the rats were performed as shown in Figure 9.

Group I (ACSF): Intracerebroventricular injections of ASCF (10 µL) on day 1 and day 3.

Group II (STZ + vehicle): On days 1 and 3, group II was given intracerebroventricular injections of STZ (3 mg/kg, 10 µL).

Group III (STZ + Donepezil): Consisted of STZ-treated rats that received donepezil (5 mg/kg; *p.o.*) from day 9 to day 22 [77].

Groups IV, V, and VI (STZ + **PS10**): Following intracerebroventricular injections of STZ, animal groups were administered different doses of **PS10** (2, 5, and 10 mg/kg, *p.o*., respectively), from 9 to 22 days.

##### Behavioral Studies Using MWM (Morris Water Maze)

The MWM test was utilized by researchers to note the cognitive behavior of rats in terms of memory and learning [78]. The rats were trained from day 19 to 22, four times daily, to locate a hidden platform, and the time to locate the hidden platform was noted as ELT (escape latency time). Treatment with either donepezil or **PS10** was administered 60 min before the training session. On day 23, the platform was deleted, and the same procedure was performed to test animal memory by noting the time spent by the rats as the target quadrant (TSTQ), with a limit of 120 s [79].

##### Brain Biochemical Estimations

After the behavioral experiment was completed, the rats were euthanized under mild anesthesia using cervical dislocation, and their brains were extracted. The homogenates were made in a buffer (pH 7.4) for further analysis. AChE activity [58], total protein levels [80], reduced GSH levels, and thiobarbituric acid reactive species (TBARS) levels [81] were measured using the brain homogenates.

### 3.6. Molecular Dynamic Simulation Studies

Using the Schrodinger suite 2021-1’s Desmond module and a Linux (Ubuntu) operating system, the molecular dynamics (MD) simulation was executed with the aid of an NVIDIA Quadro K2200 graphics card. A simulation period of 100 ns was conducted to investigate the thermodynamic stability of **PS10** complexed with the AChE enzyme [82]. For stimulatory studies, the complex was built with the TIP3P explicit solvent system and centered with an orthorhombic periodic boundary box, followed by pH adjustment by Na^+^ and 0.15 M salt concentration. The built complex system minimization was carried out with the OPLSE_2005 force field. The NPT ensemble was a simulator with a fixed temperature of 300 K using the Nosé–Hoover Chain method, 1.0 fs time step, and 1.01325 bar. In order to investigate the trajectories, the simulation interaction graphs were constructed with a trajectory path of 4.8 and an energy interval of 1.2 ps. During the simulation, the structural dynamic patterns of the protein–ligand complex were assessed as the RMSD (root mean square deviation) for both components of the complex. The RMSD plot recorded the average change in the displacement of the backbone atoms of the protein and ligand structures. Additionally, the root mean square fluctuations (RMSF) for the protein and ligand in the complex were also computed and plotted the extent of the flexibility of each component. The RMSF represents the minimized fluctuations of the protein–ligand complex.

## 4. Conclusions

In this study, the objective was to assess the potential of the designed novel aminoethyl-O-chalcone-based derivatives having multi-functional potential against AD. The compounds were evaluated using in silico, in vitro, and in vivo assays to determine their activity against varied targets like AChE, AGE formation, and free radical scavenging. The design of the derivatives was based on the structural aspects of the biogenic molecule acetylcholine and the biological atmosphere within the active site of the AChE enzyme. The in silico pharmacokinetic profiles of the synthesized compounds revealed their drug-likeliness with good brain penetrability and gastrointestinal absorptivity. Most of the compounds exhibited significant inhibitory activities against AChE and AGE formation with additional antioxidant properties. The SAR analysis suggested that the potential for AChE inhibition was mainly attributed to the type of amino group present in the compounds. The compound **PS10** with *N*-methylpiperazine substitution exhibited considerably higher AChE inhibitory potential than donepezil. The compound **PS5** also exhibited some significant inhibition of AChE with respect to the reference. Additionally, the docking analysis showed that *N*-methylpiperazine (**PS10**) and piperidine (**PS5**) interacted with the crucial amino acids of the active site of the AChE enzyme. Moreover, these compounds had significant free radical scavenging ability and AGE product formation inhibition. Among all the synthesized aminoethyl-*O*-chalcone derivatives, **PS5** and **PS10** were found to be competent multifunctional molecules. The biochemical estimations also showed significant reductions in TBARS and GSH (oxidative stress markers) and AChE activities. These multifunctional aspects make these newly designed and synthesized compounds potential candidates for the development of drugs for AD and could be further explored to manage AD.

## Figures and Tables

**Table 1:** ADME profile and the physicochemical parameters of the aminoethyl-*O*-chalcone derivatives.

S. No.	Compound	Mol.Wt.	HB Acc	HB Don	TPSA	Consensus Log P	Ali Log S	Rule of Five	Brain Permeant	GI Absorption
1	**PS-2**	295.38	3	0	29.54	3.37	−3.38	0	Yes	High
2	**PS-3**	323.43	3	0	29.54	4.02	−4.14	0	Yes	High
3	**PS-4**	371.47	3	0	29.54	4.7	−5.56	0	Yes	High
4	**PS-5**	335.44	3	0	29.54	4.1	−4.88	0	Yes	High
5	**PS-6**	336.43	4	1	41.57	2.9	−3.58	0	Yes	High
6	**PS-7**	337.41	4	0	38.77	3.19	−3.82	0	Yes	High
7	**PS-8**	321.41	3	0	29.54	3.79	−4.52	0	Yes	High
8	**PS-9**	318.37	3	0	44.12	3.22	−4.24	0	Yes	High
9	**PS-10**	350.45	4	0	32.78	3.15	−3.88	0	Yes	High
10	**PS-11**	364.48	4	0	32.78	3.48	−4.26	0	Yes	High
11	**PS-12**	349.47	3	0	29.54	4.39	−5.26	0	Yes	High
12	**PS-13**	378.51	4	0	32.78	3.8	−4.62	0	Yes	High
13	**PS-14**	363.49	3	0	29.54	4.72	−5.63	0	Yes	High
14	**PS-15**	392.53	4	0	32.78	4.13	−4.99	0	Yes	High
15	**PS-16**	377.52	3	0	29.54	5.03	−6	0	Yes	High
16	**PS-17**	406.56	4	0	32.78	4.45	−5.37	0	Yes	High
17	**PS-18**	391.55	3	0	29.54	5.35	−6.37	0	Yes	High
18	**PS-19**	380.48	5	0	42.01	3.18	−4.04	0	Yes	High
19	**PS-20**	410.51	6	0	51.24	3.14	−4.21	0	Yes	High
20	**PS-21**	440.53	7	0	60.47	3.13	−4.37	0	Yes	High
21	**PS-22**	325.4	4	0	38.77	3.48	−4.17	0	Yes	High
22	**PS-23**	351.44	4	0	38.77	3.77	−4.68	0	Yes	High
23	**PS-24**	365.47	4	0	38.77	4.06	−5.06	0	Yes	High
24	**PS-25**	366.45	5	1	50.8	2.86	−3.74	0	Yes	High
25	**PS-26**	380.48	5	0	42.01	3.18	−4.04	0	Yes	High
26	**PS-27**	295.38	3	0	29.54	3.37	−3.38	0	Yes	High
27	**PS-28**	353.45	4	0	38.77	4.1	−4.91	0	Yes	High
28	**PS-29**	379.49	4	0	38.77	4.37	−5.42	0	Yes	High
29	**PS-30**	339.43	4	0	38.77	3.78	−4.55	0	Yes	High
30	**PS-31**	379.49	4	0	38.77	4.43	−5.42	0	Yes	High
31	**PS-32**	353.45	4	0	38.77	4.14	−4.93	0	Yes	High
32	**PS-33**	367.44	5	0	48	3.2	−3.98	0	Yes	High
33	**PS-34**	339.43	4	0	38.77	3.8	−4.56	0	Yes	High
34	**PS-35**	387.47	3	0	38.77	4.68	−6.02	0	Yes	High
35	**PS-36**	367.48	4	0	38.77	4.42	−5.3	0	Yes	High
36	**PS-37**	379.49	4	0	38.77	4.3	−5.5	0	Yes	High
37	**PS-38**	381.46	5	0	48	3.52	−4.35	0	Yes	High
38	**PS-39**	353.45	4	0	38.77	4.1	−4.92	0	Yes	High
39	**PS-40**	337.41	4	0	38.77	3.71	−4.79	0	Yes	High
40	**PS-41**	353.45	4	0	38.77	4.16	−5.1	0	Yes	High

**Table 2:** The docking scores with their interactions with enzyme **4**.

Compound ID	Structure	Docking Score (Kcal/mol)	Interactions
**PS1**	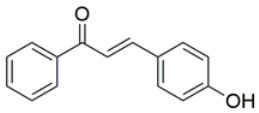	−31.90	His440, Ser200, Tyr121, Trp279
**PS2**	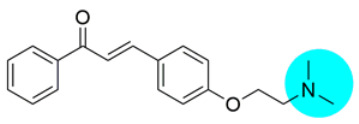	−49.39	His440, Trp84, Trp279, Ser200, Tyr121, Arg289, Gly119
**PS3**	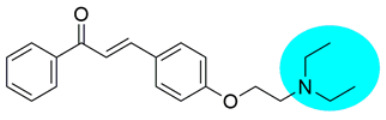	−51.43	His440, Ser200, Trp84, Trp279, Tyr121, Arg289, Gly119
**PS4**	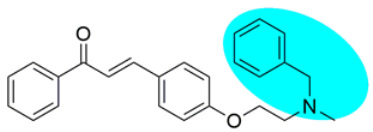	−56.92	Trp84, Phe330, Trp279, Arg289, Tyr130, His440, Ser200, Gly119
**PS5**	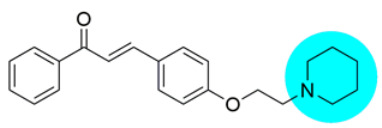	−52.28	His440, Phe330, Trp84, Trp279, Arg289, Ser200, Tyr121, Gly119, Asp72
**PS6**	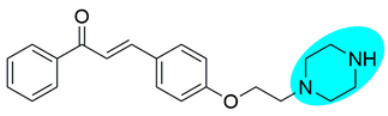	−51.08	His440, Trp84, Phe330, Arg289, Ser200, Tyr121, Trp279, Gly119
**PS7**	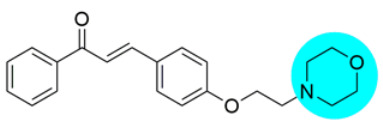	−51.01	Trp84, Ph330, Trp279, His440, Ser 200, Tyr121, Gly119, Arg289
**PS8**	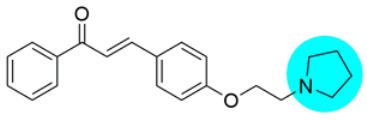	−52.83	His440, Trp84, Phe330, Ser200, Tyr121, Trp279, Arg289
**PS9**	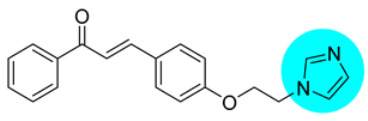	−53.45	His440, Trp279, Trp84, Ser200, Tyr121, Phe330
**PS10**	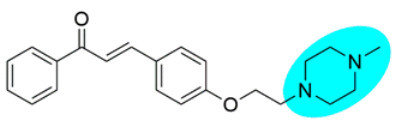	−63.75	His440, Asp72, Trp279, Trp84, Ser200, Tyr121, Arg289, Phe330
**Donepezil**	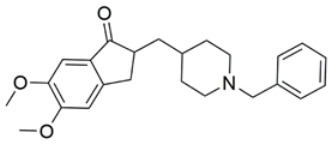	−51.89	Trp84, Tyr121, His440, Ser200, Trp279, Phe330

**Table 3:** The AChE inhibition, AGE formation inhibition, and radical scavenging activities.

	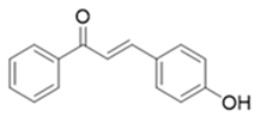		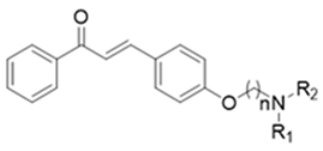	
PS1			PS (2–10)	
C. No.	-NR_1_R_2_	AChE Inhibitory Activity (Mean ^n^ ± SD, nM)	AGE Inhibitory Activity (Mean ^n^ ± SD, µM)	Radical Scavenging Activity (Mean ^n^ ± SD, nM)
**PS1**	-	36.47 ± 0.42	43.6 ± 0.08	22.41 ± 0.4
**PS2**	Dimethylamino	52.51 ± 0.20	68.6 ± 0.23	49.85 ± 0.11
**PS3**	Diethylamino	47.5 ± 0.21	67.3 ± 0.33	49.41 ± 0.22
**PS4**	N-benzyl(methyl)amino	48.8 ± 0.04	69.39 ± 0.2	49.48 ± 0.21
**PS5**	Piperidine	19.71 ± 0.3	55.3 ± 0.2	34.38 ± 0.4
**PS6**	Piperazine	21.4 ± 0.8	64.51 ± 0.2	46.7 ± 0.24
**PS7**	Morpholine	22.72 ± 0.13	51.2 ± 0.8	37.65 ± 0.24
**PS8**	Pyrrolidine	32.72 ± 0.12	65.41 ± 0.36	48.64 ± 0.18
**PS9**	Imidazole	26.3 ± 0.3	65.7 ± 0.17	48.58 ± 0.42
**PS10**	4-Methylpiperazine	15.3 ± 0.72	49.85 ± 0.13	22.83 ± 0.2
**Std.**	Donepezil	15.68 ± 0.26	--	--
**Std.**	Ascorbic acid	--	--	21.7 ± 0.08
**Std.**	Aminoguanidine (AG)	--	44.3 ± 0.12	--

n = 3.

## Data Availability

The data presented in this study are available within the article.

## Supplementary Materials

The following supporting information can be downloaded at: https://www.mdpi.com/article/10.3390/molecules28186579/s1.
