# Supplementary material for: Recent Development of Novel Aminoethyl-Substituted Chalcones as Potential Drug Candidates for the Treatment of Alzheimer’s Disease
Source: Molecules. 2023 Sep 12;28(18):6579. doi: 10.3390/molecules28186579 (PMC10534526; doi:10.3390/molecules28186579)
Supplement: Supplementary file 1 [file molecules-28-06579-s001.zip › molecules-2593825-SI.pdf]

Discovery and development of aminoethyl-O-chalcones as potential multifuctional agents against Alzheimer's disease

PS1

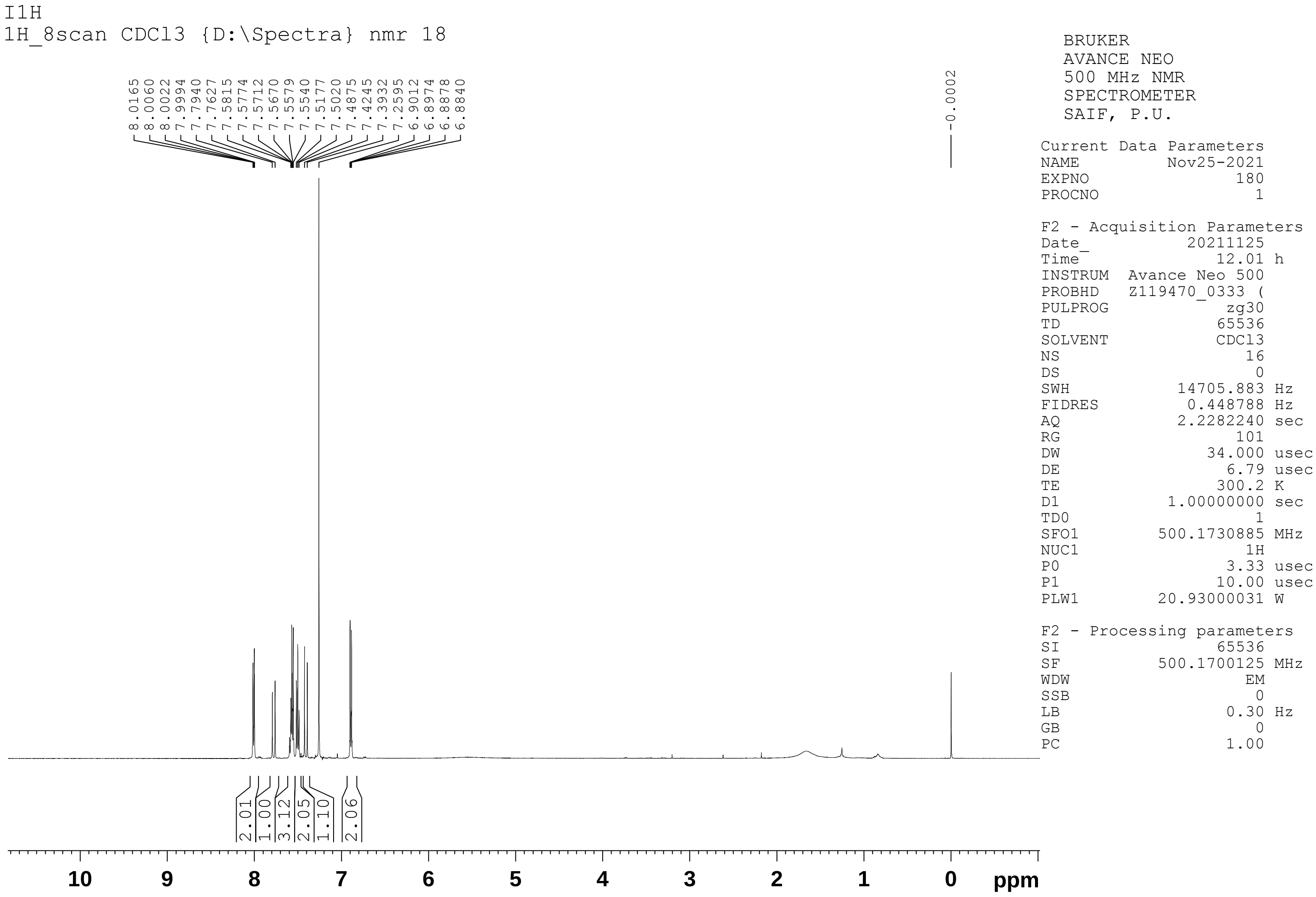

I1H
1H_8scan CDCl3 {D:\Spectra} nmr 18

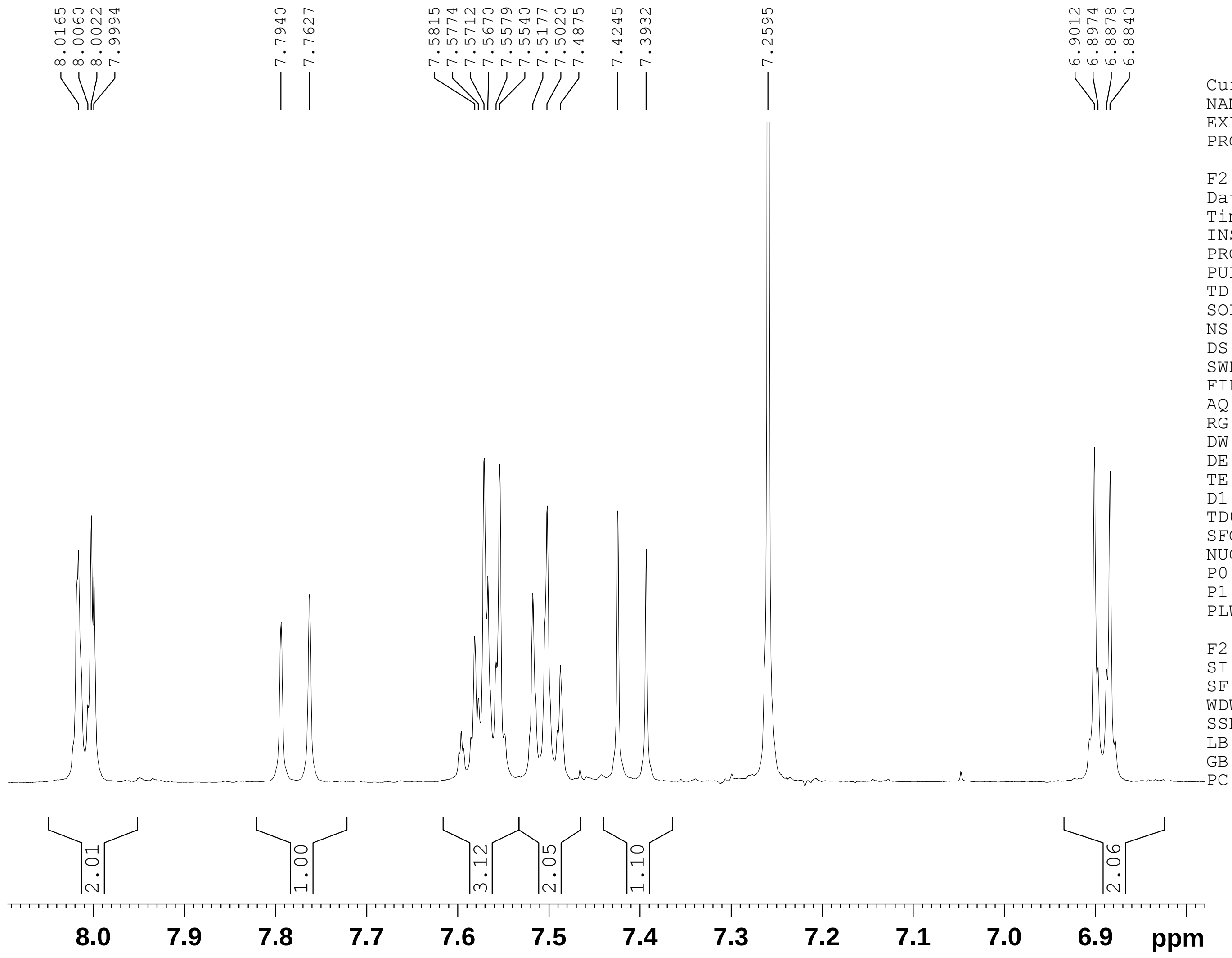

BRUKER
AVANCE NEO
500 MHz NMR
SPECTROMETER
SAIF, P.U.

Current Data Parameters

NAME	Nov25-2021	
EXPNO	180	
PROCNO	1	

F2 - Acquisition Parameters

Date_	20211125	
Time	12.01	h
INSTRUM	Avance Neo 500	
PROBHD	Z119470_0333 (	
PULPROG	zg30	
TD	65536	
SOLVENT	CDCl3	
NS	16	
DS	0	
SWH	14705.883	Hz
FIDRES	0.448788	Hz
AQ	2.2282240	sec
RG	101	
DW	34.000	usec
DE	6.79	usec
TE	300.2	K
D1	1.00000000	sec
TD0	1	
SFO1	500.1730885	MHz
NUC1	1H	
P0	3.33	usec
P1	10.00	usec
PLW1	20.93000031	W

F2 - Processing parameters

SI	65536	
SF	500.1700125	MHz
WDW	EM	
SSB	0	
LB	0.30	Hz
GB	0	
PC	1.00	

I1H
1H_8scan CDCl3 {D:\Spectra} nmr 18

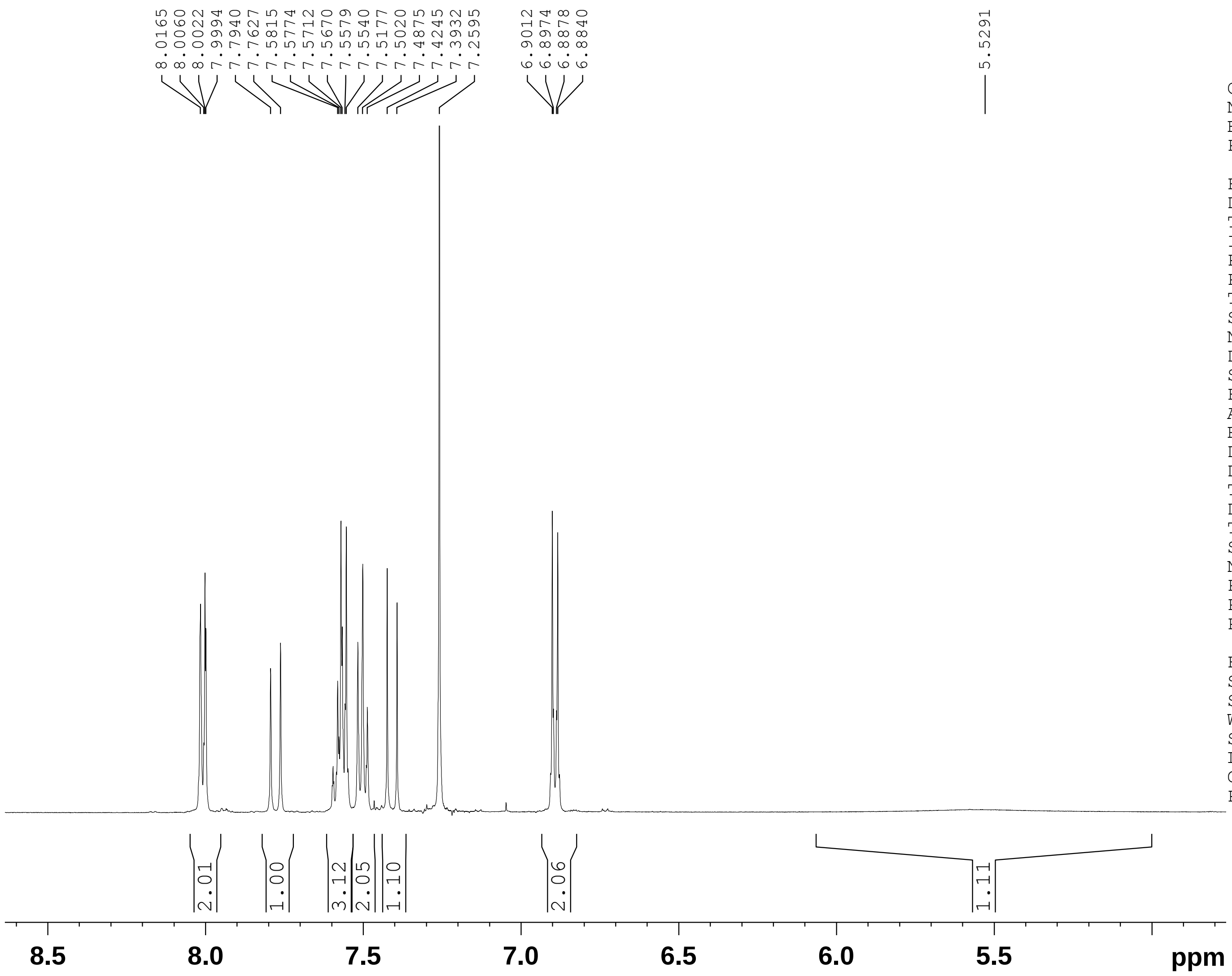

BRUKER
AVANCE NEO
500 MHz NMR
SPECTROMETER
SAIF, P.U.

Current Data Parameters
NAME Nov25-2021
EXPNO 180
PROCNO 1

F2 - Acquisition Parameters
Date_ 20211125
Time 12.01 h
INSTRUM Avance Neo 500
PROBHD Z119470_0333 (
PULPROG zg30
TD 65536
SOLVENT CDCl3
NS 16
DS 0
SWH 14705.883 Hz
FIDRES 0.448788 Hz
AQ 2.2282240 sec
RG 101
DW 34.000 usec
DE 6.79 usec
TE 300.2 K
D1 1.00000000 sec
TD0 1
SFO1 500.1730885 MHz
NUC1 1H
P0 3.33 usec
P1 10.00 usec
PLW1 20.93000031 W

F2 - Processing parameters
SI 65536
SF 500.1700125 MHz
WDW EM
SSB 0
LB 0.30 Hz
GB 0
PC 1.00

I1H
1H_8scan CDCl3 {D:\Spectra} nmr 18

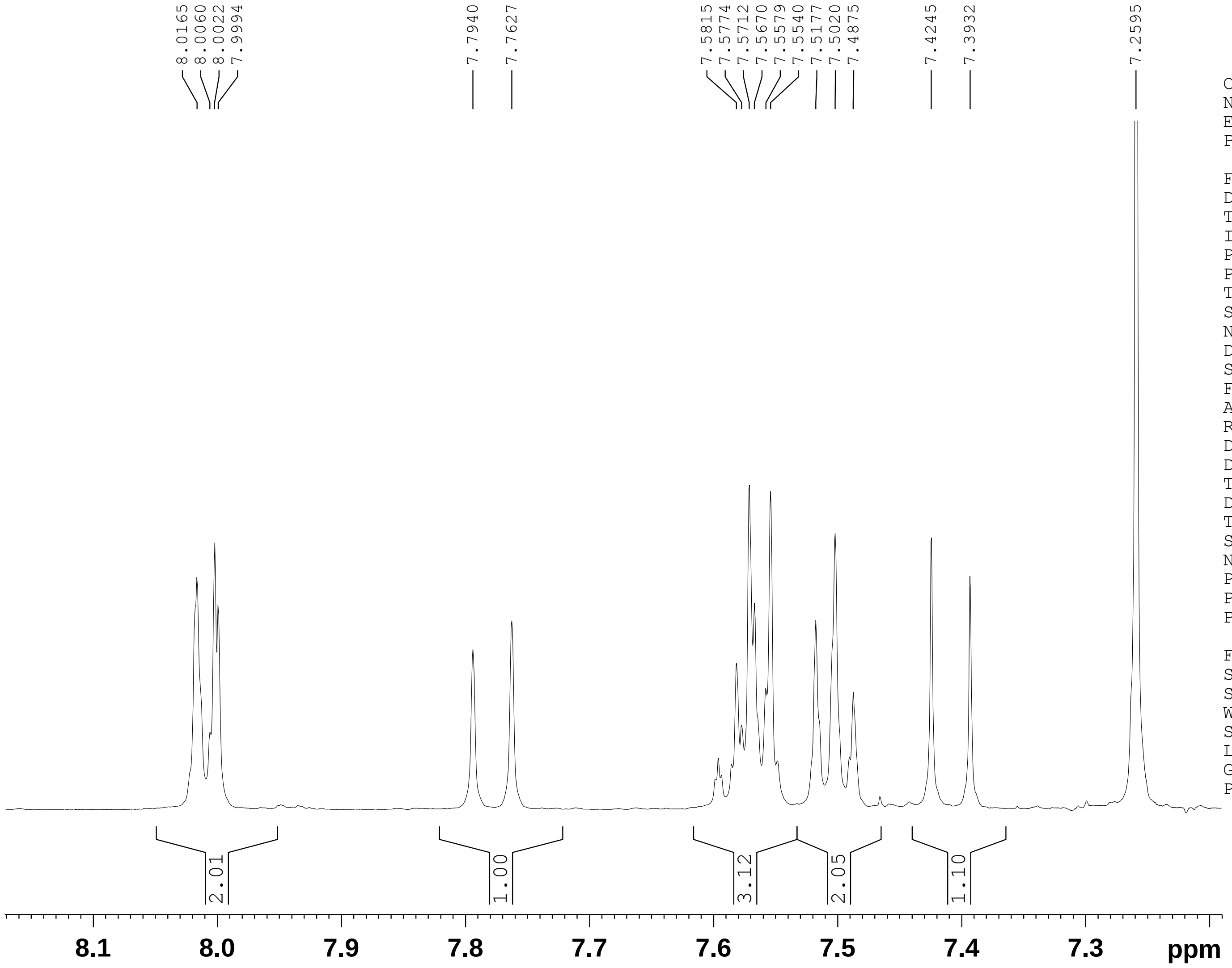

BRUKER
AVANCE NEO
500 MHz NMR
SPECTROMETER
SAIF, P.U.

Current Data Parameters

NAME	Nov25-2021
EXPNO	180
PROCNO	1

F2 - Acquisition Parameters

Date_	20211125
Time	12.01 h
INSTRUM	Avance Neo 500
PROBHD	Z119470_0333 (
PULPROG	zg30
TD	65536
SOLVENT	CDCl3
NS	16
DS	0
SWH	14705.883 Hz
FIDRES	0.448788 Hz
AQ	2.2282240 sec
RG	101
DW	34.000 usec
DE	6.79 usec
TE	300.2 K
D1	1.00000000 sec
TD0	1
SFO1	500.1730885 MHz
NUC1	1H
P0	3.33 usec
P1	10.00 usec
PLW1	20.93000031 W

F2 - Processing parameters

SI	65536
SF	500.1700125 MHz
WDW	EM
SSB	0
LB	0.30 Hz
GB	0
PC	1.00

I1H
1H_8scan CDCl3 {D:\Spectra} nmr 18

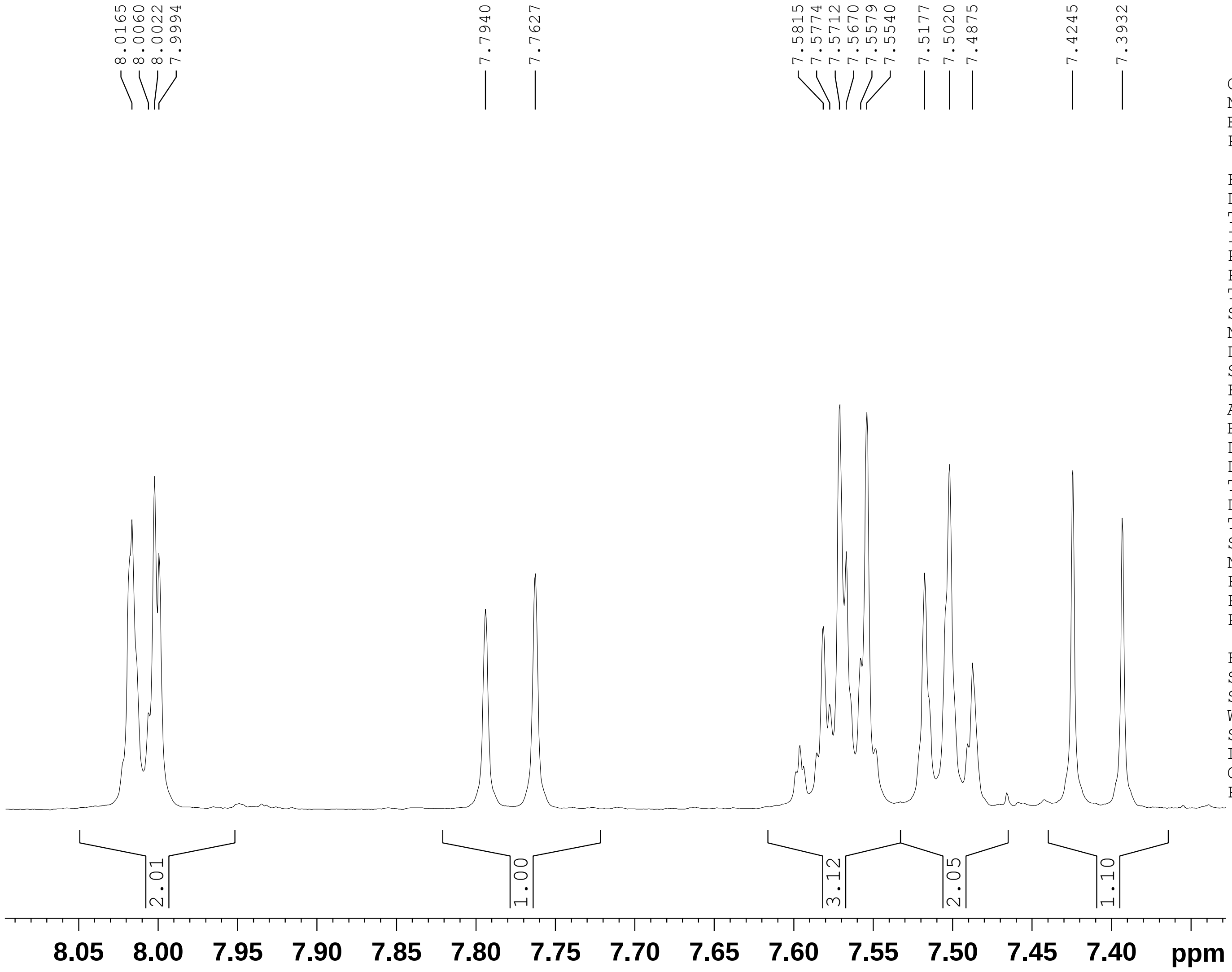

BRUKER
AVANCE NEO
500 MHz NMR
SPECTROMETER
SAIF, P.U.

Current Data Parameters
NAME Nov25-2021
EXPNO 180
PROCNO 1

F2 - Acquisition Parameters
Date_ 20211125
Time 12.01 h
INSTRUM Avance Neo 500
PROBHD Z119470_0333 (
PULPROG zg30
TD 65536
SOLVENT CDCl3
NS 16
DS 0
SWH 14705.883 Hz
FIDRES 0.448788 Hz
AQ 2.2282240 sec
RG 101
DW 34.000 usec
DE 6.79 usec
TE 300.2 K
D1 1.00000000 sec
TD0 1
SFO1 500.1730885 MHz
NUC1 1H
P0 3.33 usec
P1 10.00 usec
PLW1 20.93000031 W

F2 - Processing parameters
SI 65536
SF 500.1700125 MHz
WDW EM
SSB 0
LB 0.30 Hz
GB 0
PC 1.00

INH
C13CPD DMSO {D:\Spectra} nmr 4

BRUKER
AVANCE NEO
500 MHz NMR SPECTROMETER
SAIF, PANJAB UNIVERSITY,
CHANDIGARH

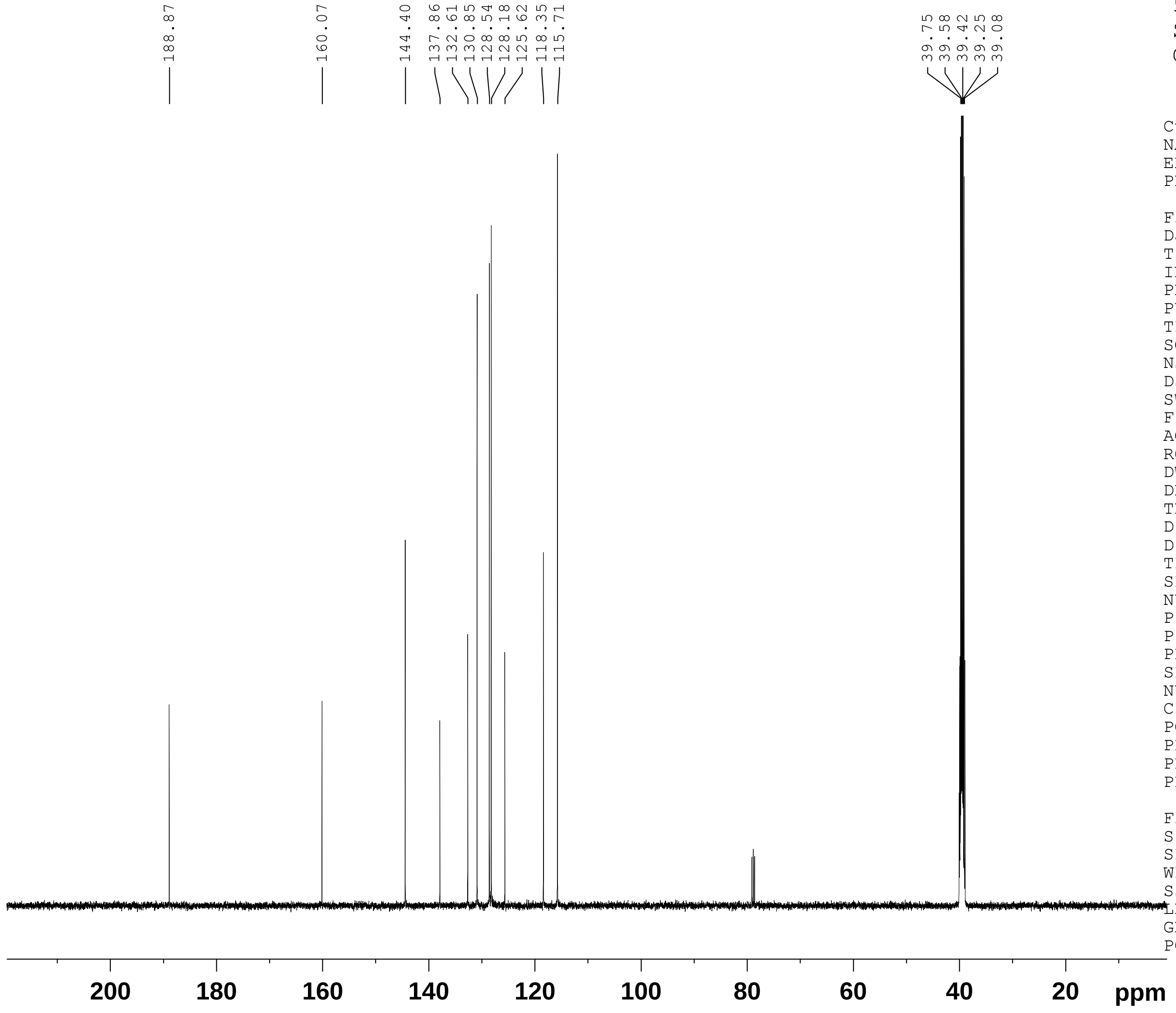

Current Data Parameters

NAME	Jan12-2022
EXPNO	42
PROCNO	1

F2 - Acquisition Parameters

Date_	20220113
Time	7.01 h
INSTRUM	Avance Neo 500
PROBHD	Z119470_0333 (
PULPROG	zgpg30
TD	65536
SOLVENT	DMSO
NS	512
DS	4
SWH	37037.035 Hz
FIDRES	1.130281 Hz
AQ	0.8847360 sec
RG	101
DW	13.500 usec
DE	6.50 usec
TE	300.1 K
D1	2.00000000 sec
D11	0.03000000 sec
TD0	1
SFO1	125.7804233 MHz
NUC1	13C
P0	3.33 usec
P1	10.00 usec
PLW1	83.14099884 W
SFO2	500.1720007 MHz
NUC2	1H
CPDPRG[2	waltz65
PCPD2	80.00 usec
PLW2	20.93000031 W
PLW12	0.32703000 W
PLW13	0.16449000 W

F2 - Processing parameters

SI	32768
SF	125.7679219 MHz
WDW	EM
SSB	0
LB	1.00 Hz
GB	0
PC	1.40

INH
C13CPD DMSO {D:\Spectra} nmr 4

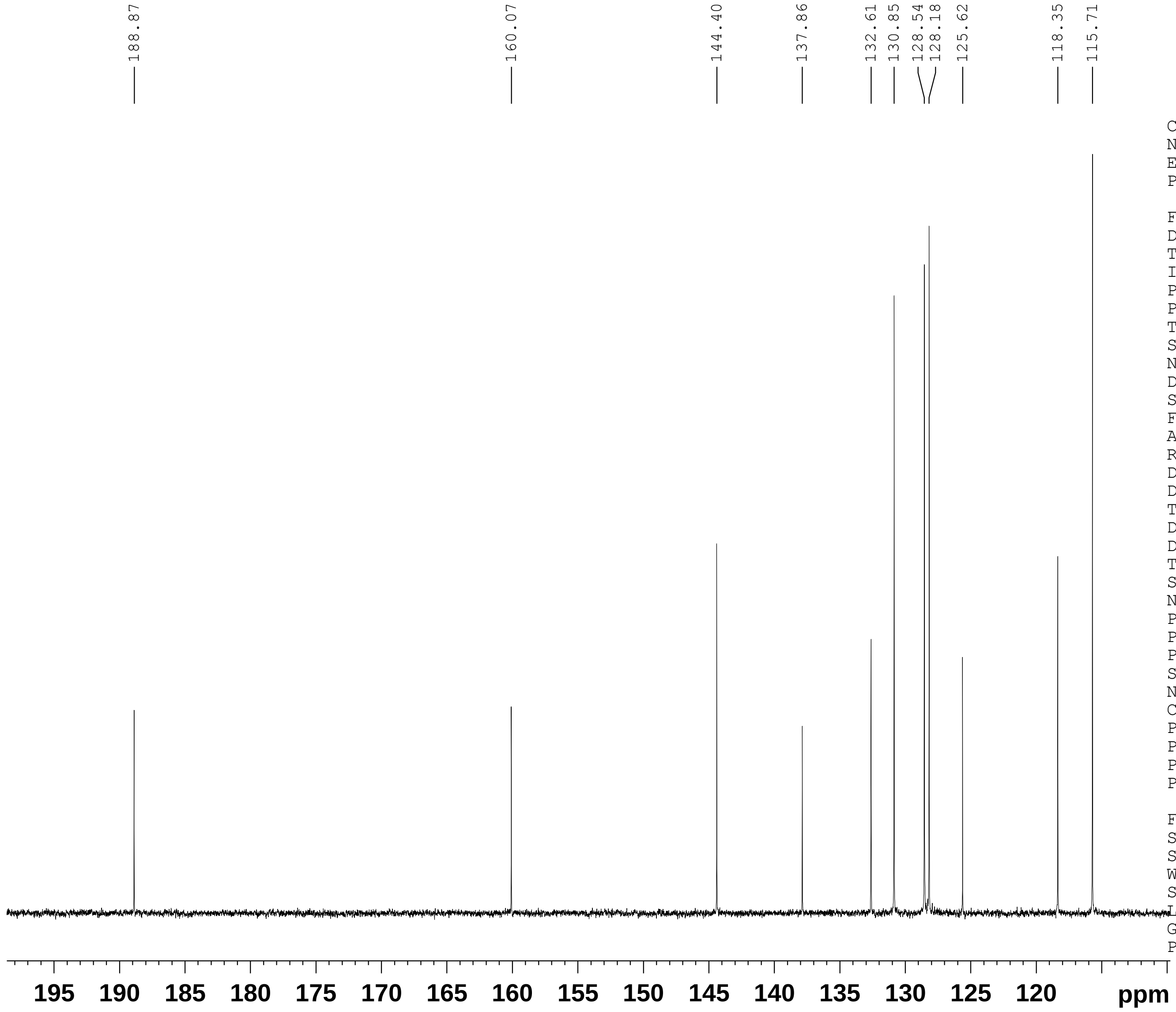

BRUKER
AVANCE NEO
500 MHz NMR SPECTROMETER
SAIF, PANJAB UNIVERSITY,
CHANDIGARH

Current Data Parameters

NAME	Jan12-2022	
EXPNO	42	
PROCNO	1	

F2 - Acquisition Parameters

Date_	20220113	
Time	7.01	h
INSTRUM	Avance Neo 500	
PROBHD	Z119470_0333 (	
PULPROG	zgpg30	
TD	65536	
SOLVENT	DMSO	
NS	512	
DS	4	
SWH	37037.035	Hz
FIDRES	1.130281	Hz
AQ	0.8847360	sec
RG	101	
DW	13.500	usec
DE	6.50	usec
TE	300.1	K
D1	2.00000000	sec
D11	0.03000000	sec
TD0	1	
SFO1	125.7804233	MHz
NUC1	13C	
P0	3.33	usec
P1	10.00	usec
PLW1	83.14099884	W
SFO2	500.1720007	MHz
NUC2	1H	
CPDPRG[2	waltz65	
PCPD2	80.00	usec
PLW2	20.93000031	W
PLW12	0.32703000	W
PLW13	0.16449000	W

F2 - Processing parameters

SI	32768	
SF	125.7679219	MHz
WDW	EM	
SSB	0	
LB	1.00	Hz
GB	0	
PC	1.40	

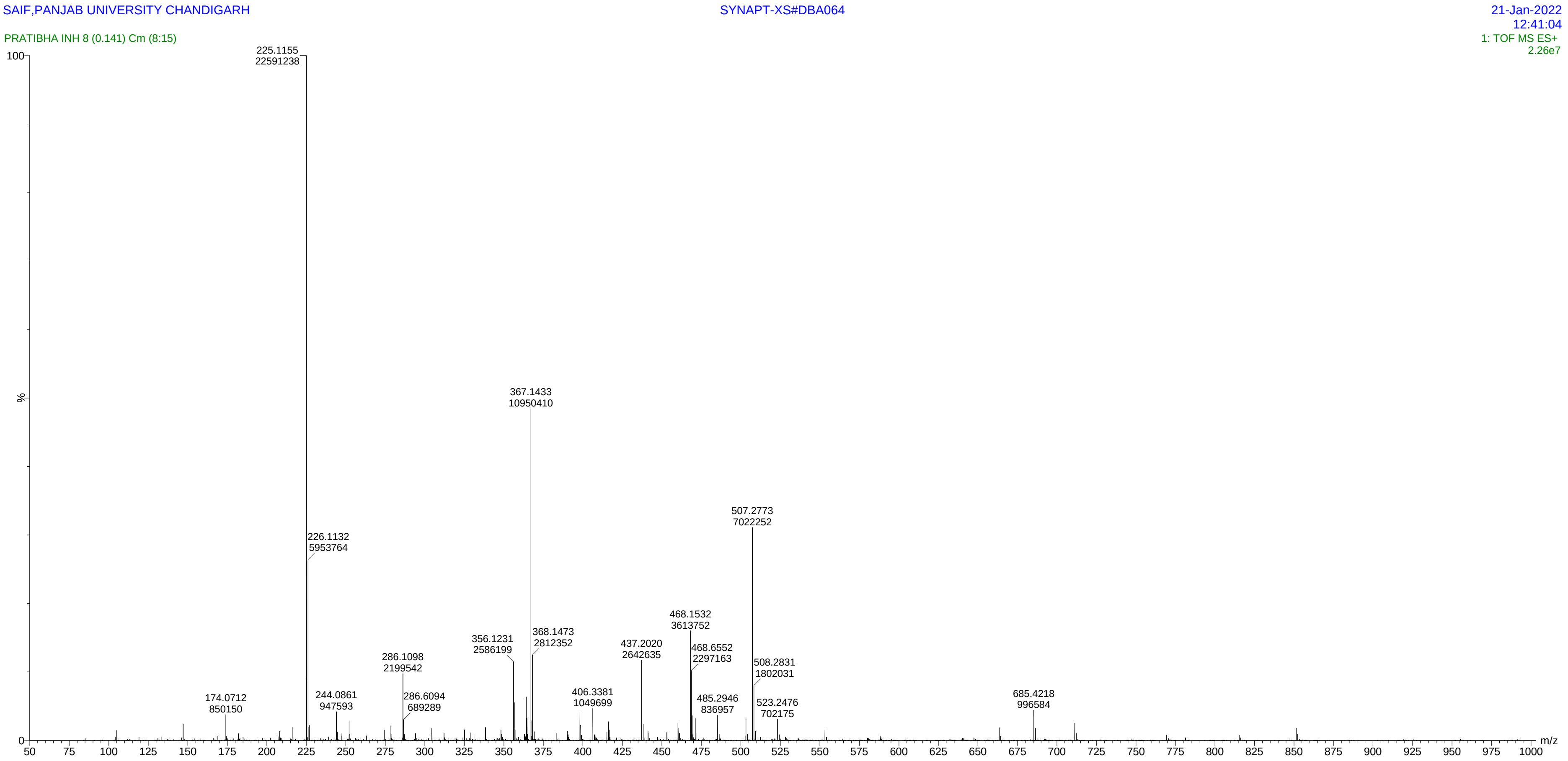

SAIF,PANJAB UNIVERSITY CHANDIGARH
SYNAPT-XS#DBA064
21-Jan-2022
12:41:04
PRATIBHA INH 8 (0.141) Cm (8:15)
1: TOF MS ES+
2.26e7
225.1155
22591238
367.1433
10950410
507.2773
7022252
226.1132
5953764
468.1532
3613752
368.1473
2812352
356.1231
2586199
437.2020
2642635
468.6552
2297163
286.1098
2199542
508.2831
1802031
244.0861
947593
286.6094
689289
406.3381
1049699
174.0712
850150
485.2946
836957
523.2476
702175
685.4218
996584
%
m/z

SAIF,PANJAB UNIVERSITY CHANDIGARH

SYNAPT-XS#DBA064

21-Jan-2022
12:41:04

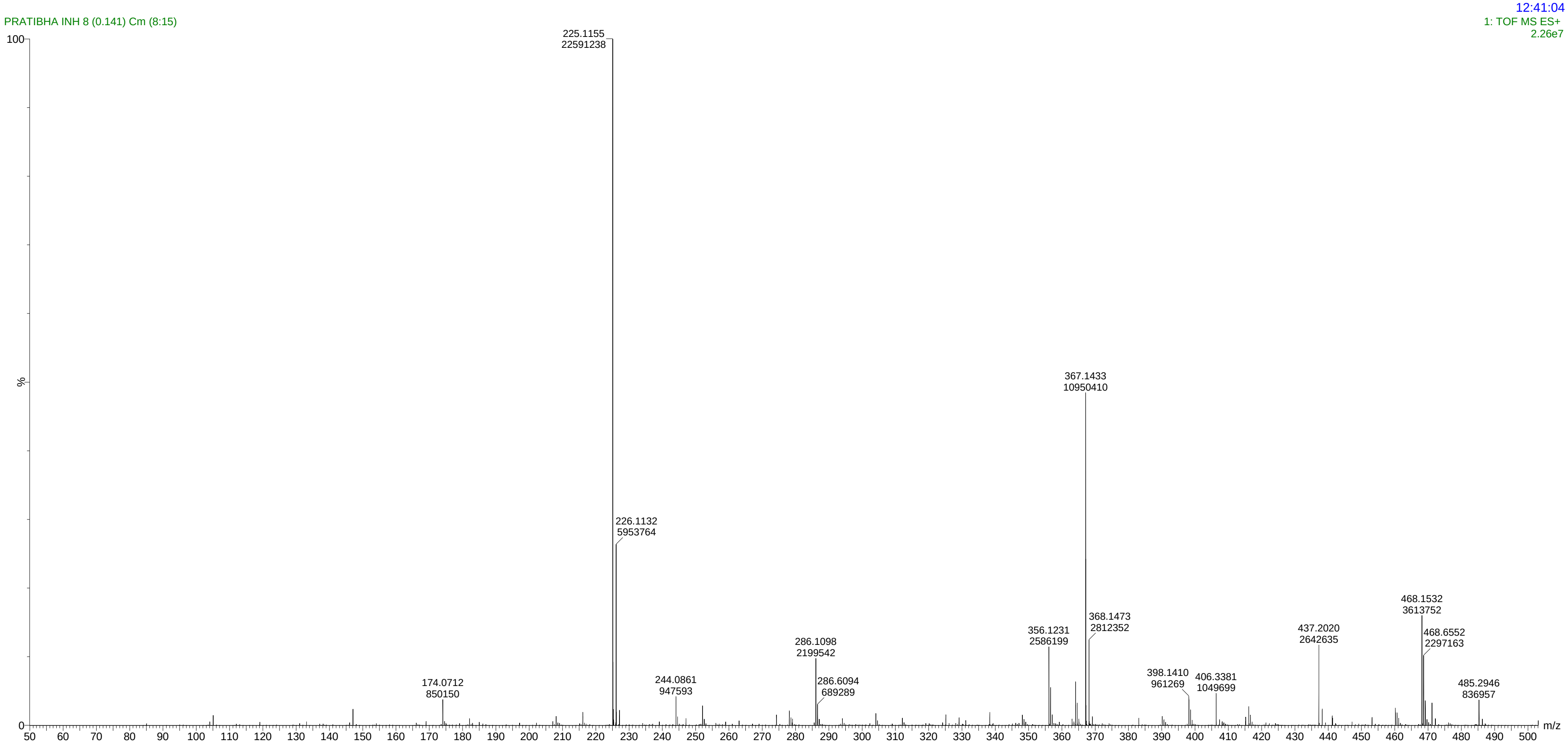

PS2

CH-O-1
1H_8scan CDCl3 {D:\Spectra} nmr 41

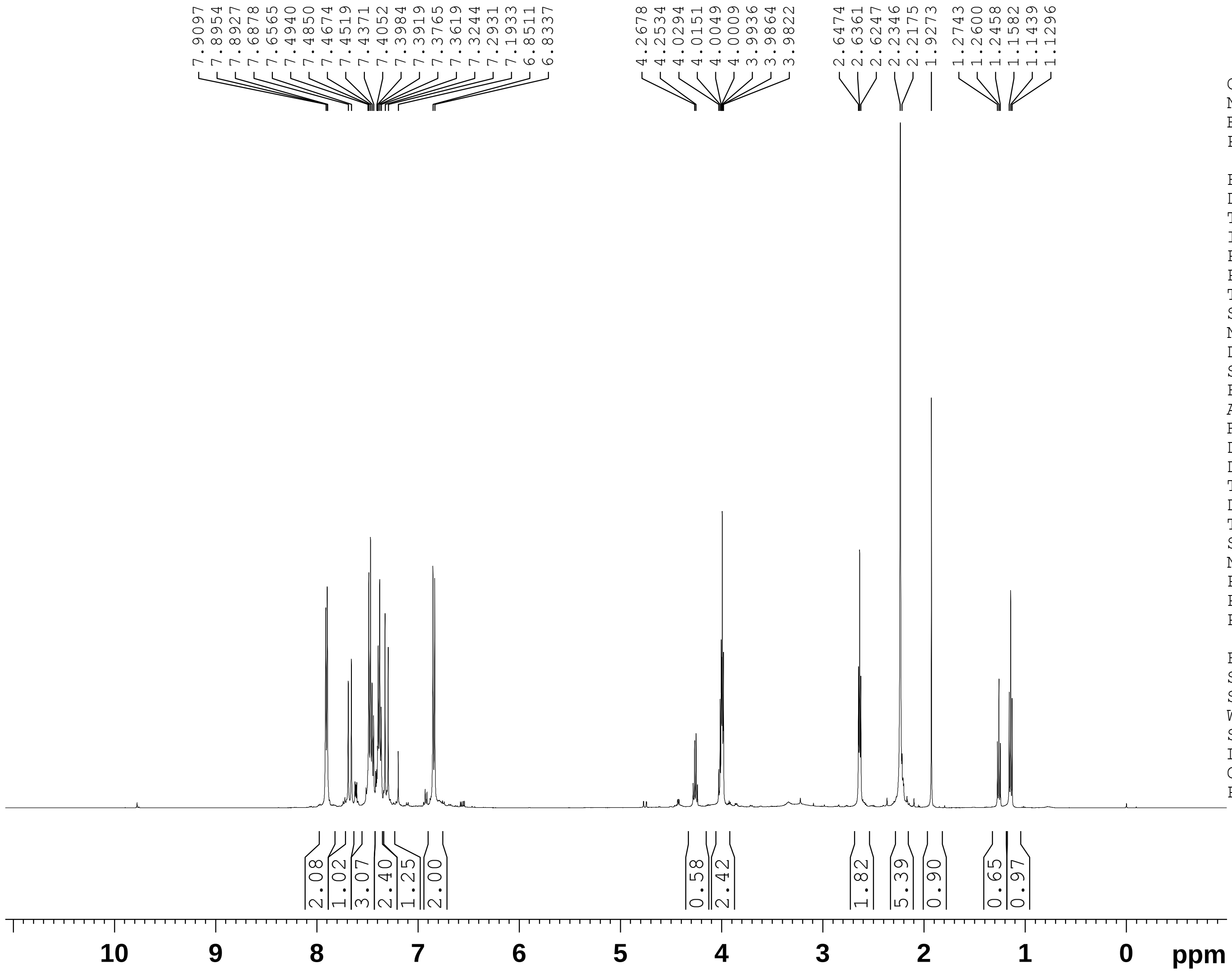

BRUKER
AVANCE NEO
500 MHz NMR
SPECTROMETER
SAIF, P.U.

Current Data Parameters

NAME	May05-2022
EXPNO	410
PROCNO	1

F2 - Acquisition Parameters

Date_	20220505
Time	15.56 h
INSTRUM	Avance Neo 500
PROBHD	Z119470_0333 (
PULPROG	zg30
TD	65536
SOLVENT	CDCl3
NS	16
DS	0
SWH	14705.883 Hz
FIDRES	0.448788 Hz
AQ	2.2282240 sec
RG	28.3797
DW	34.000 usec
DE	6.79 usec
TE	300.1 K
D1	1.00000000 sec
TD0	1
SFO1	500.1730885 MHz
NUC1	1H
P0	3.33 usec
P1	10.00 usec
PLW1	20.93000031 W

F2 - Processing parameters

SI	65536
SF	500.1700452 MHz
WDW	EM
SSB	0
LB	0.30 Hz
GB	0
PC	1.00

CH-O-1
1H_8scan CDCl3 {D:\Spectra} nmr 41

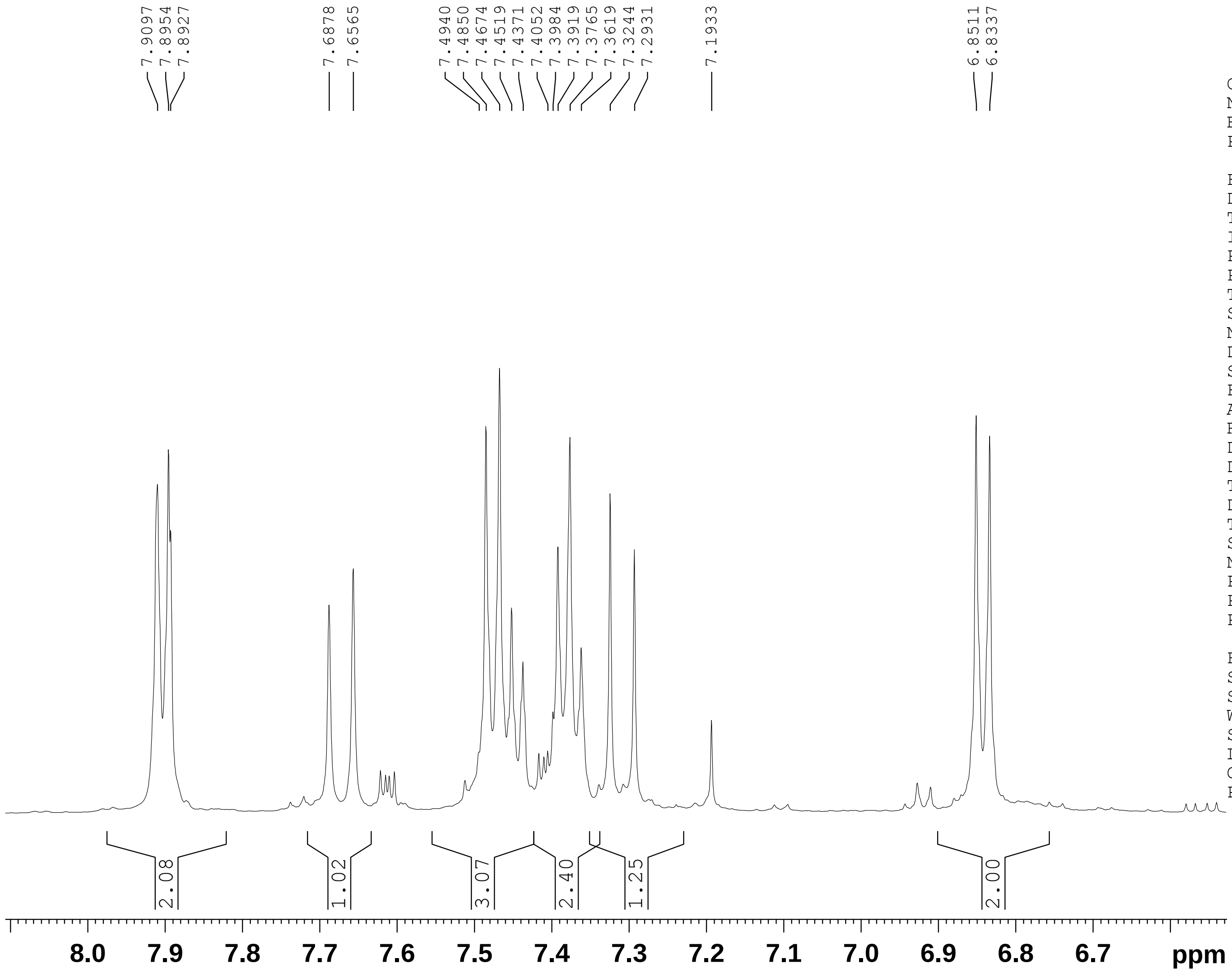

BRUKER
AVANCE NEO
500 MHz NMR
SPECTROMETER
SAIF, P.U.

Current Data Parameters

NAME	May05-2022
EXPNO	410
PROCNO	1

F2 - Acquisition Parameters

Date_	20220505
Time	15.56 h
INSTRUM	Avance Neo 500
PROBHD	Z119470_0333 (
PULPROG	zg30
TD	65536
SOLVENT	CDCl3
NS	16
DS	0
SWH	14705.883 Hz
FIDRES	0.448788 Hz
AQ	2.2282240 sec
RG	28.3797
DW	34.000 usec
DE	6.79 usec
TE	300.1 K
D1	1.00000000 sec
TD0	1
SFO1	500.1730885 MHz
NUC1	1H
P0	3.33 usec
P1	10.00 usec
PLW1	20.93000031 W

F2 - Processing parameters

SI	65536
SF	500.1700452 MHz
WDW	EM
SSB	0
LB	0.30 Hz
GB	0
PC	1.00

CH-O-1
1H_8scan CDCl3 {D:\Spectra} nmr 41

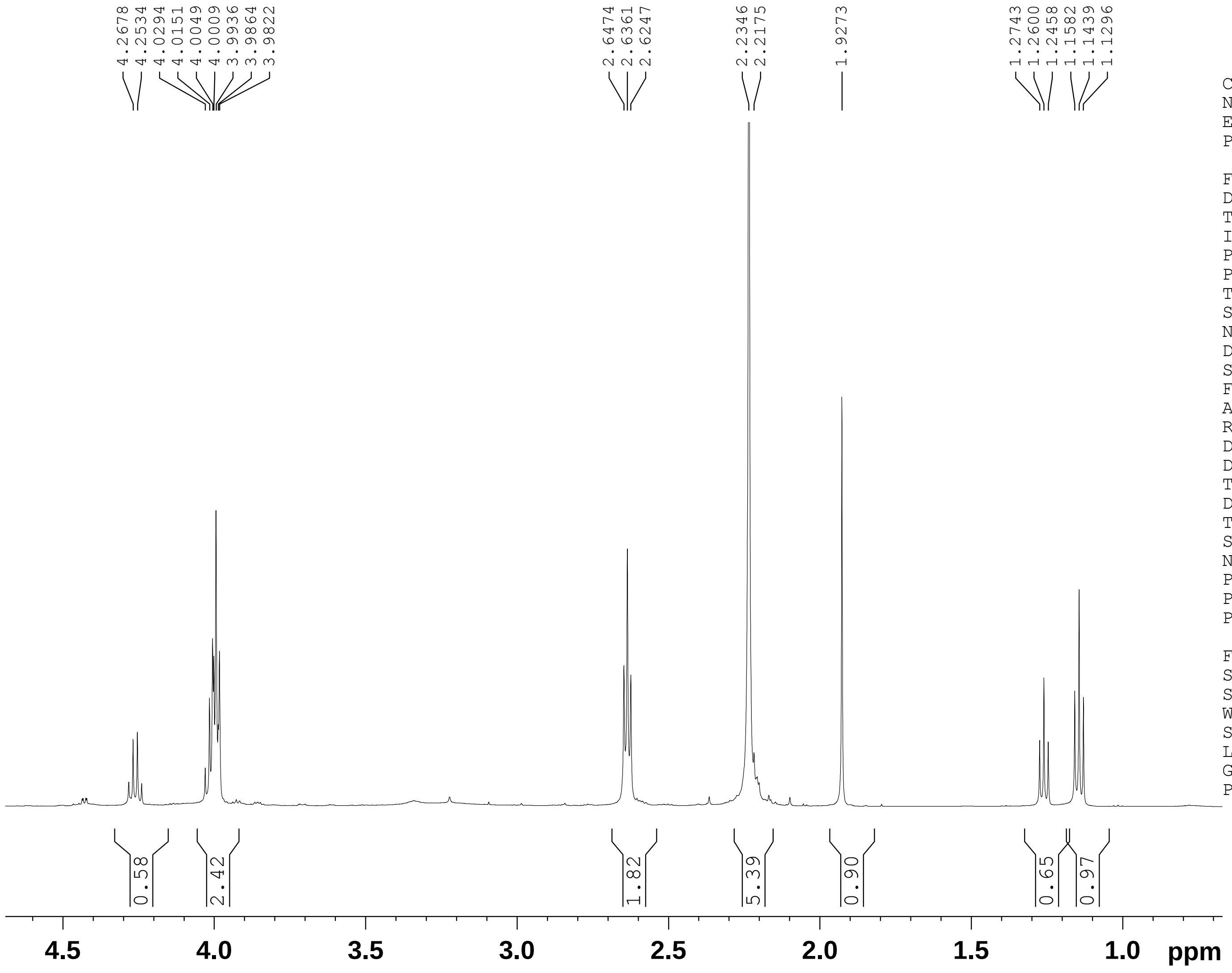

BRUKER
AVANCE NEO
500 MHz NMR
SPECTROMETER
SAIF, P.U.

Current Data Parameters

NAME	May05-2022
EXPNO	410
PROCNO	1

F2 - Acquisition Parameters

Date_	20220505
Time	15.56 h
INSTRUM	Avance Neo 500
PROBHD	Z119470_0333 (
PULPROG	zg30
TD	65536
SOLVENT	CDCl3
NS	16
DS	0
SWH	14705.883 Hz
FIDRES	0.448788 Hz
AQ	2.2282240 sec
RG	28.3797
DW	34.000 usec
DE	6.79 usec
TE	300.1 K
D1	1.00000000 sec
TD0	1
SFO1	500.1730885 MHz
NUC1	1H
P0	3.33 usec
P1	10.00 usec
PLW1	20.93000031 W

F2 - Processing parameters

SI	65536
SF	500.1700452 MHz
WDW	EM
SSB	0
LB	0.30 Hz
GB	0
PC	1.00

CH-O-PS3
C13CPD CDCl3 {D:\Spectra} nmr 25

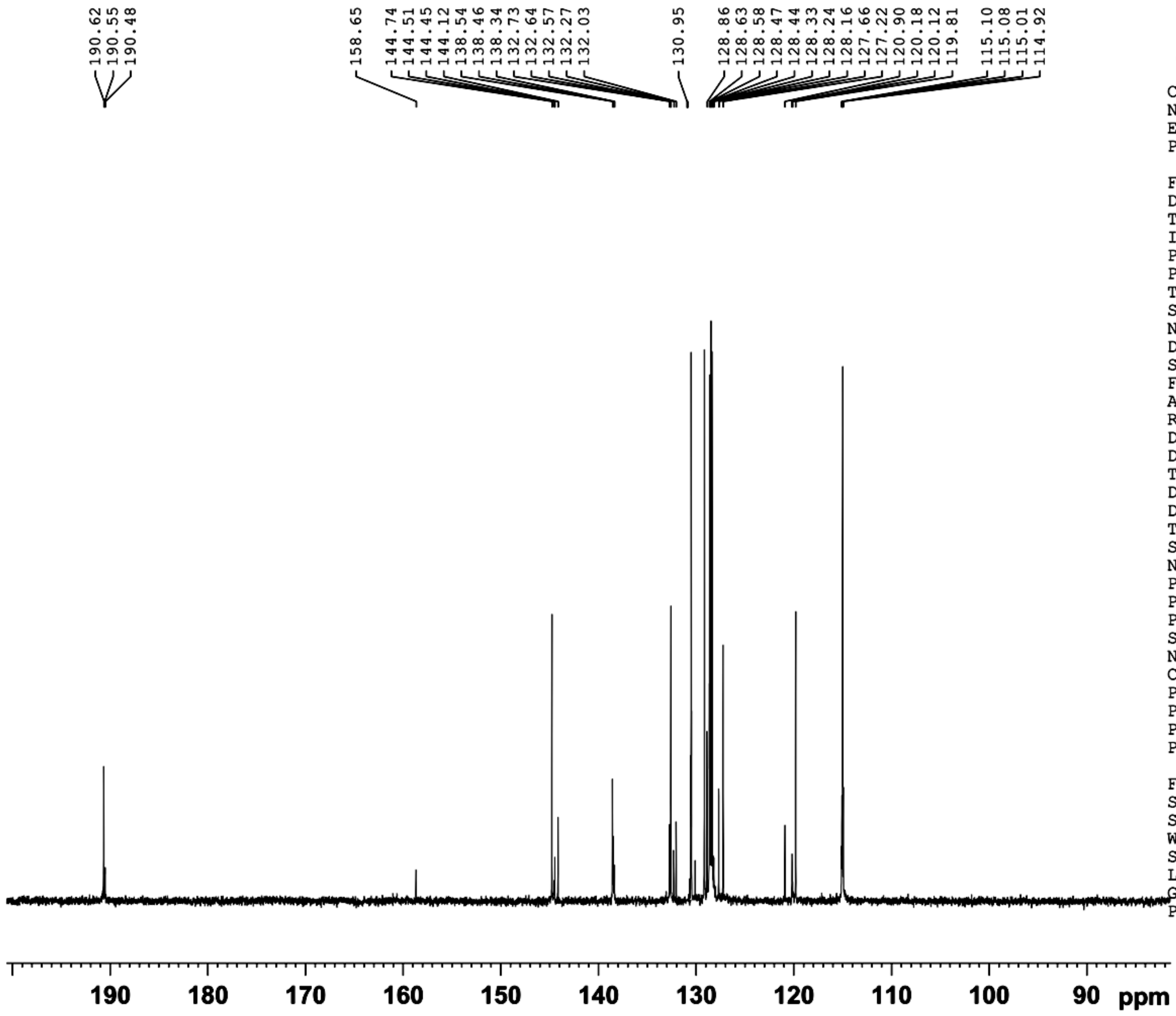

BRUKER
AVANCE NEO
500 MHz NMR SPECTROMETER
SAIF, PANJAB UNIVERSITY,
CHANDIGARH

Current Data Parameters

NAME	Mar31-2022	
EXPNO	251	
PROCNO	1	

F2 - Acquisition Parameters

Date_	20220331	
Time	17.35	h
INSTRUM	Avance Neo 500	
PROBHD	Z119470_0333 (	
PULPROG	zgpg30	
TD	65536	
SOLVENT	CDCl3	
NS	1024	
DS	4	
SWH	37037.035	Hz
FIDRES	1.130281	Hz
AQ	0.8847360	sec
RG	101	
DW	13.500	usec
DE	6.50	usec
TE	300.2	K
D1	2.00000000	sec
D11	0.03000000	sec
TD0	1	
SFO1	125.7804233	MHz
NUC1	13C	
P0	3.33	usec
P1	10.00	usec
PLW1	83.14099884	W
SFO2	500.1720007	MHz
NUC2	1H	
CPDPRG[2	waltz65	
PCPD2	80.00	usec
PLW2	20.93000031	W
PLW12	0.32703000	W
PLW13	0.16449000	W

F2 - Processing parameters

SI	32768	
SF	125.7678465	MHz
WDW	EM	
SSB	0	
LB	1.00	Hz
GB	0	
PC	1.40	

CH-O-PS3
C13CPD CDCl3 {D:\Spectra} nmr 25

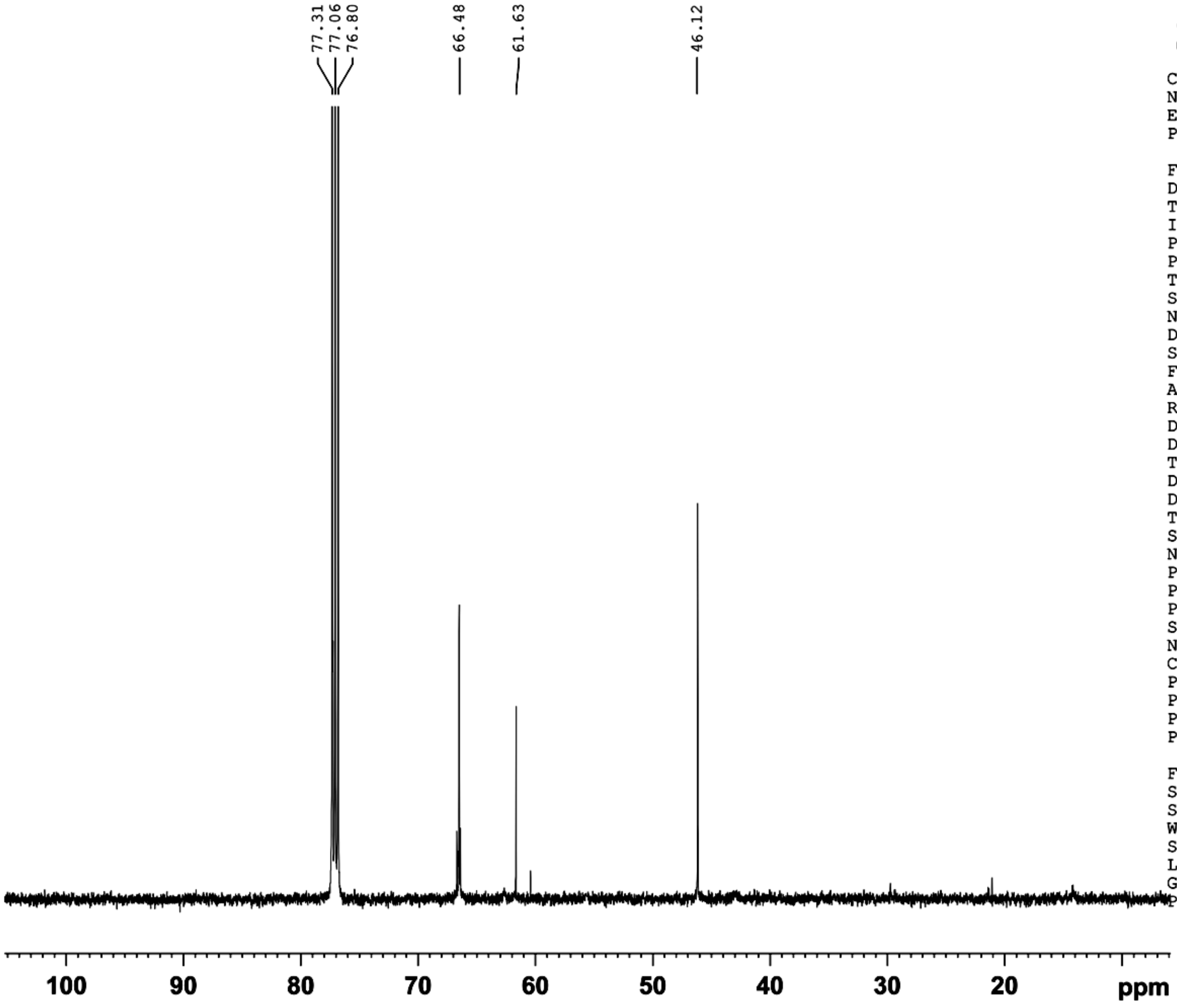

BRUKER
AVANCE NEO
500 MHz NMR SPECTROMETER
SAIF, PANJAB UNIVERSITY,
CHANDIGARH

Current Data Parameters
NAME Mar31-2022
EXPNO 251
PROCNO 1

F2 - Acquisition Parameters
Date_ 20220331
Time 17.35 h
INSTRUM Avance Neo 500
PROBHD Z119470_0333 (
PULPROG zgpg30
TD 65536
SOLVENT CDCl3
NS 1024
DS 4
SWH 37037.035 Hz
FIDRES 1.130281 Hz
AQ 0.8847360 sec
RG 101
DW 13.500 usec
DE 6.50 usec
TE 300.2 K
D1 2.00000000 sec
D11 0.03000000 sec
TD0 1
SFO1 125.7804233 MHz
NUC1 13C
P0 3.33 usec
P1 10.00 usec
PLW1 83.14099884 W
SFO2 500.1720007 MHz
NUC2 1H
CPDPRG[2 waltz65
PCPD2 80.00 usec
PLW2 20.93000031 W
PLW12 0.32703000 W
PLW13 0.16449000 W

F2 - Processing parameters
SI 32768
SF 125.7678465 MHz
WDW EM
SSB 0
LB 1.00 Hz
GB 0
PC 1.40

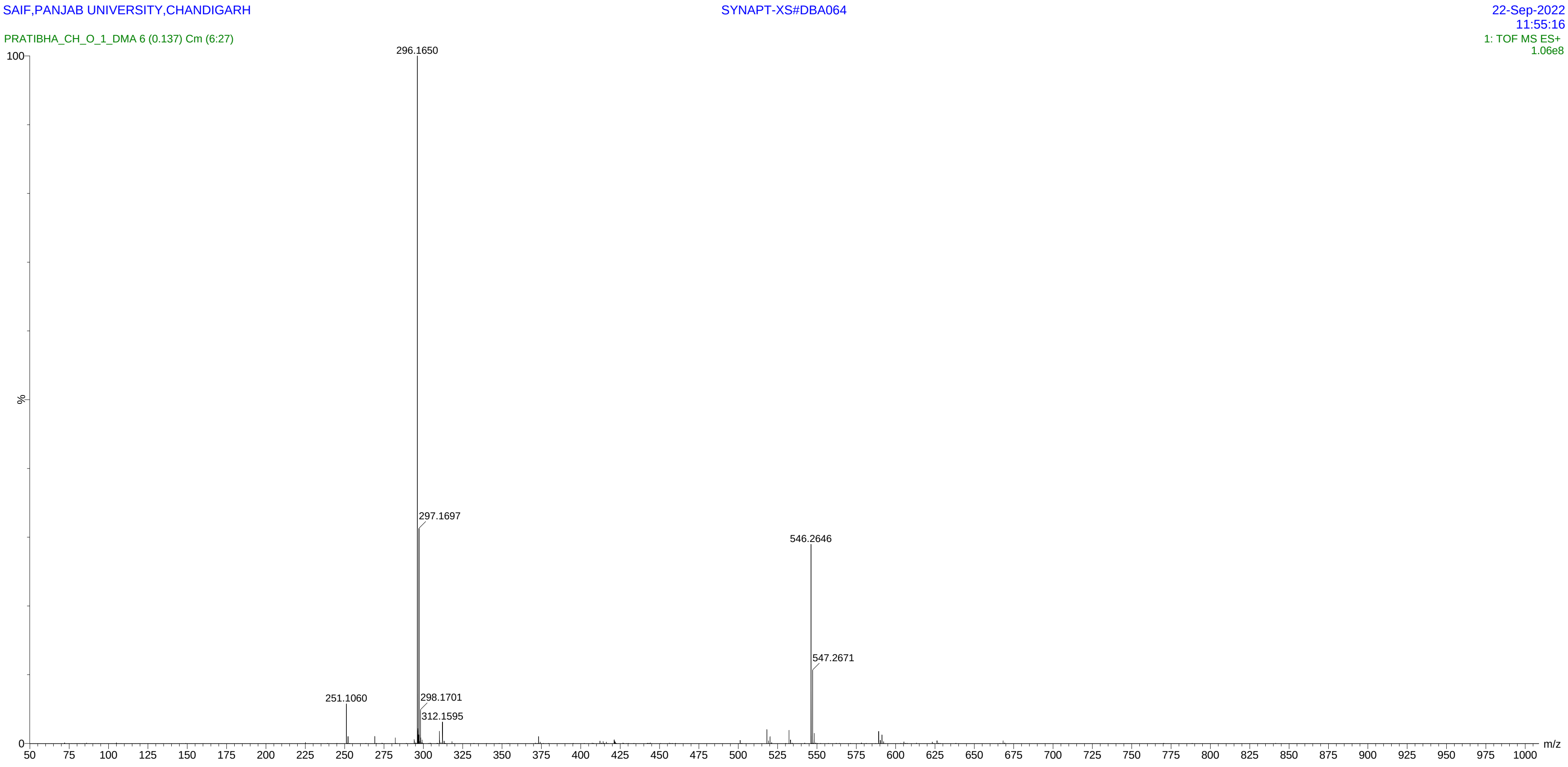

SAIF,PANJAB UNIVERSITY,CHANDIGARH
SYNAPT-XS#DBA064
22-Sep-2022
11:55:16
PRATIBHA_CH_O_1_DMA 6 (0.137) Cm (6:27)
1: TOF MS ES+
1.06e8
100
%
0
296.1650
297.1697
546.2646
547.2671
251.1060
298.1701
312.1595
m/z
50 75 100 125 150 175 200 225 250 275 300 325 350 375 400 425 450 475 500 525 550 575 600 625 650 675 700 725 750 775 800 825 850 875 900 925 950 975 1000

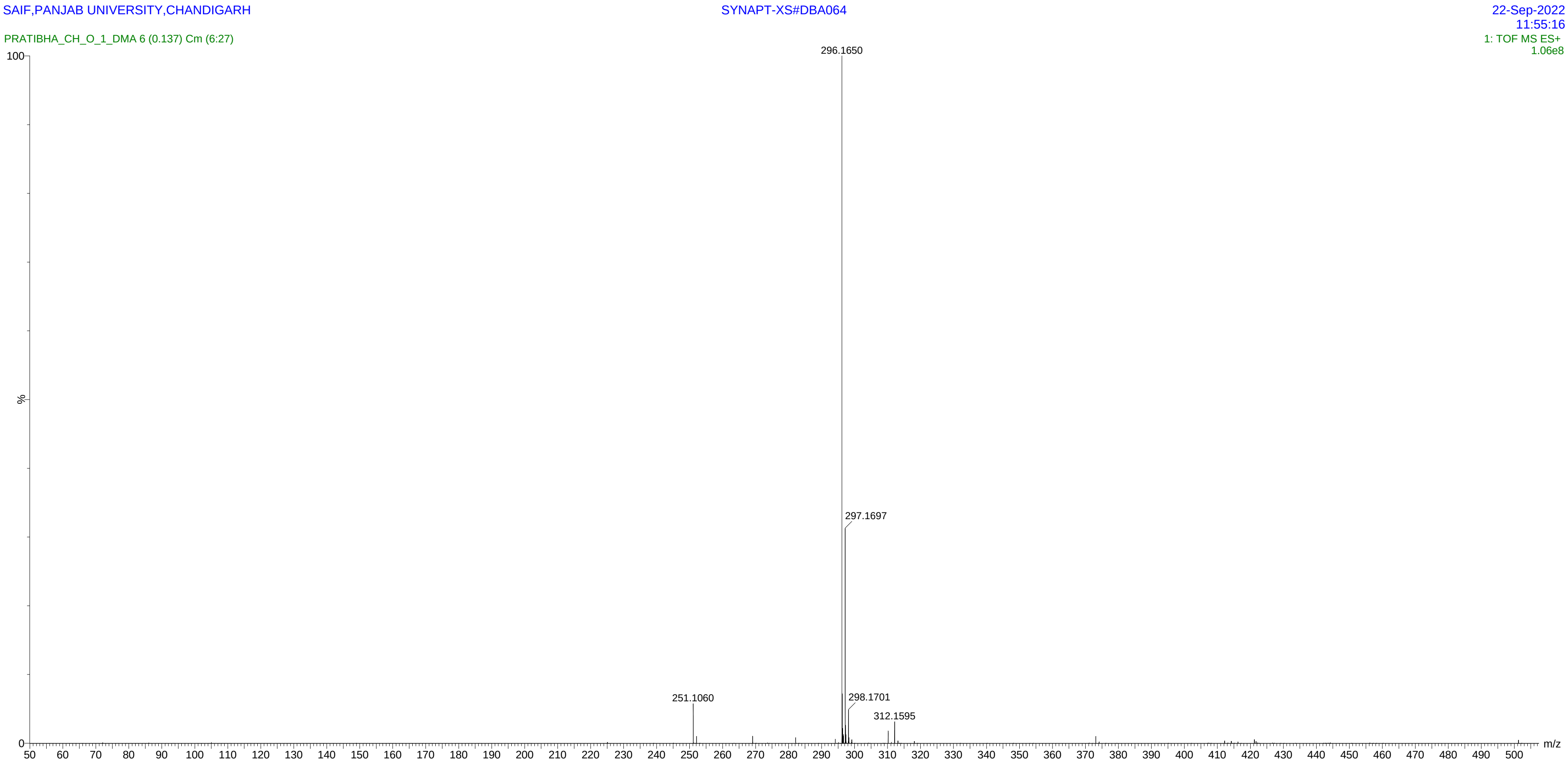
SAIF,PANJAB UNIVERSITY,CHANDIGARH
SYNAPT-XS#DBA064
22-Sep-2022
11:55:16
PRATIBHA_CH_O_1_DMA 6 (0.137) Cm (6:27)
1: TOF MS ES+
1.06e8
296.1650
297.1697
251.1060
298.1701
312.1595
%
m/z

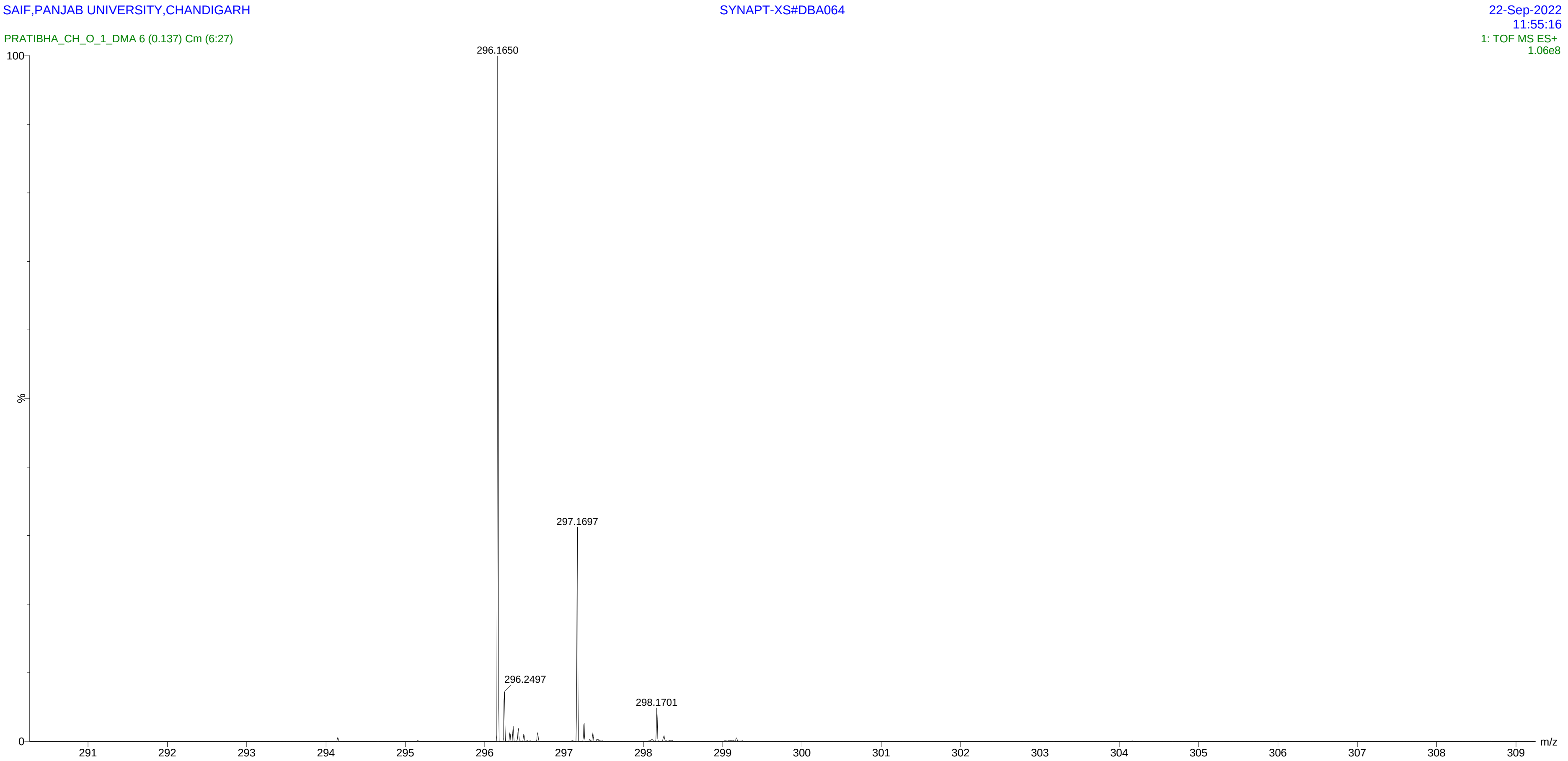
SAIF,PANJAB UNIVERSITY,CHANDIGARH
SYNAPT-XS#DBA064
22-Sep-2022
11:55:16
PRATIBHA_CH_O_1_DMA 6 (0.137) Cm (6:27)
1: TOF MS ES+
1.06e8
100
%
0
296.1650
297.1697
296.2497
298.1701
291
292
293
294
295
296
297
298
299
300
301
302
303
304
305
306
307
308
309
m/z

PS3

CH-O-4

1H_8scan CDCl3 {D:\Spectra} nmr 42

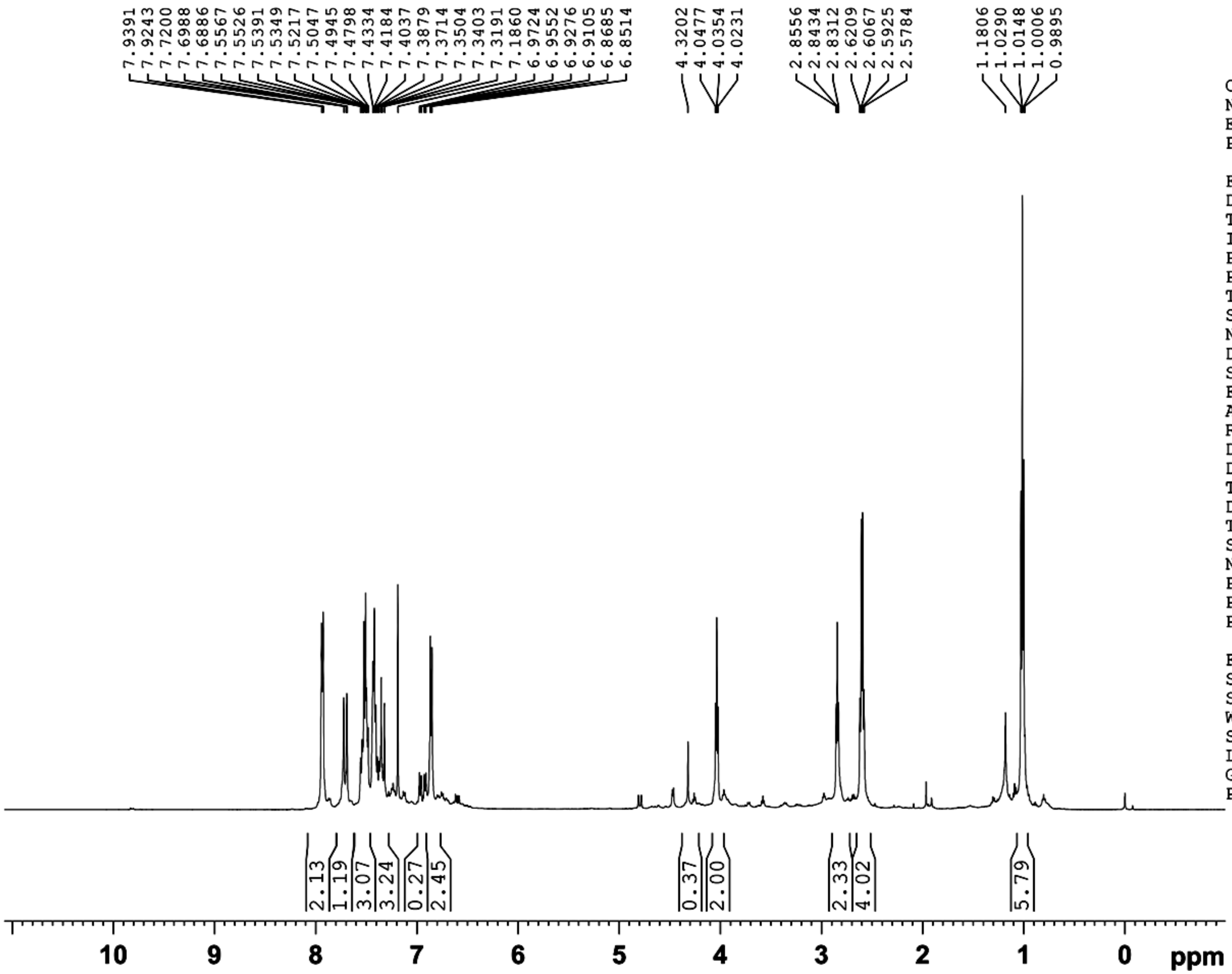

BRUKER
AVANCE NEO
500 MHz NMR
SPECTROMETER
SAIF, P.U.

Current Data Parameters

NAME	May05-2022
EXPNO	420
PROCNO	1

F2 - Acquisition Parameters

Date_	20220505
Time	15.58 h
INSTRUM	Avance Neo 500
PROBHD	Z119470_0333 (
PULPROG	zg30
TD	65536
SOLVENT	CDCl3
NS	16
DS	0
SWH	14705.883 Hz
FIDRES	0.448788 Hz
AQ	2.2282240 sec
RG	95.7854
DW	34.000 usec
DE	6.79 usec
TE	300.1 K
D1	1.00000000 sec
TD0	1
SFO1	500.1730885 MHz
NUC1	1H
P0	3.33 usec
P1	10.00 usec
PLW1	20.93000031 W

F2 - Processing parameters

SI	65536
SF	500.1700490 MHz
WDW	EM
SSB	0
LB	0.30 Hz
GB	0
PC	1.00

PS313
C13CPD CDCl3 {D:\Spectra} nmr 33

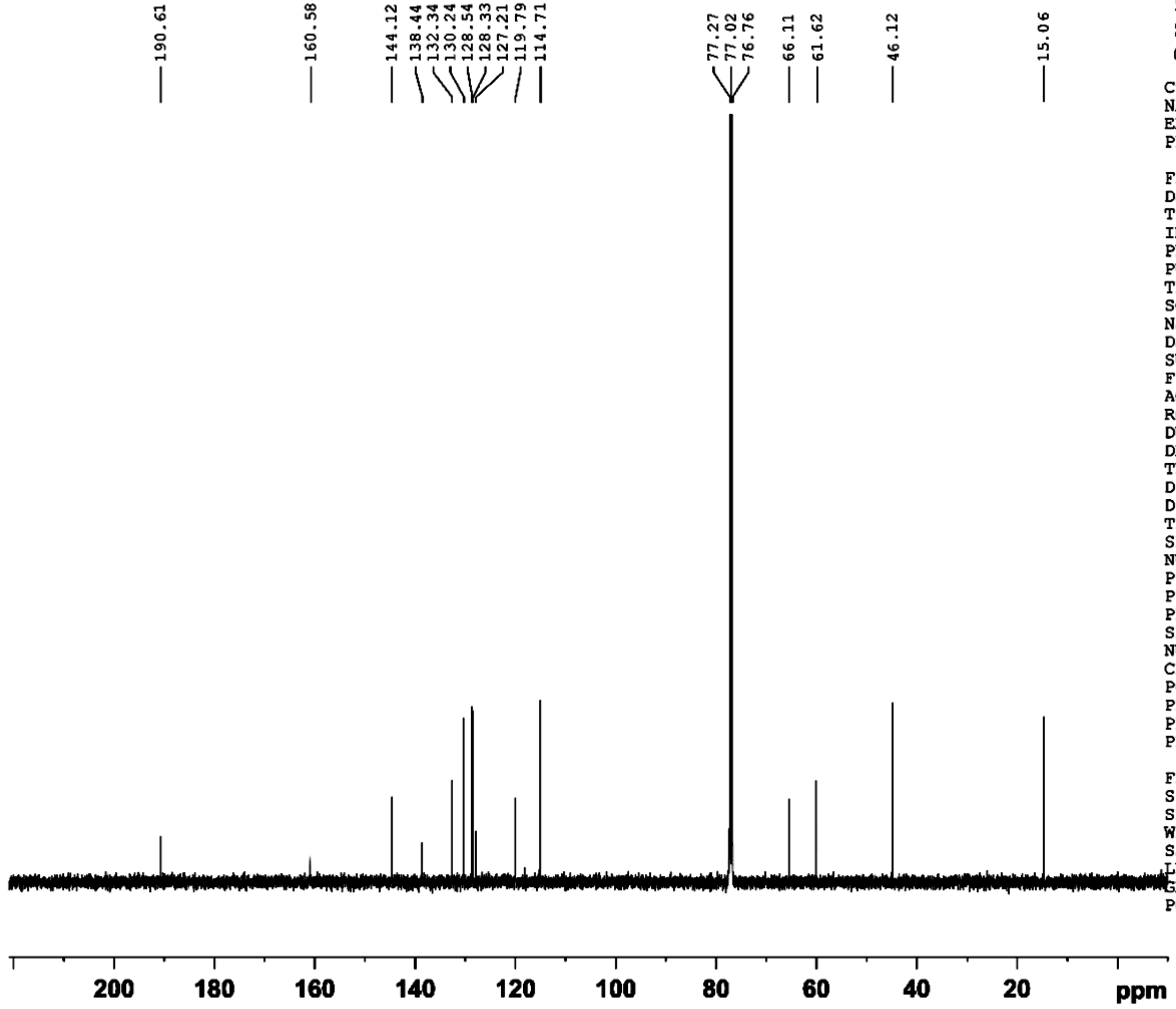

BRUKER
AVANCE NEO
500 MHz NMR SPECTROMETER
SAIF, PANJAB UNIVERSITY,
CHANDIGARH

Current Data Parameters
NAME Sep15-2022
EXPNO 331
PROCNO 1

F2 - Acquisition Parameters
Date_ 20220915
Time 21.54 h
INSTRUM Avance Neo 500
PROBHD Z119470_0333 (
PULPROG zgpg30
TD 65536
SOLVENT CDCl3
NS 512
DS 4
SWH 37037.035 Hz
FIDRES 1.130281 Hz
AQ 0.8847360 sec
RG 101
DW 13.500 usec
DE 6.50 usec
TE 300.1 K
D1 2.00000000 sec
D11 0.03000000 sec
TD0 1
SFO1 125.7804233 MHz
NUC1 13C
P0 3.33 usec
P1 10.00 usec
PLW1 83.14099884 W
SFO2 500.1720007 MHz
NUC2 1H
CPDPRG[2 waltz65
PCPD2 80.00 usec
PLW2 20.93000031 W
PLW12 0.32703000 W
PLW13 0.16449000 W

F2 - Processing parameters
SI 32768
SF 125.7678465 MHz
WDW EM
SSB 0
LB 1.00 Hz
GB 0
PC 1.40

SAIF,PANJAB UNIVERSITY,CHANDIGARH

SYNAPT-XS#DBA064

22-Sep-2022
11:58:10

PRATIBHA_PS_3 5 (0.120) Cm (4:9)

1: TOF MS ES+
3.19e7

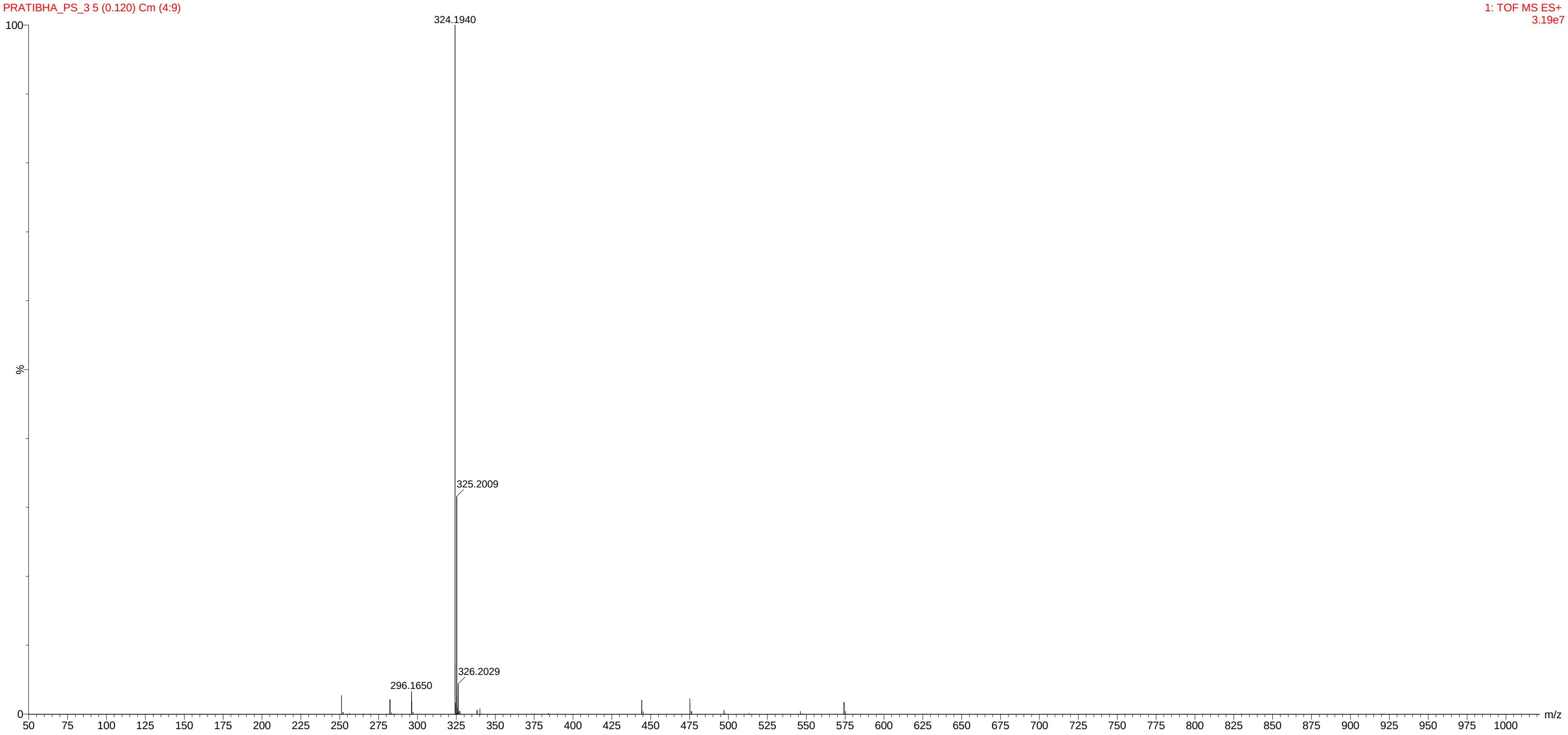

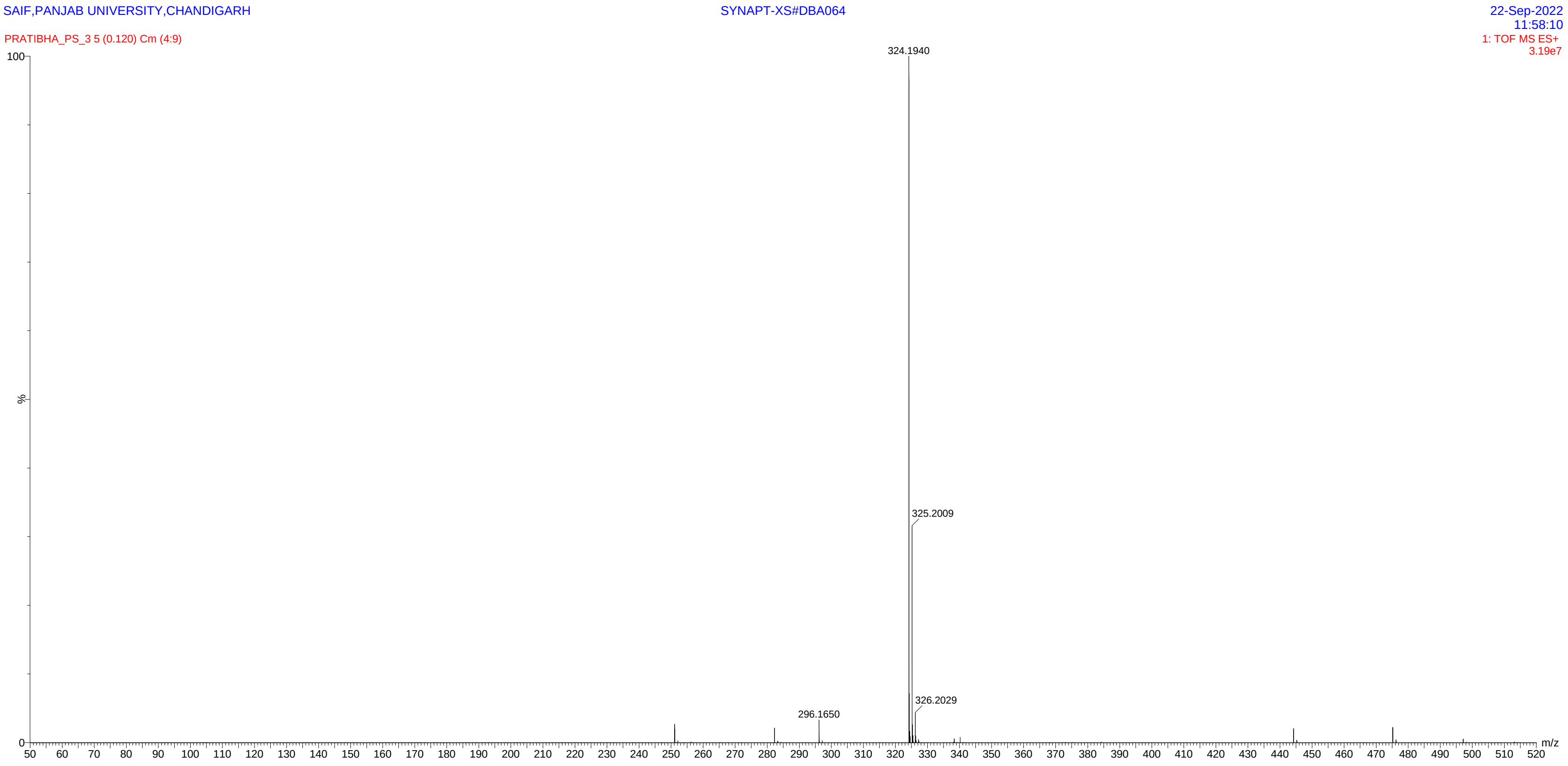
SAIF,PANJAB UNIVERSITY,CHANDIGARH
SYNAPT-XS#DBA064
22-Sep-2022
11:58:10
PRATIBHA_PS_3 5 (0.120) Cm (4:9)
1: TOF MS ES+
3.19e7
324.1940
325.2009
326.2029
296.1650
%
100
0
m/z

PS4

CH-O-3
1H_8scan CDCl3 {D:\Spectra} nmr 25

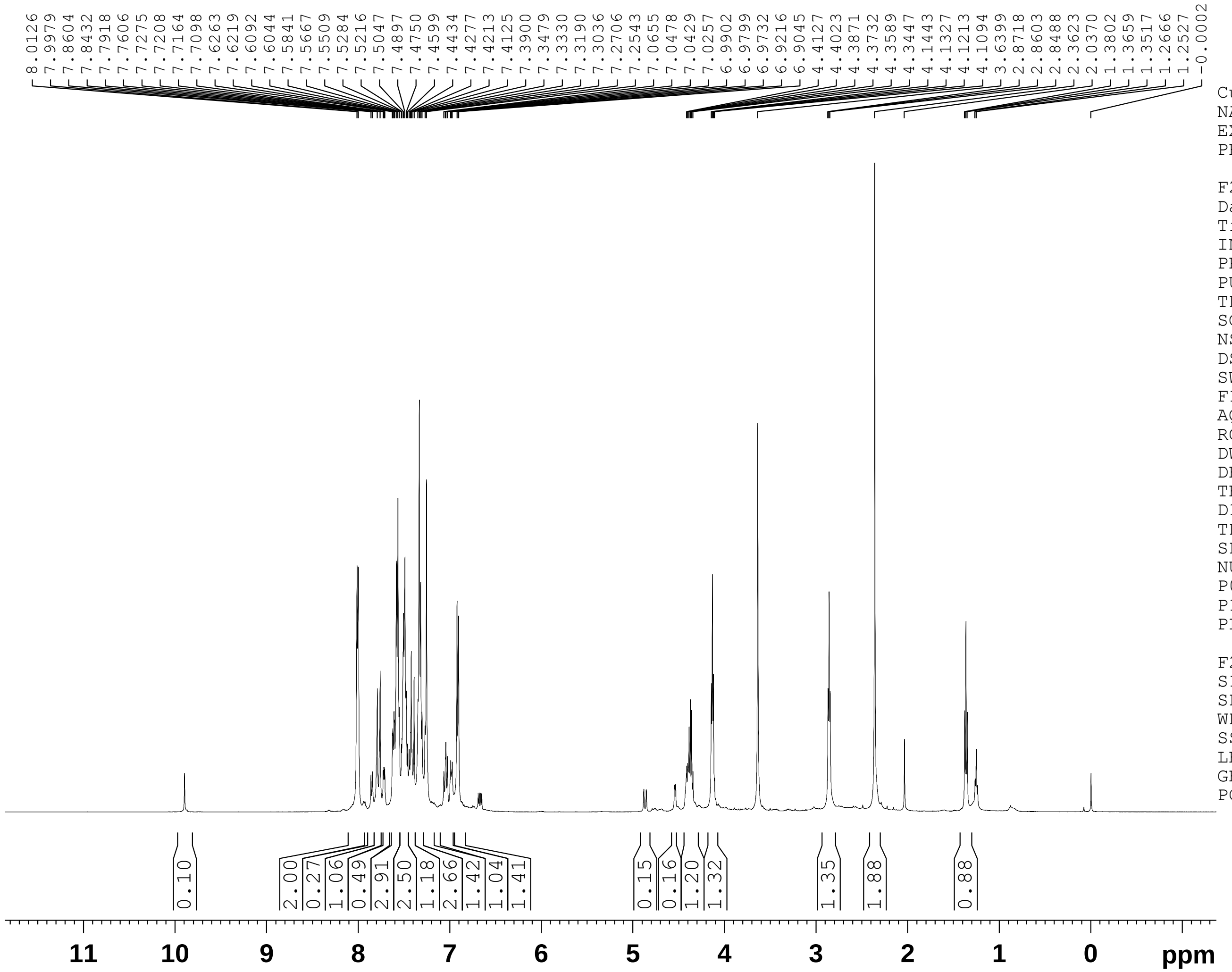

BRUKER
AVANCE NEO
500 MHz NMR
SPECTROMETER
SAIF, P.U.

Current Data Parameters
NAME Mar31-2022
EXPNO 250
PROCNO 1

F2 - Acquisition Parameters
Date_ 20220331
Time 16.44 h
INSTRUM Avance Neo 500
PROBHD Z119470_0333 (
PULPROG zg30
TD 65536
SOLVENT CDCl3
NS 16
DS 0
SWH 14705.883 Hz
FIDRES 0.448788 Hz
AQ 2.2282240 sec
RG 95.7854
DW 34.000 usec
DE 6.79 usec
TE 300.1 K
D1 1.00000000 sec
TD0 1
SFO1 500.1730885 MHz
NUC1 1H
P0 3.33 usec
P1 10.00 usec
PLW1 20.93000031 W

F2 - Processing parameters
SI 65536
SF 500.1700148 MHz
WDW EM
SSB 0
LB 0.30 Hz
GB 0
PC 1.00

CH-O-3
1H_8scan CDCl3 {D:\Spectra} nmr 25

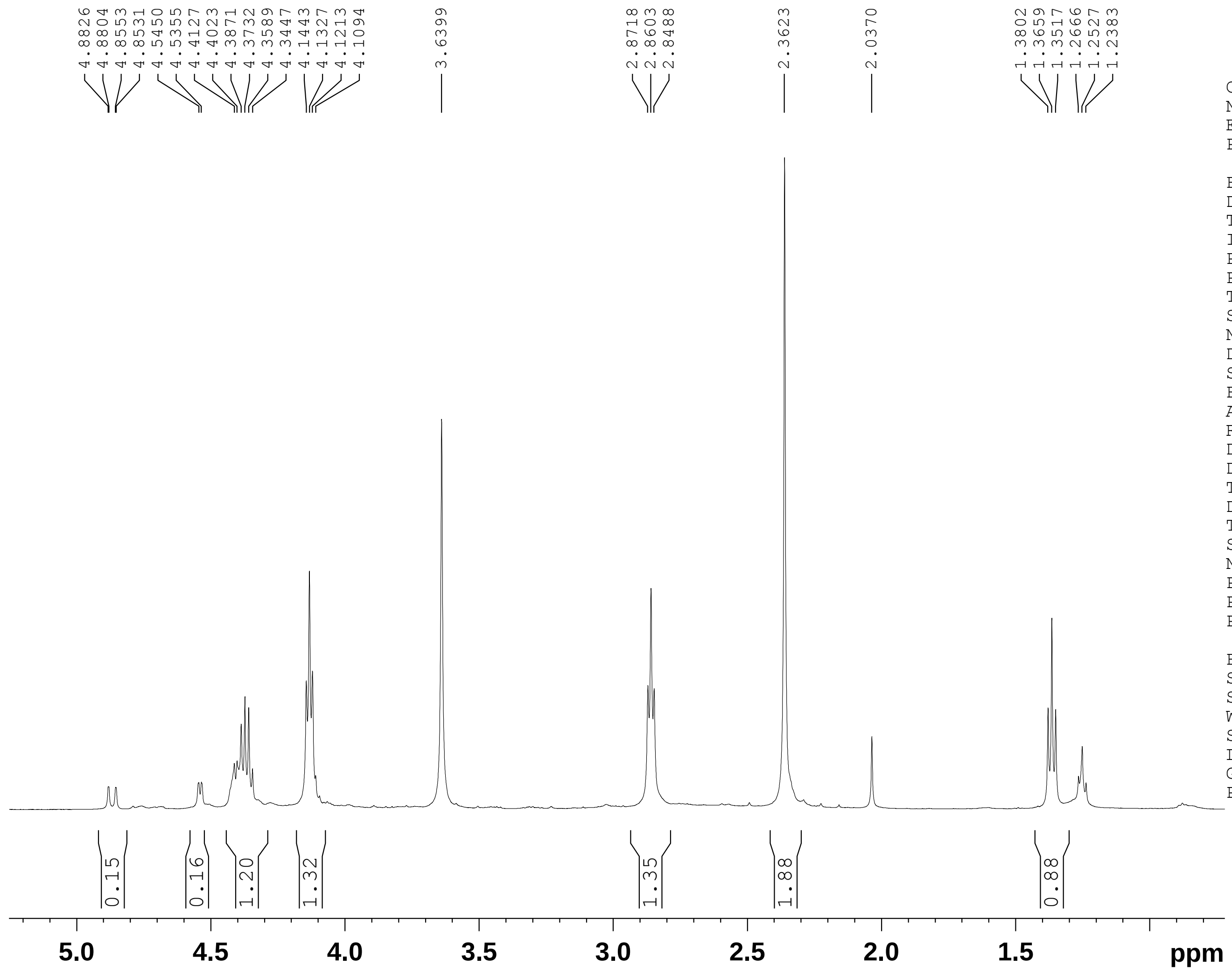

BRUKER
AVANCE NEO
500 MHz NMR
SPECTROMETER
SAIF, P.U.

Current Data Parameters

NAME	Mar31-2022
EXPNO	250
PROCNO	1

F2 - Acquisition Parameters

Date_	20220331
Time	16.44 h
INSTRUM	Avance Neo 500
PROBHD	Z119470_0333 (
PULPROG	zg30
TD	65536
SOLVENT	CDCl3
NS	16
DS	0
SWH	14705.883 Hz
FIDRES	0.448788 Hz
AQ	2.2282240 sec
RG	95.7854
DW	34.000 usec
DE	6.79 usec
TE	300.1 K
D1	1.00000000 sec
TD0	1
SFO1	500.1730885 MHz
NUC1	1H
P0	3.33 usec
P1	10.00 usec
PLW1	20.93000031 W

F2 - Processing parameters

SI	65536
SF	500.1700148 MHz
WDW	EM
SSB	0
LB	0.30 Hz
GB	0
PC	1.00

CH-O-3
C13CPD CDCl3 {D:\Spectra} nmr 25

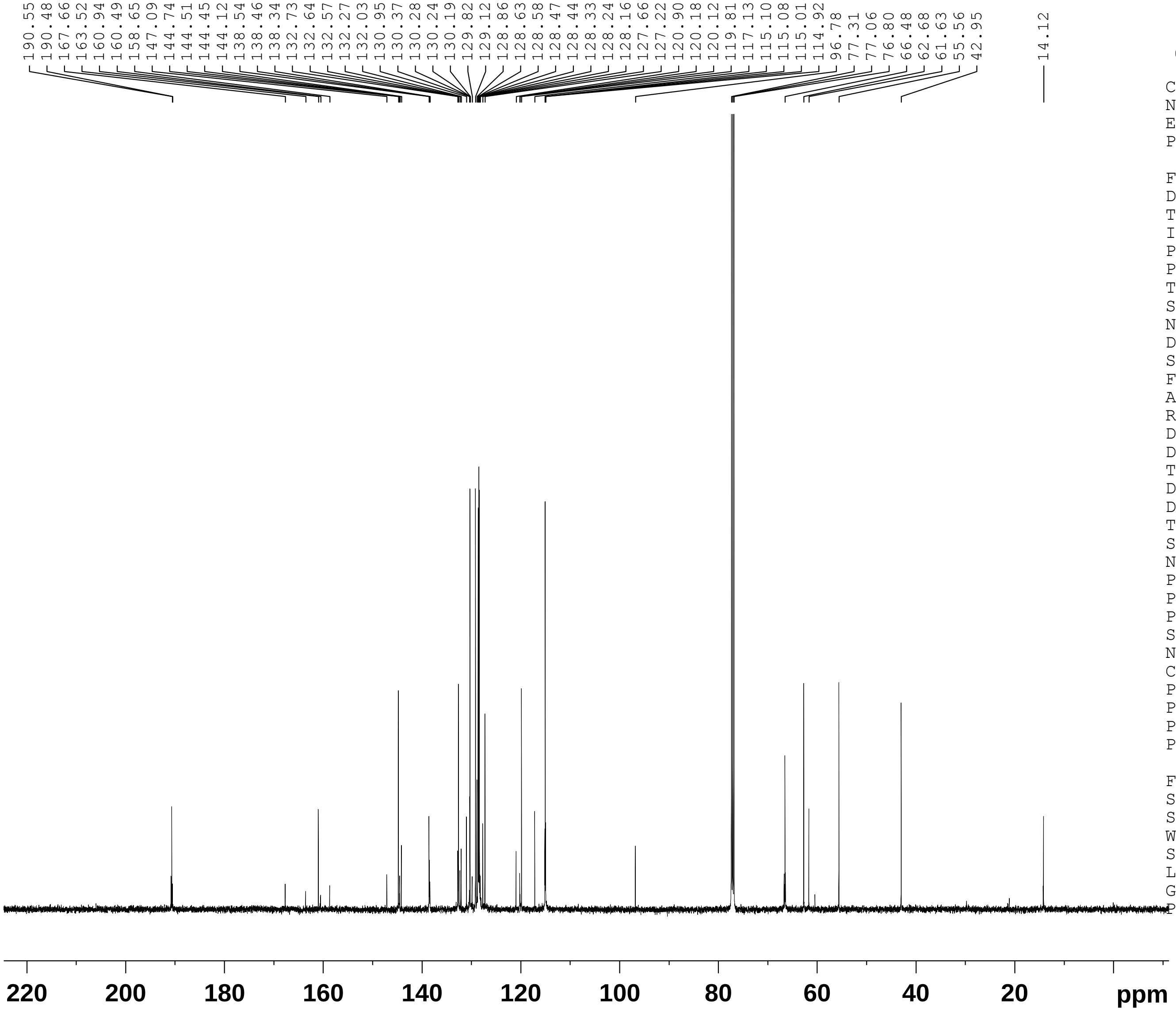

BRUKER
AVANCE NEO
500 MHz NMR SPECTROMETER
SAIF, PANJAB UNIVERSITY,
CHANDIGARH

Current Data Parameters
NAME Mar31-2022
EXPNO 251
PROCNO 1

F2 - Acquisition Parameters
Date_ 20220331
Time 17.35 h
INSTRUM Avance Neo 500
PROBHD Z119470_0333 (
PULPROG zgpg30
TD 65536
SOLVENT CDCl3
NS 1024
DS 4
SWH 37037.035 Hz
FIDRES 1.130281 Hz
AQ 0.8847360 sec
RG 101
DW 13.500 usec
DE 6.50 usec
TE 300.2 K
D1 2.00000000 sec
D11 0.03000000 sec
TD0 1
SFO1 125.7804233 MHz
NUC1 13C
P0 3.33 usec
P1 10.00 usec
PLW1 83.14099884 W
SFO2 500.1720007 MHz
NUC2 1H
CPDPRG[2 waltz65
PCPD2 80.00 usec
PLW2 20.93000031 W
PLW12 0.32703000 W
PLW13 0.16449000 W

F2 - Processing parameters
SI 32768
SF 125.7678465 MHz
WDW EM
SSB 0
LB 1.00 Hz
GB 0
PC 1.40

SAIF,PANJAB UNIVERSITY,CHANDIGARH

SYNAPT-XS#DBA064

22-Sep-2022
12:24:02

PR5 (0.120) Cm (4:9)

1: TOF MS ES+
1.14e7

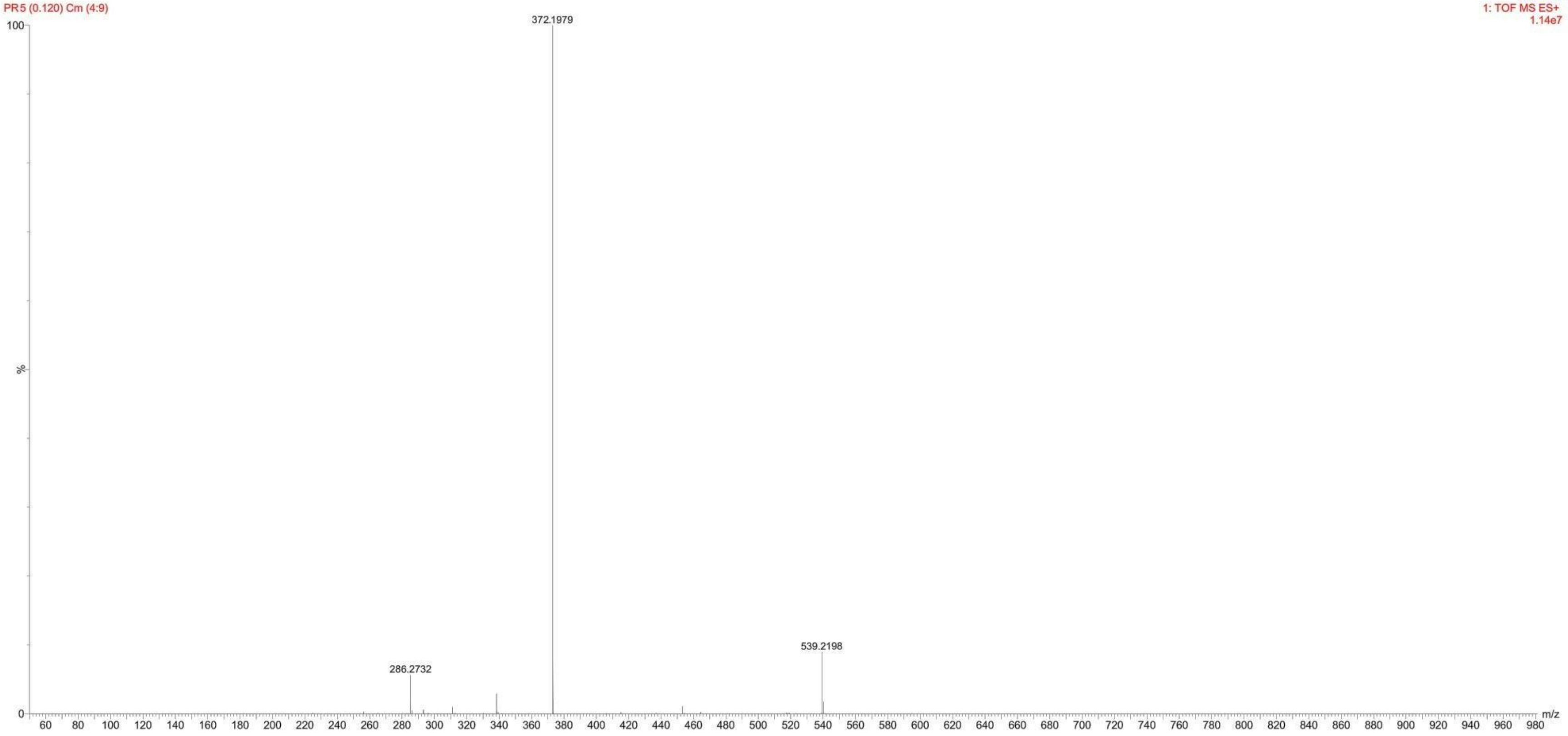

PS5

CH-O-5

1H_8scan CDCl3 {D:\Spectra} nmr 26

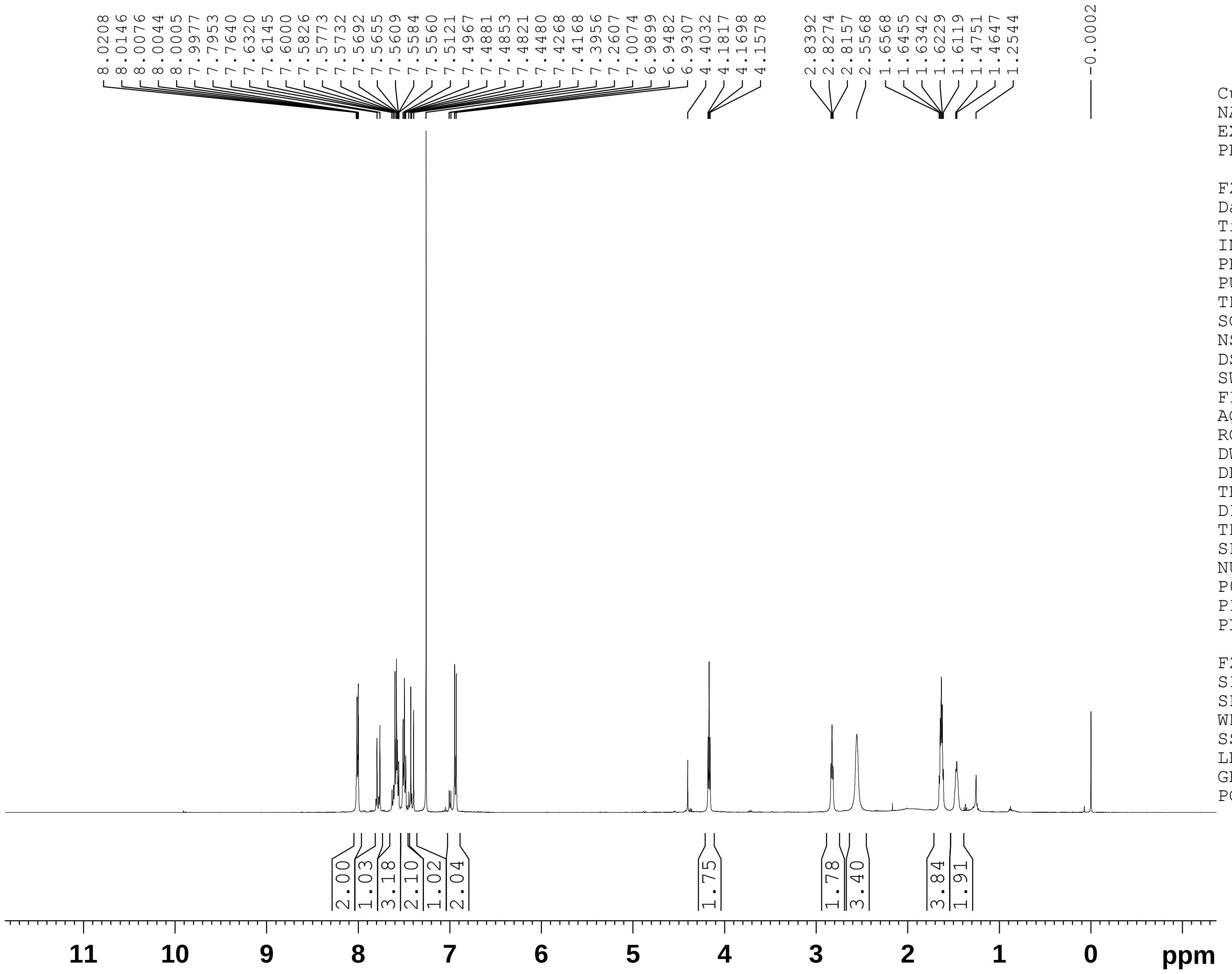

BRUKER
AVANCE NEO
500 MHz NMR
SPECTROMETER
SAIF, P.U.

Current Data Parameters

NAME	Mar31-2022
EXPNO	260
PROCNO	1

F2 - Acquisition Parameters

Date_	20220331
Time	17.38 h
INSTRUM	Avance Neo 500
PROBHD	Z119470_0333 (
PULPROG	zg30
TD	65536
SOLVENT	CDCl3
NS	16
DS	0
SWH	14705.883 Hz
FIDRES	0.448788 Hz
AQ	2.2282240 sec
RG	101
DW	34.000 usec
DE	6.79 usec
TE	300.2 K
D1	1.00000000 sec
TD0	1
SFO1	500.1730885 MHz
NUC1	1H
P0	3.33 usec
P1	10.00 usec
PLW1	20.93000031 W

F2 - Processing parameters

SI	65536
SF	500.1700117 MHz
WDW	EM
SSB	0
LB	0.30 Hz
GB	0
PC	1.00

CH-O-5
1H_8scan CDCl3 {D:\Spectra} nmr 26

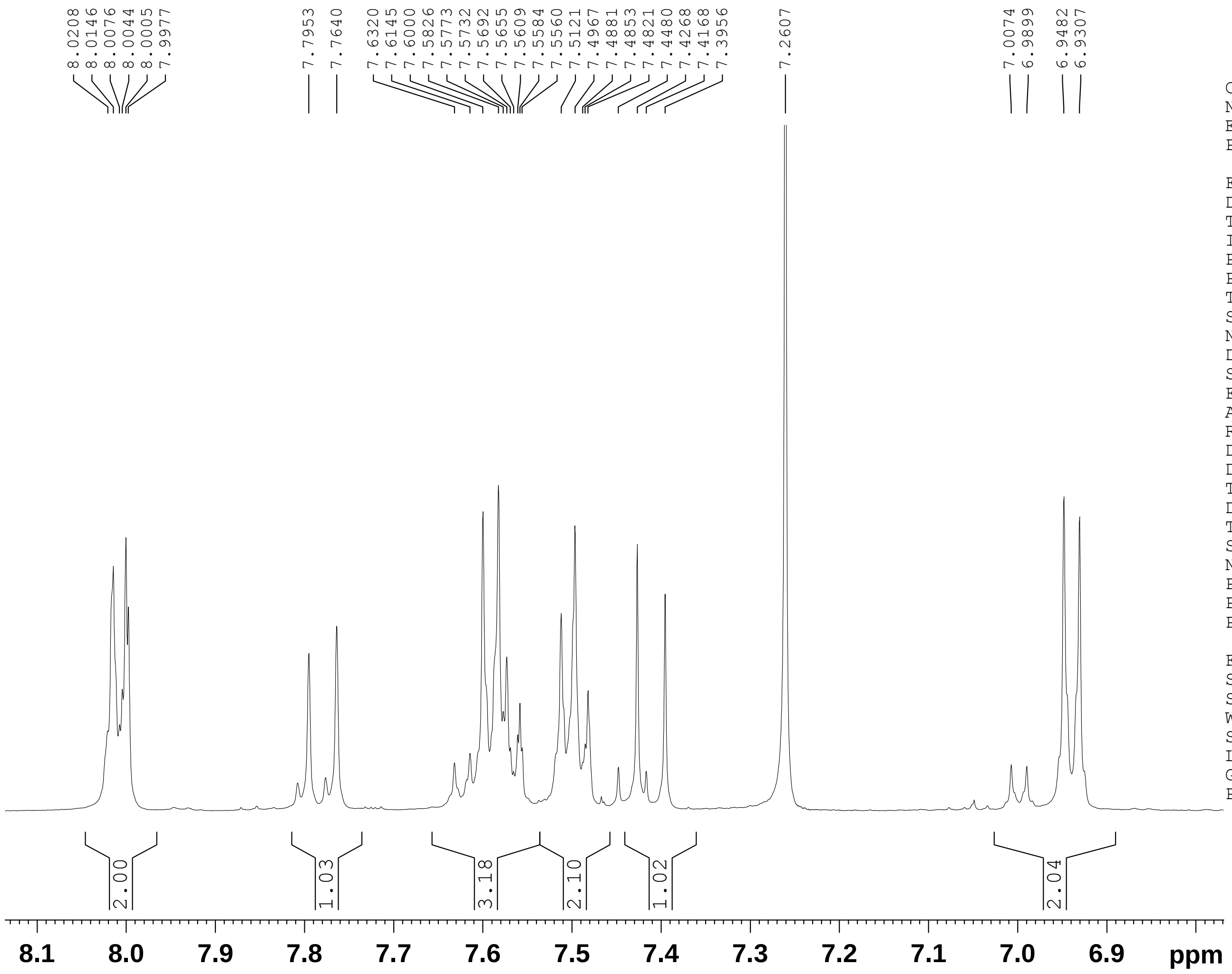

BRUKER
AVANCE NEO
500 MHz NMR
SPECTROMETER
SAIF, P.U.

Current Data Parameters
NAME Mar31-2022
EXPNO 260
PROCNO 1

F2 - Acquisition Parameters
Date_ 20220331
Time 17.38 h
INSTRUM Avance Neo 500
PROBHD Z119470_0333 (
PULPROG zg30
TD 65536
SOLVENT CDCl3
NS 16
DS 0
SWH 14705.883 Hz
FIDRES 0.448788 Hz
AQ 2.2282240 sec
RG 101
DW 34.000 usec
DE 6.79 usec
TE 300.2 K
D1 1.00000000 sec
TD0 1
SFO1 500.1730885 MHz
NUC1 1H
P0 3.33 usec
P1 10.00 usec
PLW1 20.93000031 W

F2 - Processing parameters
SI 65536
SF 500.1700117 MHz
WDW EM
SSB 0
LB 0.30 Hz
GB 0
PC 1.00

CH-O-5
1H_8scan CDCl3 {D:\Spectra} nmr 26

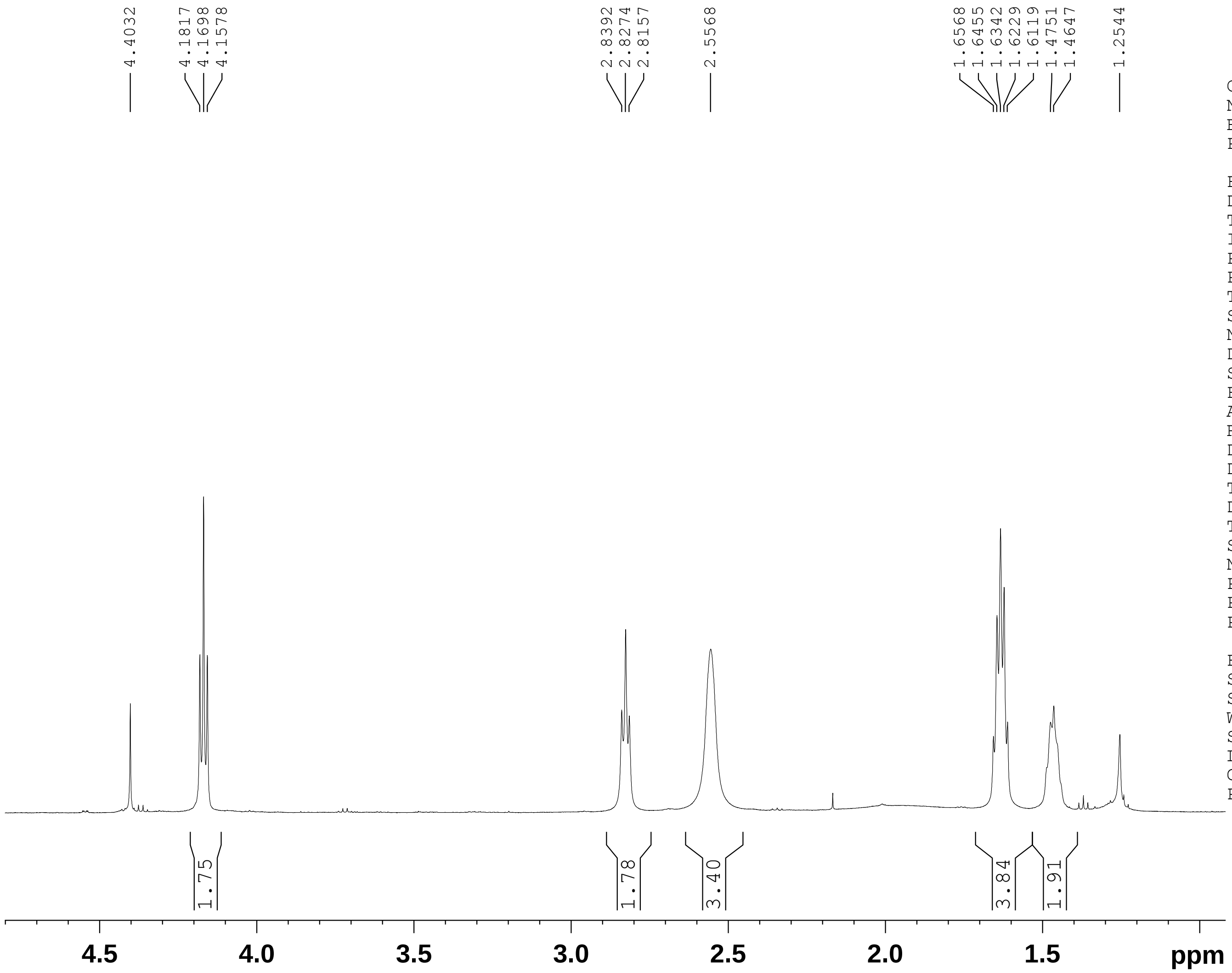

BRUKER
AVANCE NEO
500 MHz NMR
SPECTROMETER
SAIF, P.U.

Current Data Parameters
NAME Mar31-2022
EXPNO 260
PROCNO 1

F2 - Acquisition Parameters
Date_ 20220331
Time 17.38 h
INSTRUM Avance Neo 500
PROBHD Z119470_0333 (
PULPROG zg30
TD 65536
SOLVENT CDCl3
NS 16
DS 0
SWH 14705.883 Hz
FIDRES 0.448788 Hz
AQ 2.2282240 sec
RG 101
DW 34.000 usec
DE 6.79 usec
TE 300.2 K
D1 1.00000000 sec
TD0 1
SFO1 500.1730885 MHz
NUC1 1H
P0 3.33 usec
P1 10.00 usec
PLW1 20.93000031 W

F2 - Processing parameters
SI 65536
SF 500.1700117 MHz
WDW EM
SSB 0
LB 0.30 Hz
GB 0
PC 1.00

CH-O-5
C13CPD CDCl3 {D:\Spectra} nmr 26

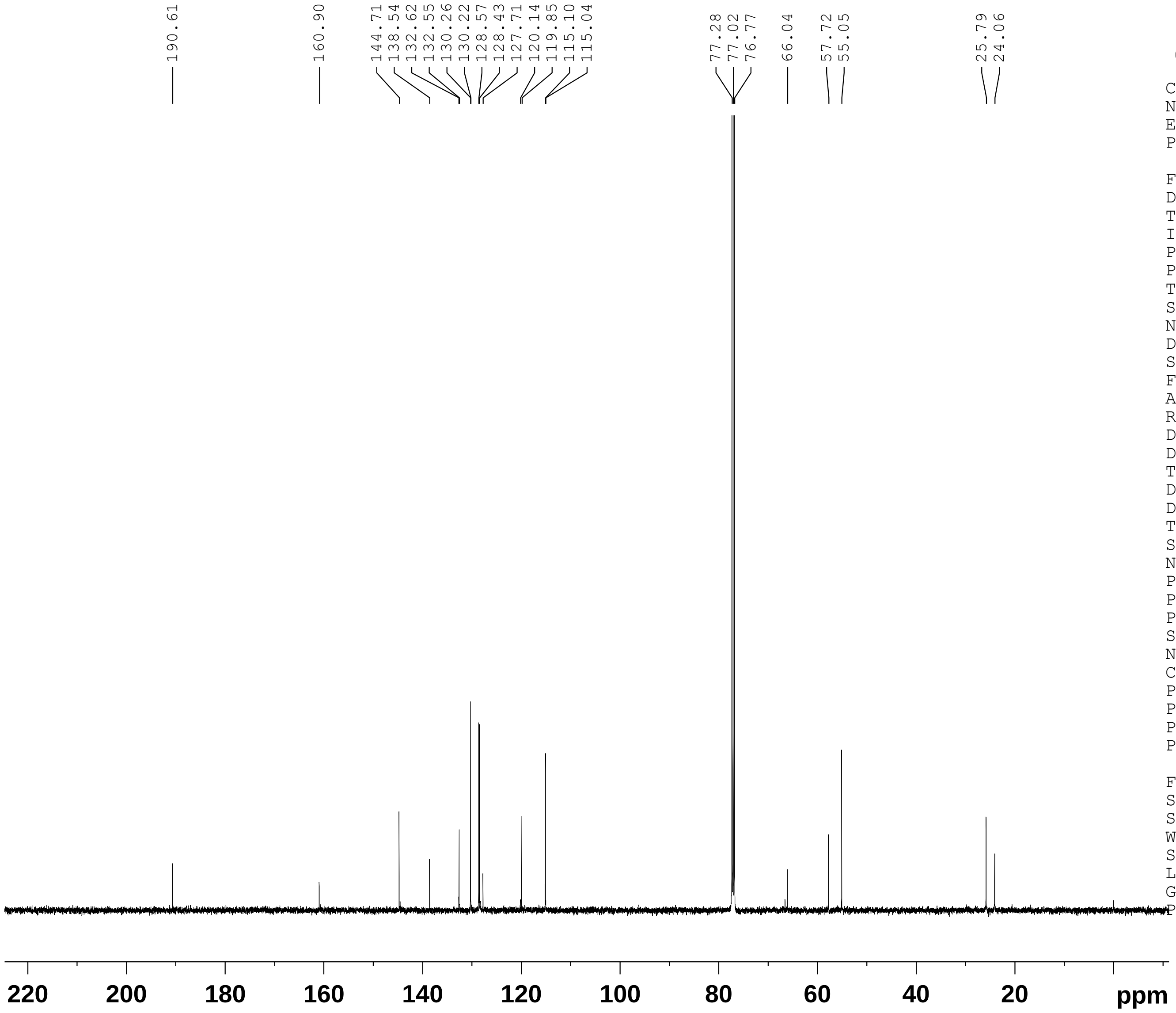

BRUKER
AVANCE NEO
500 MHz NMR SPECTROMETER
SAIF, PANJAB UNIVERSITY,
CHANDIGARH

Current Data Parameters

NAME	Mar31-2022	
EXPNO	261	
PROCNO	1	

F2 - Acquisition Parameters

Date_	20220331	
Time	18.29	h
INSTRUM	Avance Neo 500	
PROBHD	Z119470_0333 (	
PULPROG	zgpg30	
TD	65536	
SOLVENT	CDCl3	
NS	1024	
DS	4	
SWH	37037.035	Hz
FIDRES	1.130281	Hz
AQ	0.8847360	sec
RG	101	
DW	13.500	usec
DE	6.50	usec
TE	300.2	K
D1	2.00000000	sec
D11	0.03000000	sec
TD0	1	
SFO1	125.7804233	MHz
NUC1	13C	
P0	3.33	usec
P1	10.00	usec
PLW1	83.14099884	W
SFO2	500.1720007	MHz
NUC2	1H	
CPDPRG[2	waltz65	
PCPD2	80.00	usec
PLW2	20.93000031	W
PLW12	0.32703000	W
PLW13	0.16449000	W

F2 - Processing parameters

SI	32768	
SF	125.7678465	MHz
WDW	EM	
SSB	0	
LB	1.00	Hz
GB	0	
PC	1.40	

CH-O-5
C13CPD CDCl3 {D:\Spectra} nmr 26

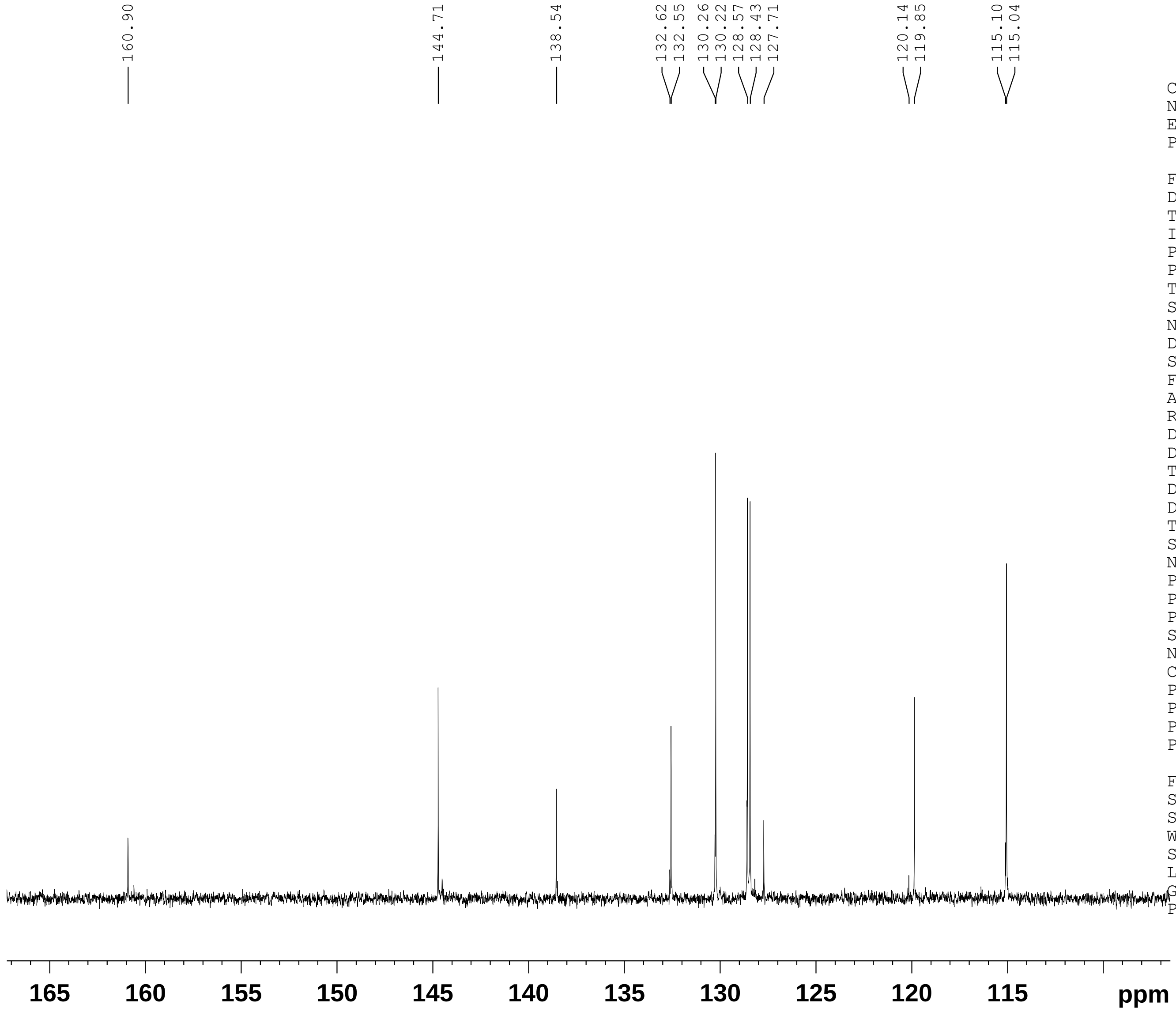

BRUKER
AVANCE NEO
500 MHz NMR SPECTROMETER
SAIF, PANJAB UNIVERSITY,
CHANDIGARH

Current Data Parameters

NAME	Mar31-2022	
EXPNO	261	
PROCNO	1	

F2 - Acquisition Parameters

Date_	20220331	
Time	18.29	h
INSTRUM	Avance Neo 500	
PROBHD	Z119470_0333 (	
PULPROG	zgpg30	
TD	65536	
SOLVENT	CDCl3	
NS	1024	
DS	4	
SWH	37037.035	Hz
FIDRES	1.130281	Hz
AQ	0.8847360	sec
RG	101	
DW	13.500	usec
DE	6.50	usec
TE	300.2	K
D1	2.00000000	sec
D11	0.03000000	sec
TD0	1	
SFO1	125.7804233	MHz
NUC1	13C	
P0	3.33	usec
P1	10.00	usec
PLW1	83.14099884	W
SFO2	500.1720007	MHz
NUC2	1H	
CPDPRG[2	waltz65	
PCPD2	80.00	usec
PLW2	20.93000031	W
PLW12	0.32703000	W
PLW13	0.16449000	W

F2 - Processing parameters

SI	32768	
SF	125.7678465	MHz
WDW	EM	
SSB	0	
LB	1.00	Hz
GB	0	
PC	1.40	

PS6

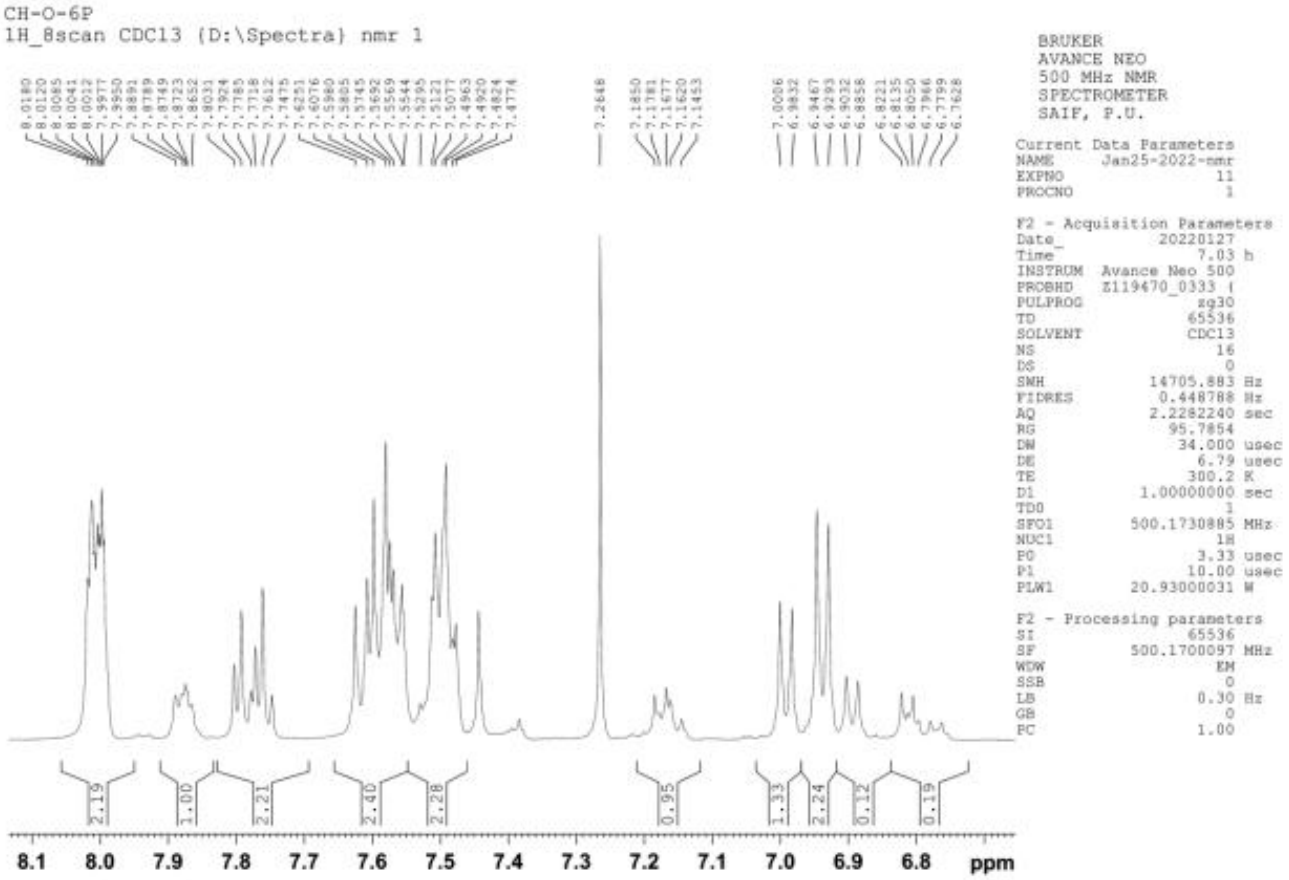

CH-O-6P
1H_8scan CDCl3 (D:\Spectra) nmr 1
BRUKER
AVANCE NEO
500 MHz NMR
SPECTROMETER
SAIF, P.U.
Current Data Parameters
NAME Jan25-2022-nmr
EXPNO 11
PROCNO 1
F2 - Acquisition Parameters
Date_ 20220127
Time 7.03 h
INSTRUM Avance Neo 500
PROBHD Z119470_0333 (
PULPROG zg30
TD 65536
SOLVENT CDCl3
NS 16
DS 0
SWH 14705.883 Hz
FIDRES 0.448788 Hz
AQ 2.2282240 sec
RG 95.7854
DW 34.000 usec
DE 6.79 usec
TE 300.2 K
D1 1.00000000 sec
TD0 1
SFO1 500.1730885 MHz
NUC1 1H
P0 3.33 usec
P1 10.00 usec
PLW1 20.93000031 W
F2 - Processing parameters
SI 65536
SF 500.1700097 MHz
WDW EM
SSB 0
LB 0.30 Hz
GB 0
PC 1.00
2.19
1.00
2.21
2.40
2.28
0.95
1.33
2.24
0.12
0.19
8.1
8.0
7.9
7.8
7.7
7.6
7.5
7.4
7.3
7.2
7.1
7.0
6.9
6.8
ppm

CH-O-6P
1H_8scan CDCl3 (D:\Spectra) nmr 1

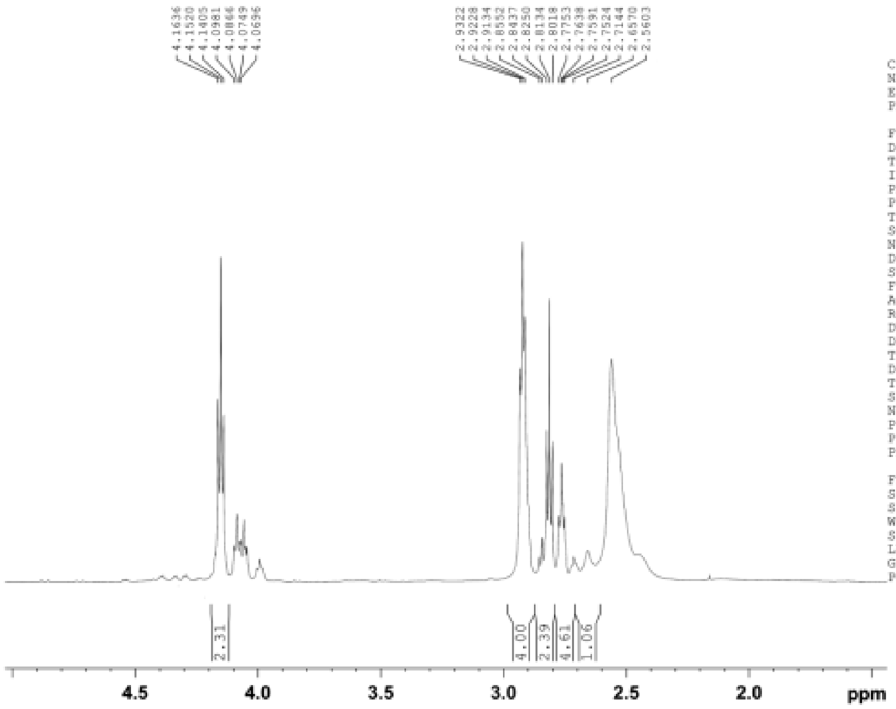

BRUKER
AVANCE NEO
500 MHz NMR
SPECTROMETER
SAIF, P.U.

Current Data Parameters

NAME	Jan25-2022-nmr
EXPNO	11
PROCNO	1

F2 - Acquisition Parameters

Date_	20220127
Time	7.03 h
INSTRUM	Avance Neo 500
PROBHD	Z119470_0333 (
PULPROG	zg30
TD	65536
SOLVENT	CDCl3
NS	16
DS	0
SWH	14705.883 Hz
FIDRES	0.448788 Hz
AQ	2.2282240 sec
RG	95.7854
DW	34.000 usec
DE	6.79 usec
TE	300.2 K
D1	1.00000000 sec
TD0	1
SFO1	500.1730885 MHz
NUC1	1H
P0	3.33 usec
P1	10.00 usec
PLW1	20.93000031 W

F2 - Processing parameters

SI	65536
SF	500.1700097 MHz
WDW	EM
SSB	0
LB	0.30 Hz
GB	0
PC	1.00

CH-O-6P
C13CPD CDCl3 {D:\Spectra} nmr 1

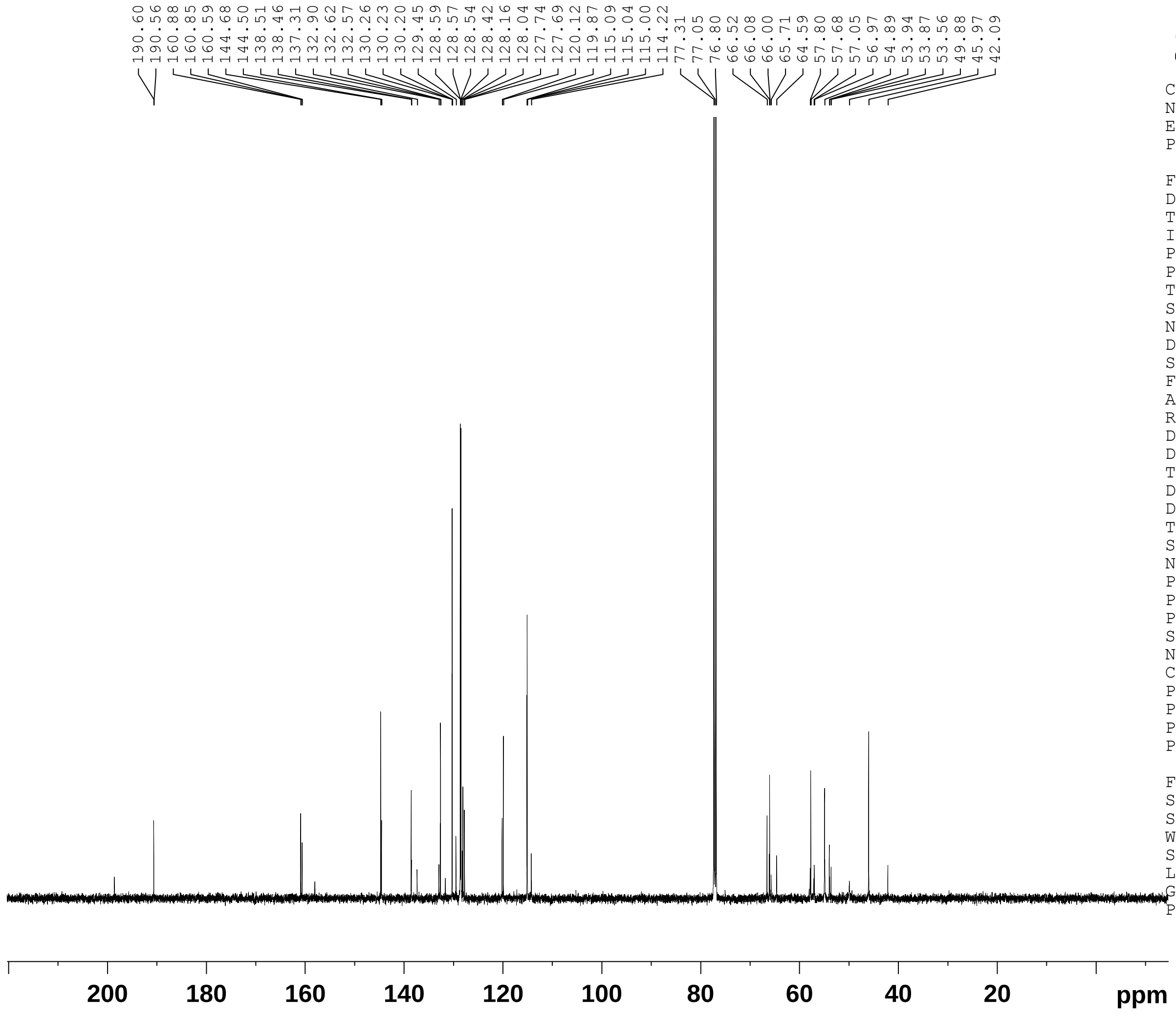

BRUKER
AVANCE NEO
500 MHz NMR SPECTROMETER
SAIF, PANJAB UNIVERSITY,
CHANDIGARH

Current Data Parameters

NAME	Jan25-2022-nmr	
EXPNO	12	
PROCNO	1	

F2 - Acquisition Parameters

Date_	20220127	
Time	7.29	h
INSTRUM	Avance Neo 500	
PROBHD	Z119470_0333 (	
PULPROG	zgpg30	
TD	65536	
SOLVENT	CDCl3	
NS	512	
DS	4	
SWH	37037.035	Hz
FIDRES	1.130281	Hz
AQ	0.8847360	sec
RG	101	
DW	13.500	usec
DE	6.50	usec
TE	300.1	K
D1	2.00000000	sec
D11	0.03000000	sec
TD0	1	
SFO1	125.7804233	MHz
NUC1	13C	
P0	3.33	usec
P1	10.00	usec
PLW1	83.14099884	W
SFO2	500.1720007	MHz
NUC2	1H	
CPDPRG[2	waltz65	
PCPD2	80.00	usec
PLW2	20.93000031	W
PLW12	0.32703000	W
PLW13	0.16449000	W

F2 - Processing parameters

SI	32768	
SF	125.7678465	MHz
WDW	EM	
SSB	0	
LB	1.00	Hz
GB	0	
PC	1.40	

CH-O-6P
C13CPD CDCl3 {D:\Spectra} nmr 1

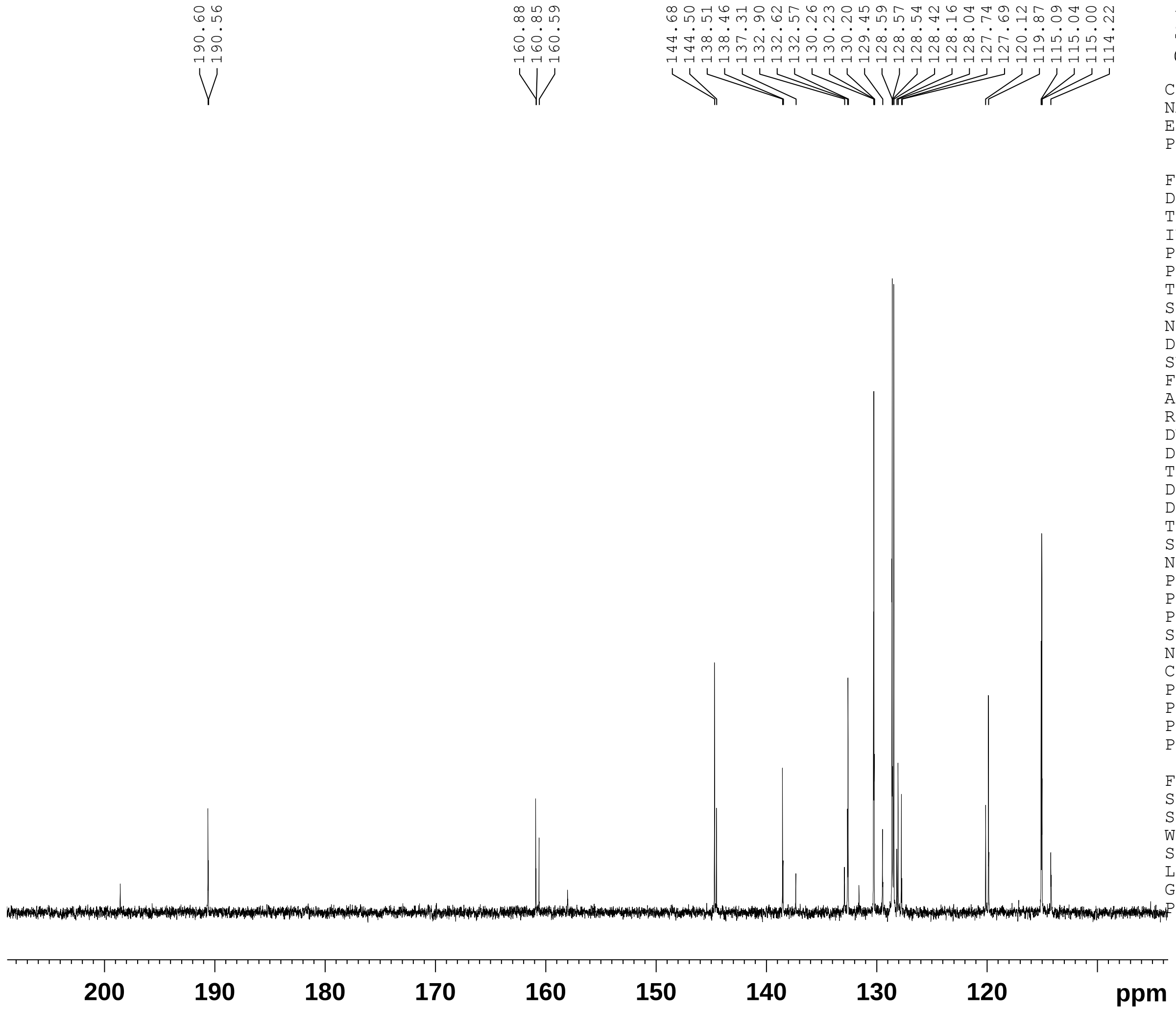

BRUKER
AVANCE NEO
500 MHz NMR SPECTROMETER
SAIF, PANJAB UNIVERSITY,
CHANDIGARH

Current Data Parameters
NAME Jan25-2022-nmr
EXPNO 12
PROCNO 1

F2 - Acquisition Parameters
Date_ 20220127
Time 7.29 h
INSTRUM Avance Neo 500
PROBHD Z119470_0333 (
PULPROG zgpg30
TD 65536
SOLVENT CDCl3
NS 512
DS 4
SWH 37037.035 Hz
FIDRES 1.130281 Hz
AQ 0.8847360 sec
RG 101
DW 13.500 usec
DE 6.50 usec
TE 300.1 K
D1 2.00000000 sec
D11 0.03000000 sec
TD0 1
SFO1 125.7804233 MHz
NUC1 13C
P0 3.33 usec
P1 10.00 usec
PLW1 83.14099884 W
SFO2 500.1720007 MHz
NUC2 1H
CPDPRG[2 waltz65
PCPD2 80.00 usec
PLW2 20.93000031 W
PLW12 0.32703000 W
PLW13 0.16449000 W

F2 - Processing parameters
SI 32768
SF 125.7678465 MHz
WDW EM
SSB 0
LB 1.00 Hz
GB 0
PC 1.40

SAIF,PANJAB UNIVERSITY,CHANDIGARH

SYNAPT-XS#DBA064

22-Sep-2022
11:55:16

PRATIBHA_CH_O_1_DMA 6 (0.137) Cm (6:27)

1: TOF MS ES+
1.06e8

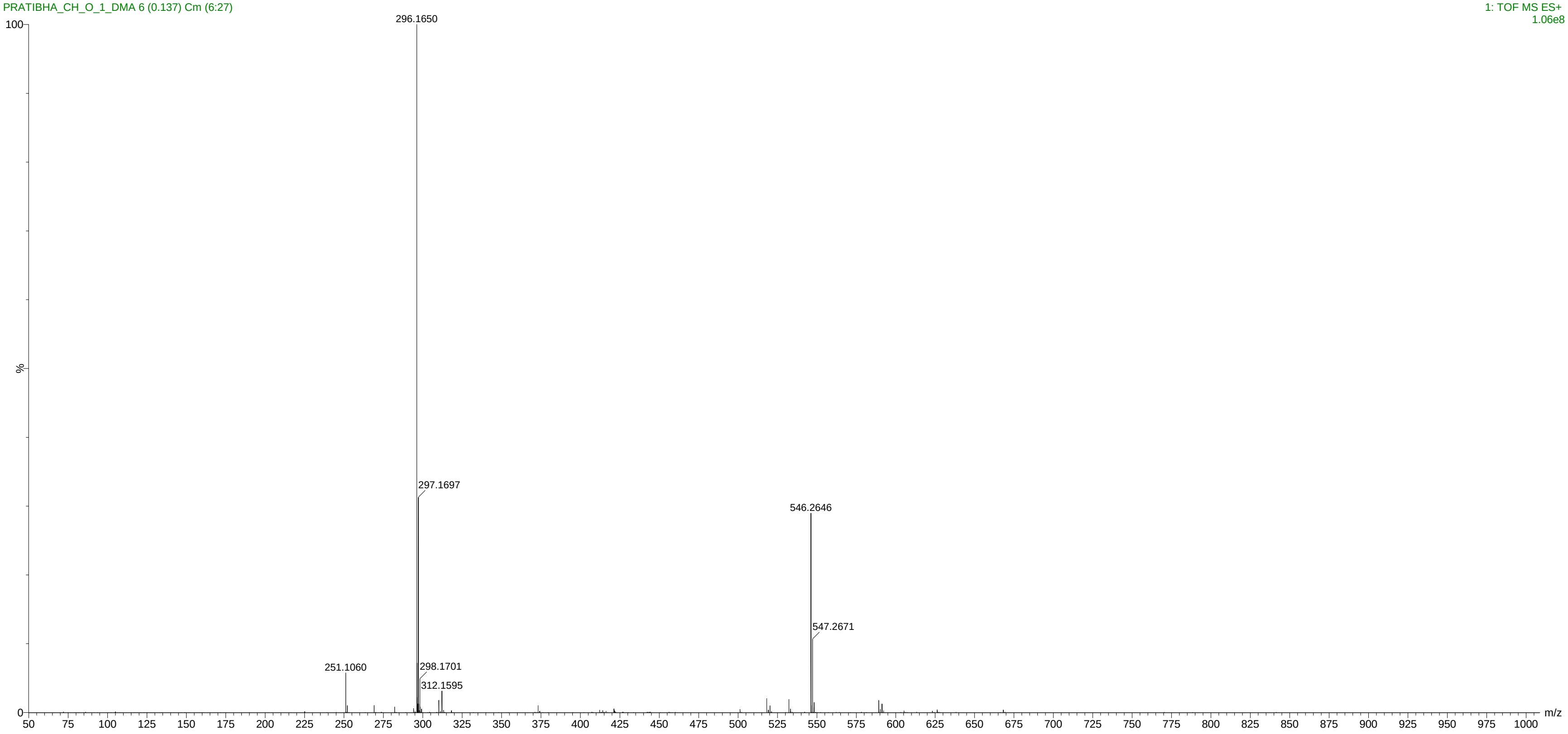

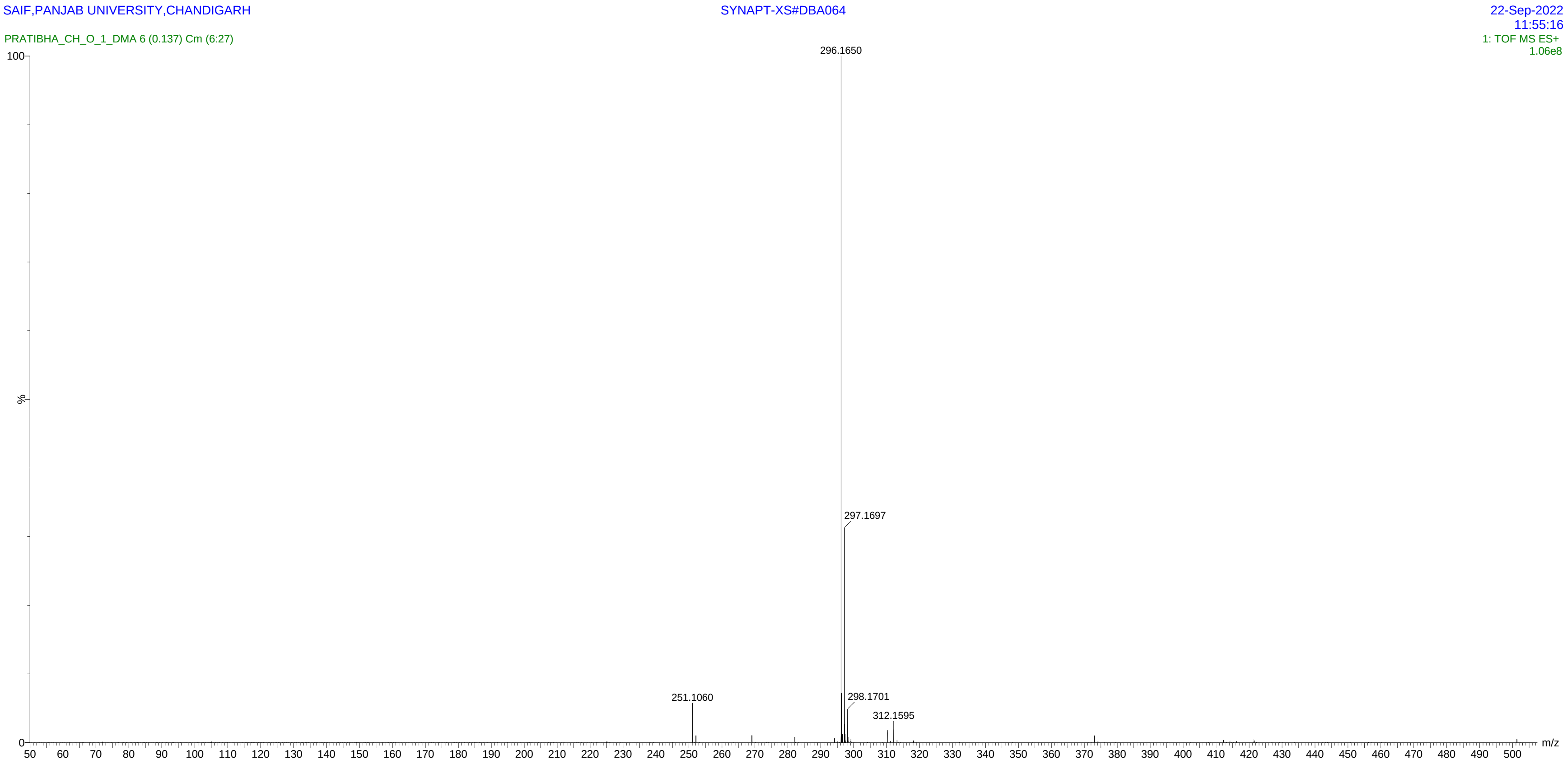

SAIF,PANJAB UNIVERSITY,CHANDIGARH
SYNAPT-XS#DBA064
22-Sep-2022
11:55:16
PRATIBHA_CH_O_1_DMA 6 (0.137) Cm (6:27)
1: TOF MS ES+
1.06e8
296.1650
297.1697
251.1060
298.1701
312.1595
100
%
0
m/z
50 60 70 80 90 100 110 120 130 140 150 160 170 180 190 200 210 220 230 240 250 260 270 280 290 300 310 320 330 340 350 360 370 380 390 400 410 420 430 440 450 460 470 480 490 500

PS7

CH-O-8
1H_8scan CDCl3 {D:\Spectra} nmr 27

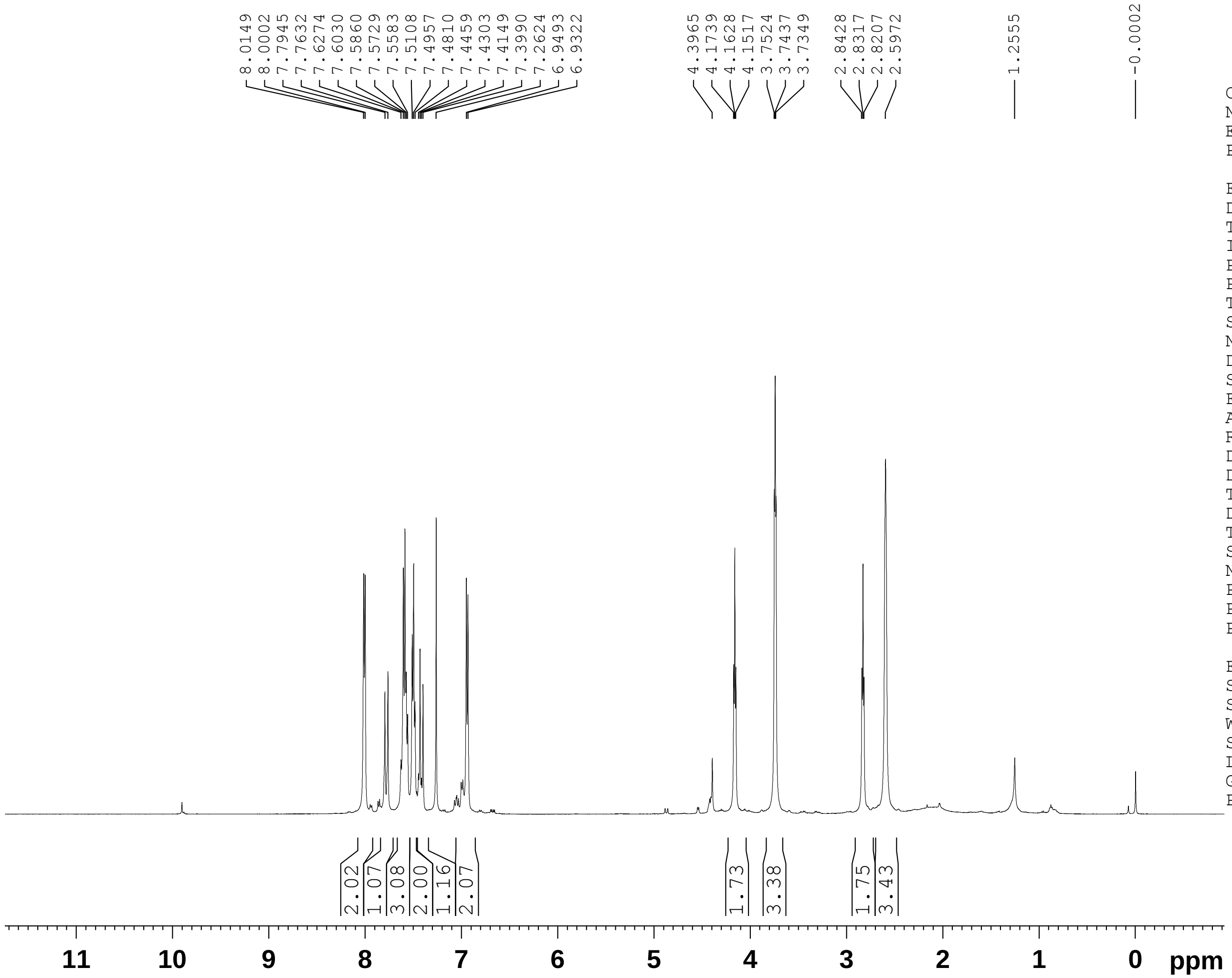

BRUKER
AVANCE NEO
500 MHz NMR
SPECTROMETER
SAIF, P.U.

Current Data Parameters
NAME Mar31-2022
EXPNO 270
PROCNO 1

F2 - Acquisition Parameters
Date_ 20220331
Time_ 18.31 h
INSTRUM Avance Neo 500
PROBHD Z119470_0333 (
PULPROG zg30
TD 65536
SOLVENT CDCl3
NS 16
DS 0
SWH 14705.883 Hz
FIDRES 0.448788 Hz
AQ 2.2282240 sec
RG 95.7854
DW 34.000 usec
DE 6.79 usec
TE 300.2 K
D1 1.00000000 sec
TD0 1
SFO1 500.1730885 MHz
NUC1 1H
P0 3.33 usec
P1 10.00 usec
PLW1 20.93000031 W

F2 - Processing parameters
SI 65536
SF 500.1700109 MHz
WDW EM
SSB 0
LB 0.30 Hz
GB 0
PC 1.00

CH-O-8
1H_8scan CDCl3 {D:\Spectra} nmr 27

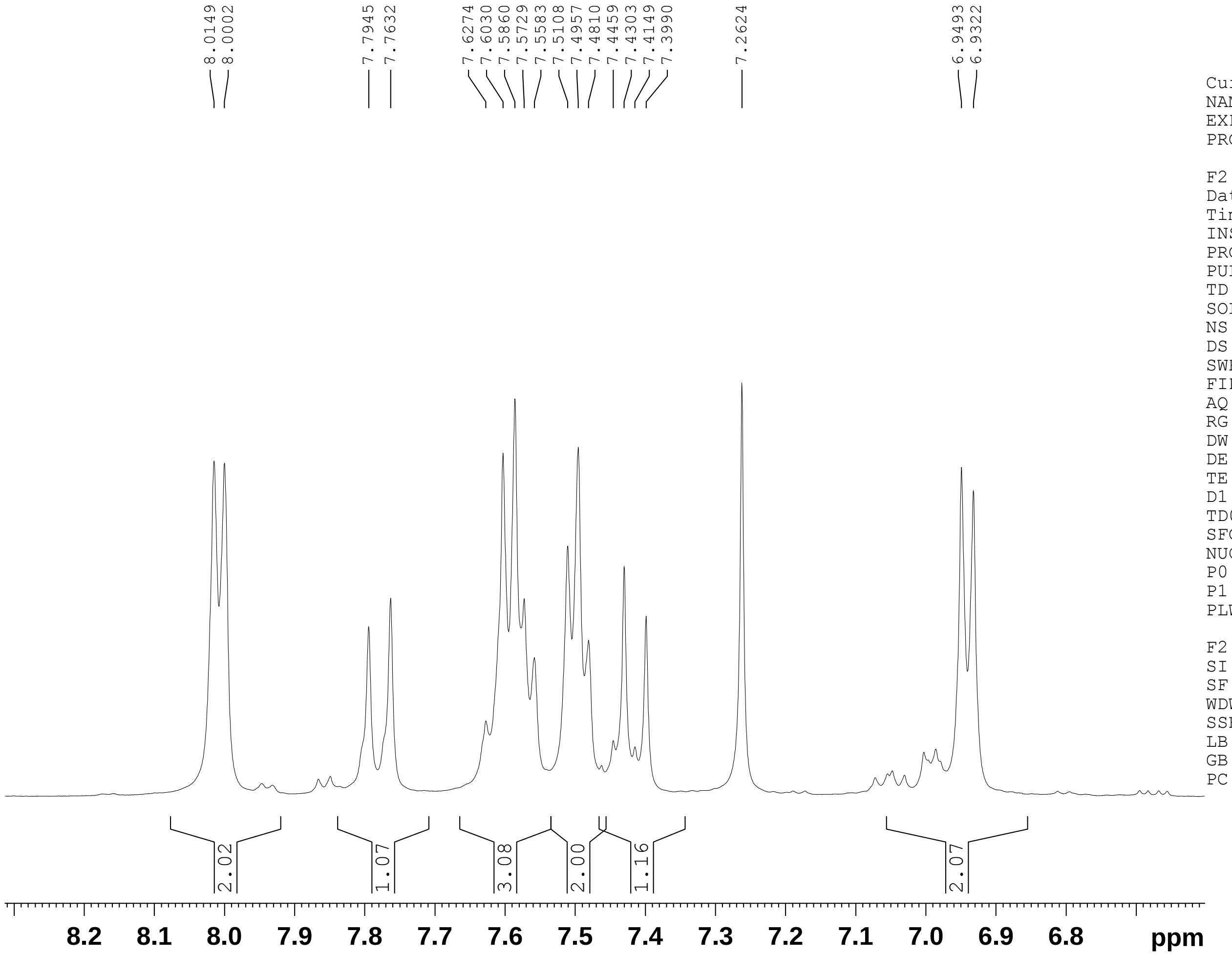

BRUKER
AVANCE NEO
500 MHz NMR
SPECTROMETER
SAIF, P.U.

Current Data Parameters

NAME	Mar31-2022
EXPNO	270
PROCNO	1

F2 - Acquisition Parameters

Date_	20220331
Time	18.31 h
INSTRUM	Avance Neo 500
PROBHD	Z119470_0333 (
PULPROG	zg30
TD	65536
SOLVENT	CDCl3
NS	16
DS	0
SWH	14705.883 Hz
FIDRES	0.448788 Hz
AQ	2.2282240 sec
RG	95.7854
DW	34.000 usec
DE	6.79 usec
TE	300.2 K
D1	1.00000000 sec
TD0	1
SFO1	500.1730885 MHz
NUC1	1H
P0	3.33 usec
P1	10.00 usec
PLW1	20.93000031 W

F2 - Processing parameters

SI	65536
SF	500.1700109 MHz
WDW	EM
SSB	0
LB	0.30 Hz
GB	0
PC	1.00

CH-O-8
1H_8scan CDCl3 {D:\Spectra} nmr 27

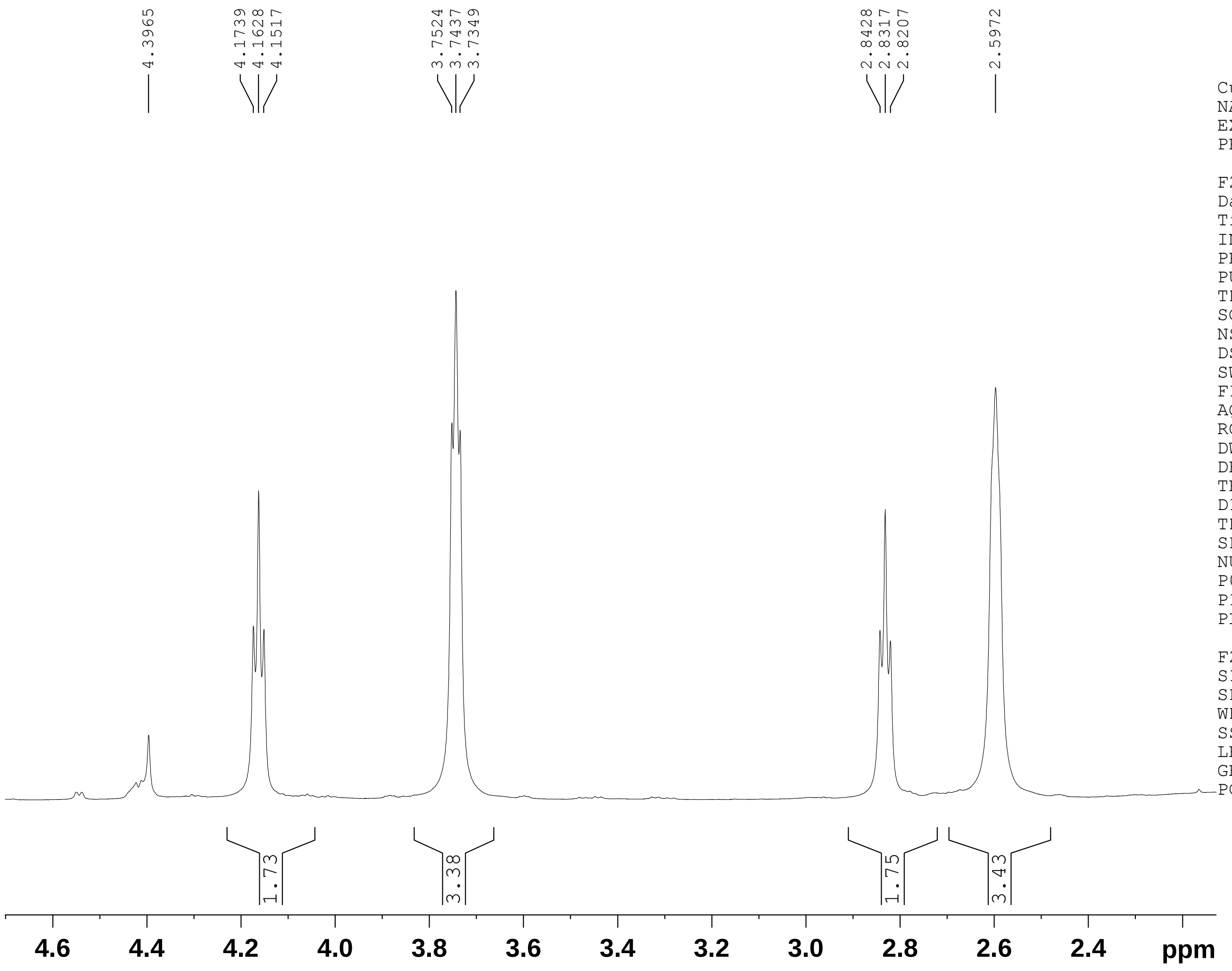

BRUKER
AVANCE NEO
500 MHz NMR
SPECTROMETER
SAIF, P.U.

Current Data Parameters

NAME	Mar31-2022
EXPNO	270
PROCNO	1

F2 - Acquisition Parameters

Date_	20220331
Time	18.31 h
INSTRUM	Avance Neo 500
PROBHD	Z119470_0333 (
PULPROG	zg30
TD	65536
SOLVENT	CDCl3
NS	16
DS	0
SWH	14705.883 Hz
FIDRES	0.448788 Hz
AQ	2.2282240 sec
RG	95.7854
DW	34.000 usec
DE	6.79 usec
TE	300.2 K
D1	1.00000000 sec
TD0	1
SFO1	500.1730885 MHz
NUC1	1H
P0	3.33 usec
P1	10.00 usec
PLW1	20.93000031 W

F2 - Processing parameters

SI	65536
SF	500.1700109 MHz
WDW	EM
SSB	0
LB	0.30 Hz
GB	0
PC	1.00

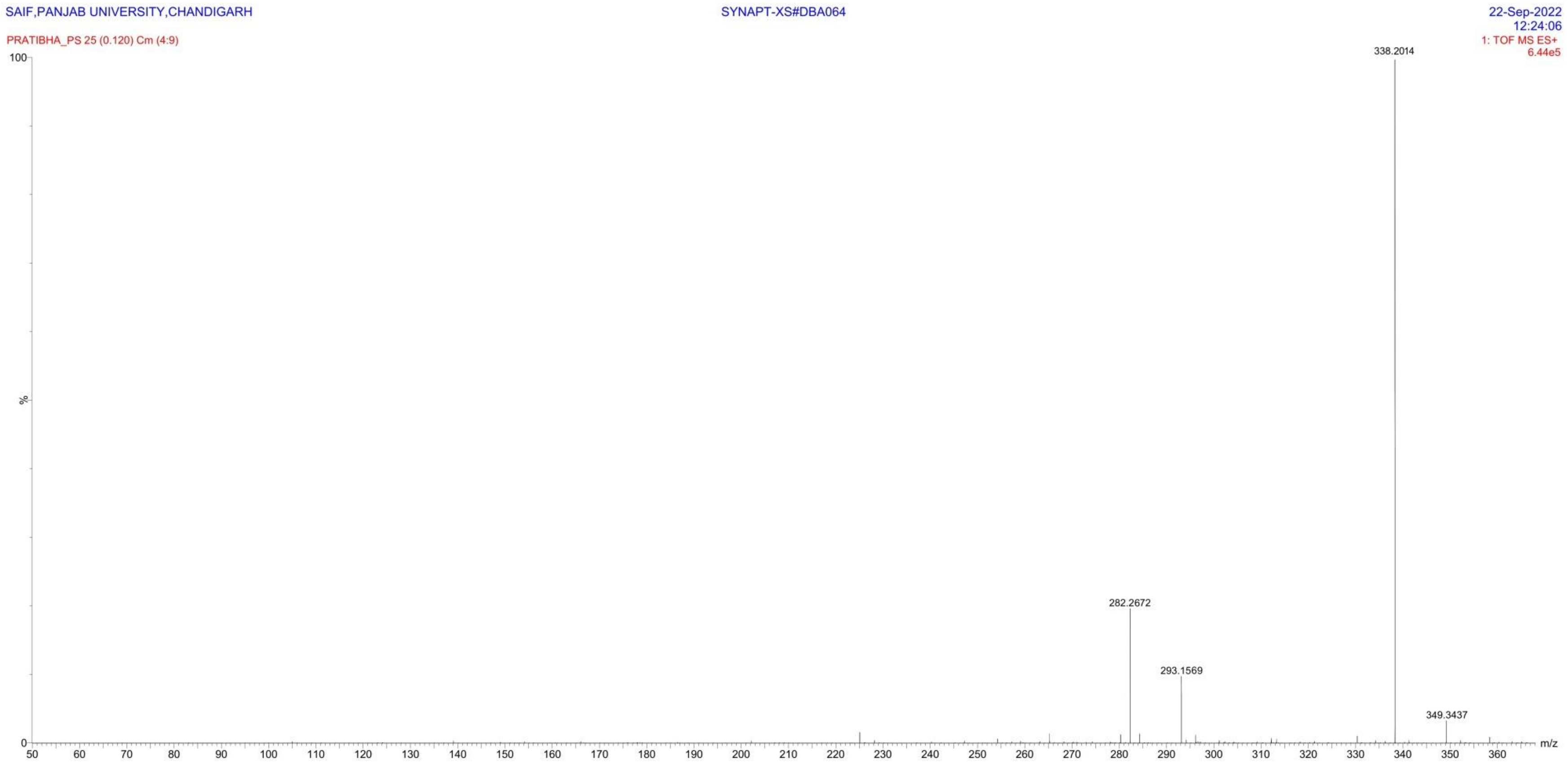
SAIF,PANJAB UNIVERSITY,CHANDIGARH
SYNAPT-XS#DBA064
22-Sep-2022
12:24:06
PRATIBHA_PS 25 (0.120) Cm (4:9)
1: TOF MS ES+
6.44e5
338.2014
282.2672
293.1569
349.3437
%
m/z

PS8

CH-O-9PY
1H_8scan CDCl3 {D:\Spectra} nmr 3

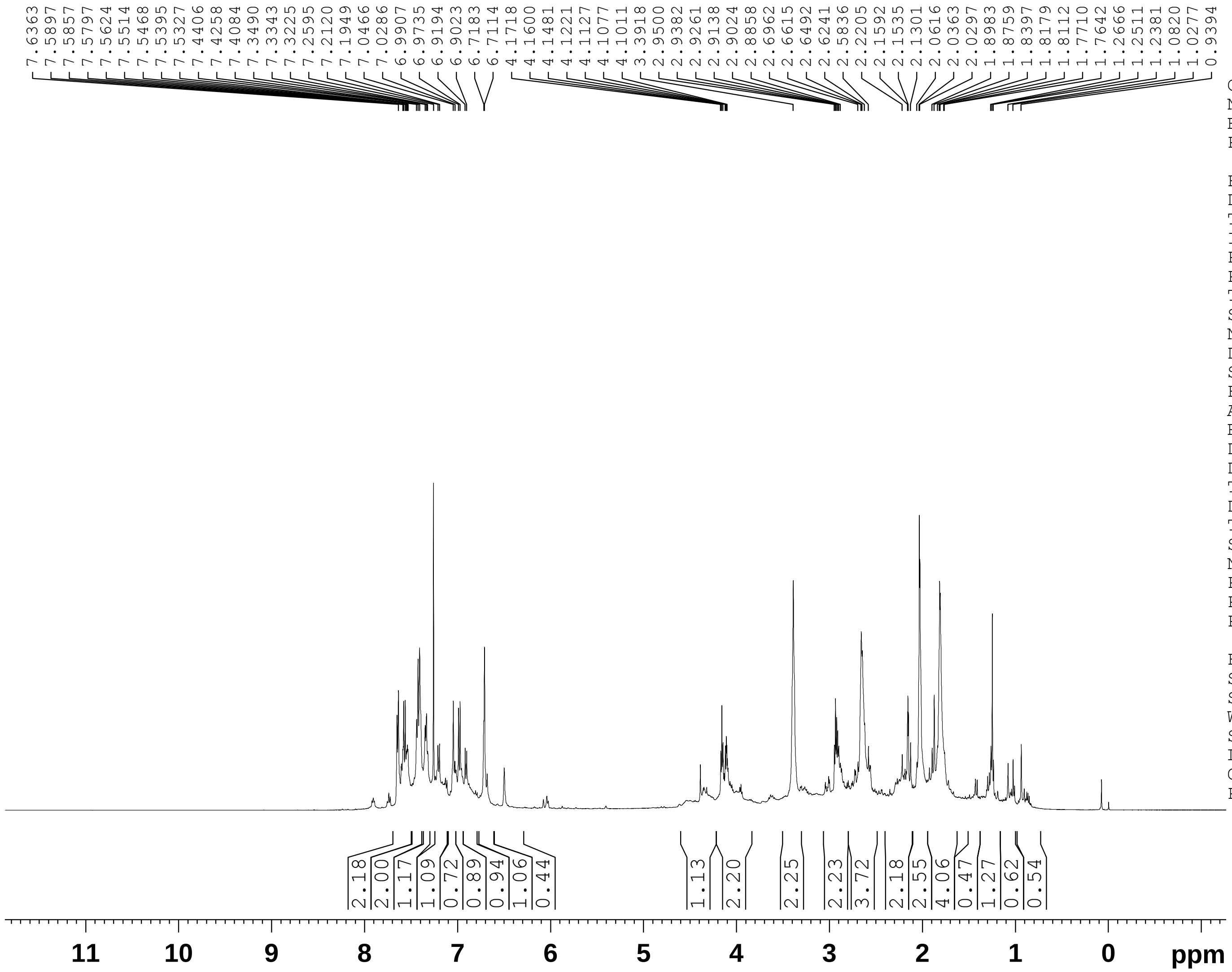

BRUKER
AVANCE NEO
500 MHz NMR
SPECTROMETER
SAIF, P.U.

Current Data Parameters
NAME Jan25-2022-nmr
EXPNO 31
PROCNO 1

F2 - Acquisition Parameters
Date_ 20220127
Time 8.01 h
INSTRUM Avance Neo 500
PROBHD Z119470_0333 (
PULPROG zg30
TD 65536
SOLVENT CDCl3
NS 16
DS 0
SWH 14705.883 Hz
FIDRES 0.448788 Hz
AQ 2.2282240 sec
RG 44.47
DW 34.000 usec
DE 6.79 usec
TE 300.2 K
D1 1.00000000 sec
TD0 1
SFO1 500.1730885 MHz
NUC1 1H
P0 3.33 usec
P1 10.00 usec
PLW1 20.93000031 W

F2 - Processing parameters
SI 65536
SF 500.1700125 MHz
WDW EM
SSB 0
LB 0.30 Hz
GB 0
PC 1.00

CH-O-9PY
C13CPD CDCl3 {D:\Spectra} nmr 3

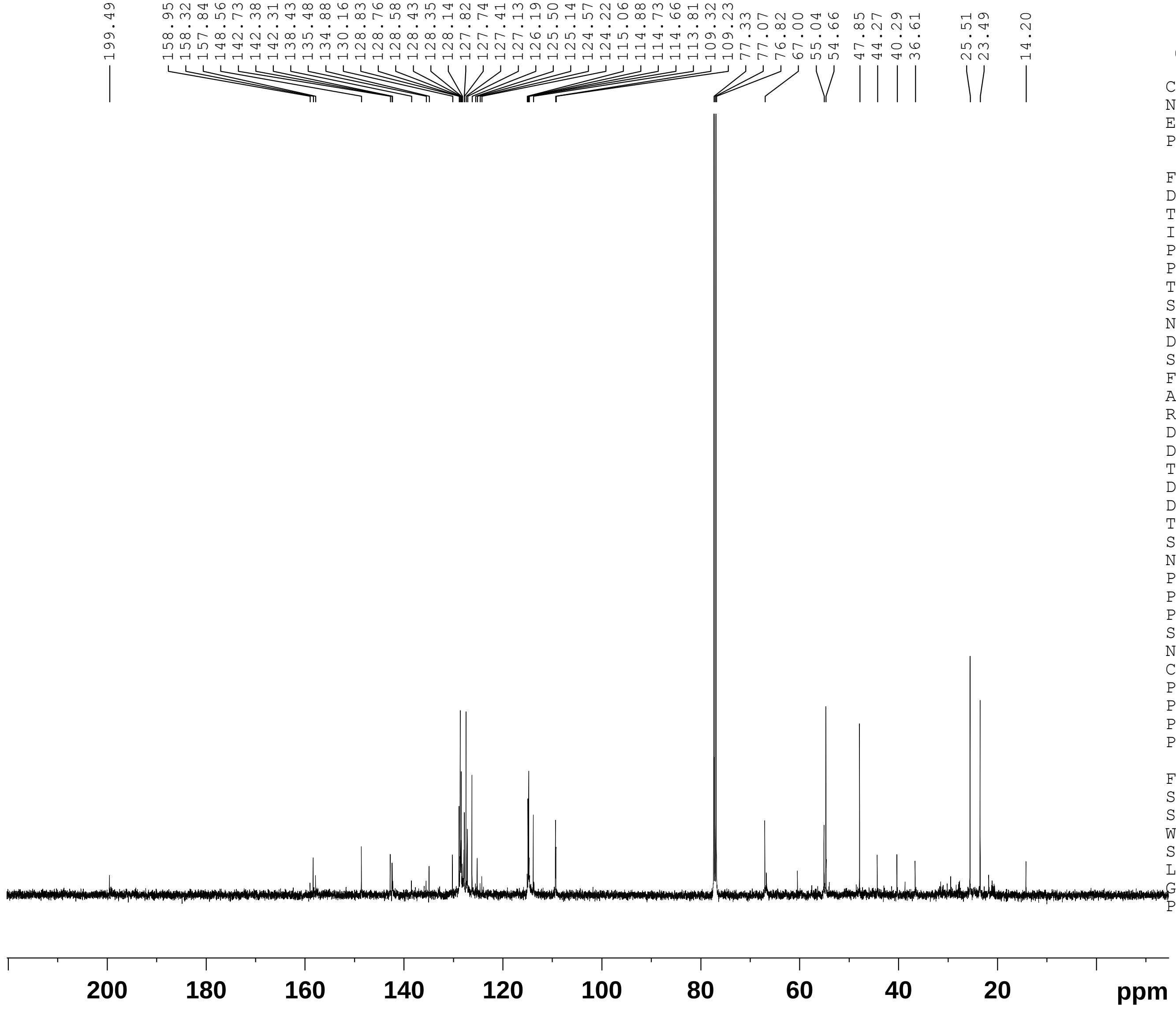

BRUKER
AVANCE NEO
500 MHz NMR SPECTROMETER
SAIF, PANJAB UNIVERSITY,
CHANDIGARH

Current Data Parameters

NAME	Jan25-2022-nmr	
EXPNO	32	
PROCNO	1	

F2 - Acquisition Parameters

Date_	20220127	
Time	8.26	h
INSTRUM	Avance Neo 500	
PROBHD	Z119470_0333 (	
PULPROG	zgpg30	
TD	65536	
SOLVENT	CDCl3	
NS	512	
DS	4	
SWH	37037.035	Hz
FIDRES	1.130281	Hz
AQ	0.8847360	sec
RG	101	
DW	13.500	usec
DE	6.50	usec
TE	300.2	K
D1	2.00000000	sec
D11	0.03000000	sec
TD0	1	
SFO1	125.7804233	MHz
NUC1	13C	
P0	3.33	usec
P1	10.00	usec
PLW1	83.14099884	W
SFO2	500.1720007	MHz
NUC2	1H	
CPDPRG[2	waltz65	
PCPD2	80.00	usec
PLW2	20.93000031	W
PLW12	0.32703000	W
PLW13	0.16449000	W

F2 - Processing parameters

SI	32768	
SF	125.7678465	MHz
WDW	EM	
SSB	0	
LB	1.00	Hz
GB	0	
PC	1.40	

CH-O-9PY
C13CPD CDCl3 {D:\Spectra} nmr 3

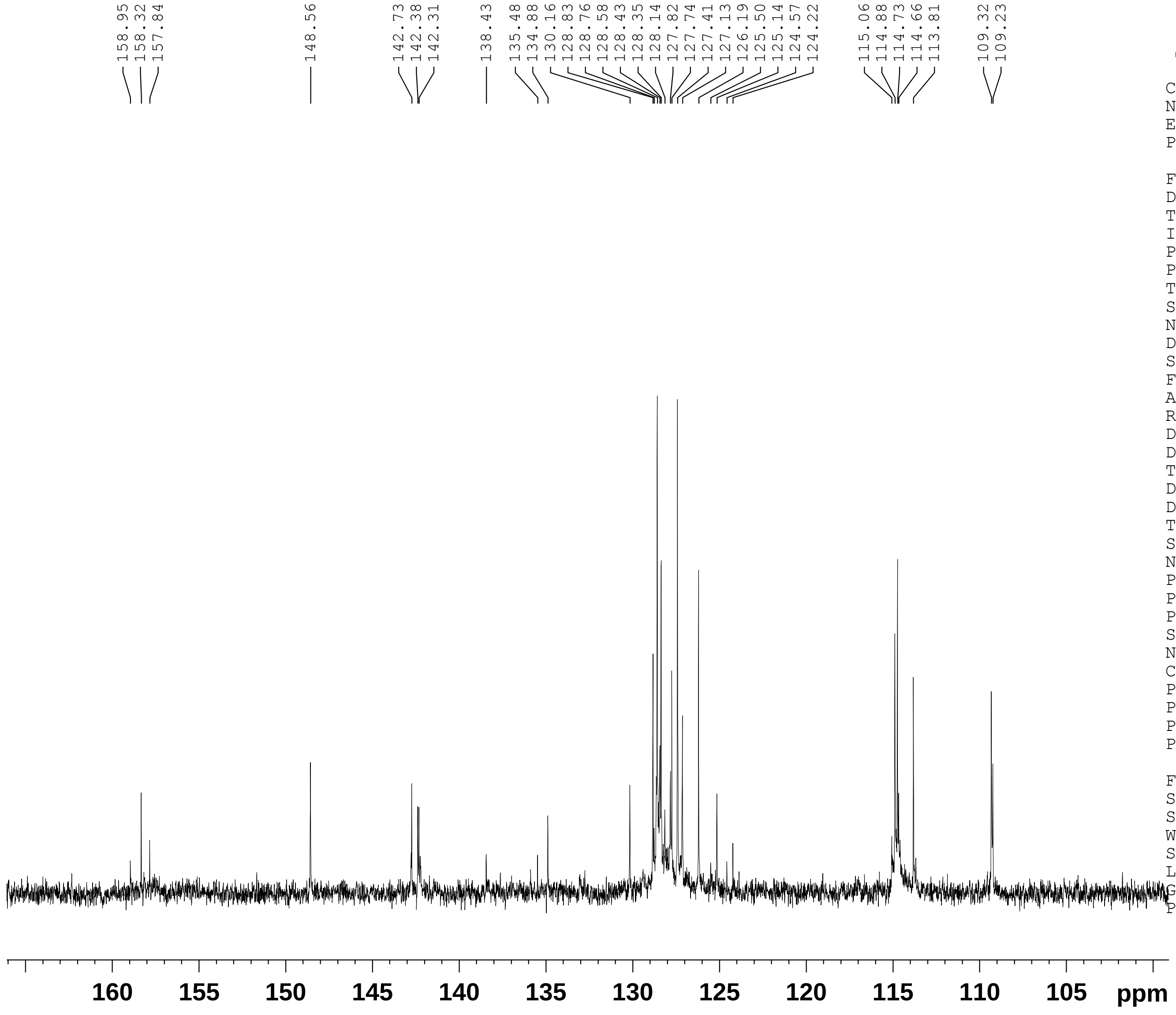

BRUKER
AVANCE NEO
500 MHz NMR SPECTROMETER
SAIF, PANJAB UNIVERSITY,
CHANDIGARH

Current Data Parameters
NAME Jan25-2022-nmr
EXPNO 32
PROCNO 1

F2 - Acquisition Parameters
Date_ 20220127
Time 8.26 h
INSTRUM Avance Neo 500
PROBHD Z119470_0333 (
PULPROG zgpg30
TD 65536
SOLVENT CDCl3
NS 512
DS 4
SWH 37037.035 Hz
FIDRES 1.130281 Hz
AQ 0.8847360 sec
RG 101
DW 13.500 usec
DE 6.50 usec
TE 300.2 K
D1 2.00000000 sec
D11 0.03000000 sec
TD0 1
SFO1 125.7804233 MHz
NUC1 13C
P0 3.33 usec
P1 10.00 usec
PLW1 83.14099884 W
SFO2 500.1720007 MHz
NUC2 1H
CPDPRG[2 waltz65
PCPD2 80.00 usec
PLW2 20.93000031 W
PLW12 0.32703000 W
PLW13 0.16449000 W

F2 - Processing parameters
SI 32768
SF 125.7678465 MHz
WDW EM
SSB 0
LB 1.00 Hz
GB 0
PC 1.40

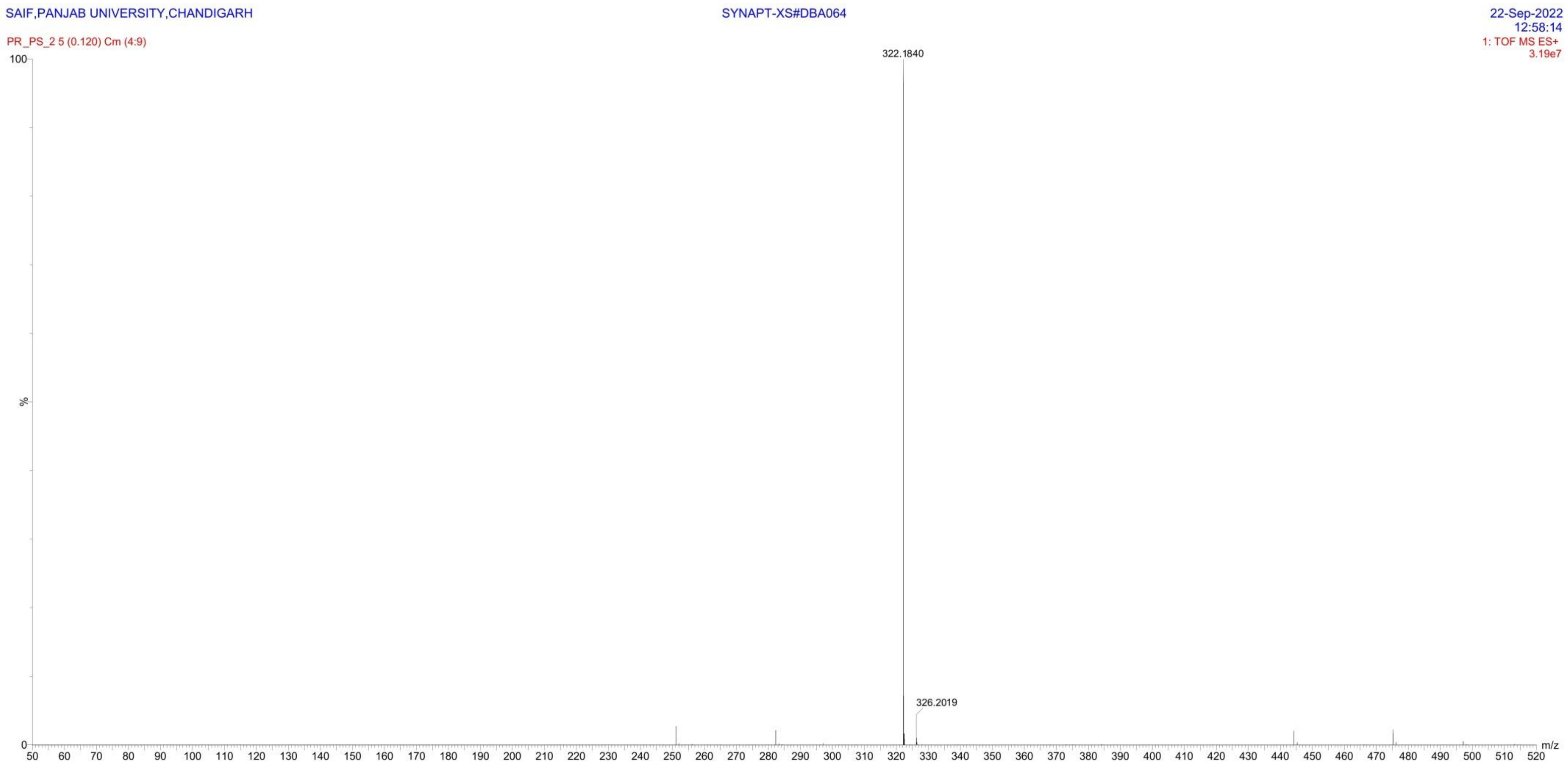
SAIF,PANJAB UNIVERSITY,CHANDIGARH
SYNAPT-XS#DBA064
22-Sep-2022
12:58:14
PR_PS_2 5 (0.120) Cm (4:9)
1: TOF MS ES+
3.19e7
322.1840
326.2019
100
%
0
m/z
50 60 70 80 90 100 110 120 130 140 150 160 170 180 190 200 210 220 230 240 250 260 270 280 290 300 310 320 330 340 350 360 370 380 390 400 410 420 430 440 450 460 470 480 490 500 510 520

PS9

CH-O-10
1H_8scan CDCl3 {D:\Spectra} nmr 28

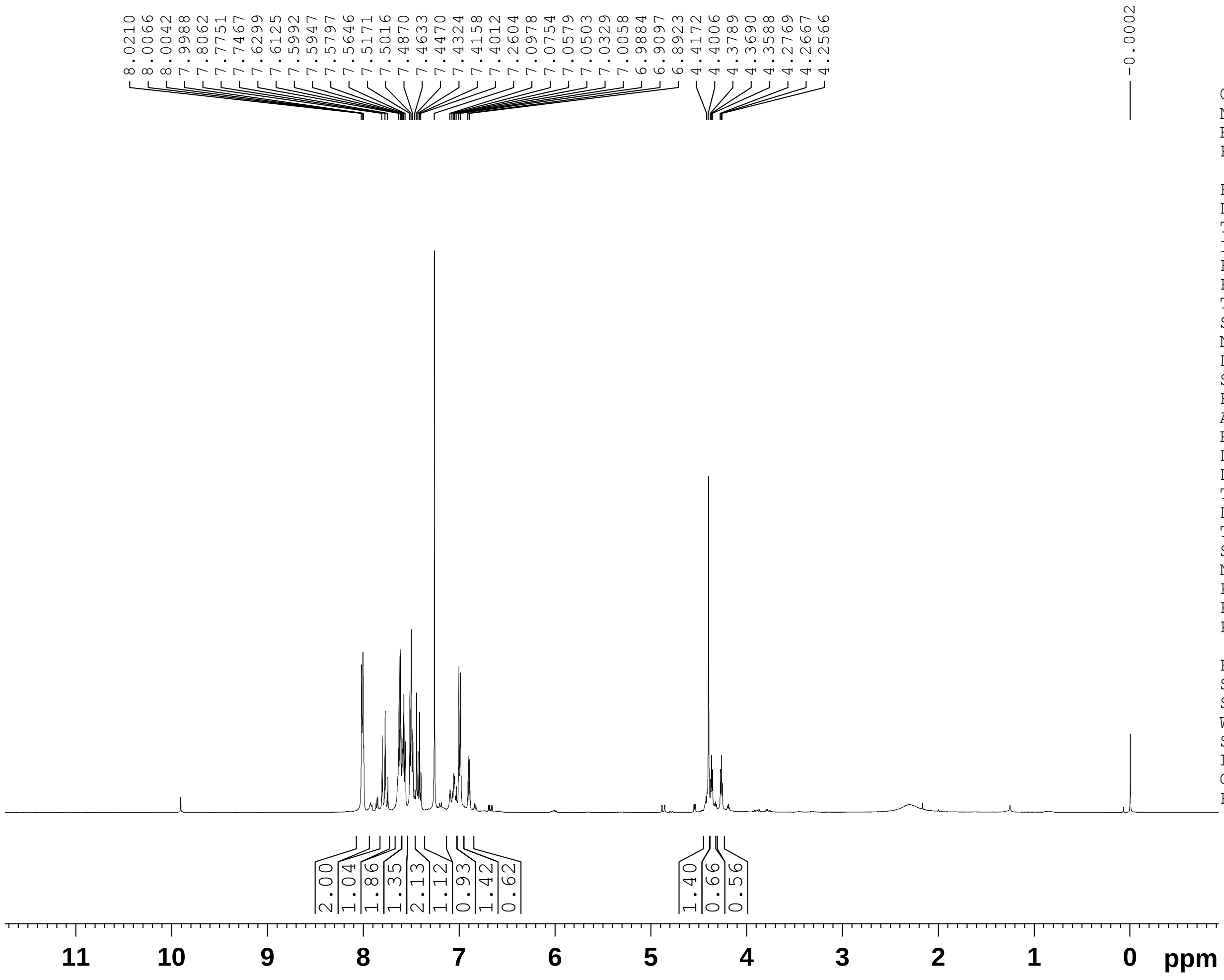

BRUKER
AVANCE NEO
500 MHz NMR
SPECTROMETER
SAIF, P.U.

Current Data Parameters
NAME Mar31-2022
EXPNO 280
PROCNO 1

F2 - Acquisition Parameters
Date_ 20220331
Time 19.25 h
INSTRUM Avance Neo 500
PROBHD Z119470_0333 (
PULPROG zg30
TD 65536
SOLVENT CDCl3
NS 16
DS 0
SWH 14705.883 Hz
FIDRES 0.448788 Hz
AQ 2.2282240 sec
RG 101
DW 34.000 usec
DE 6.79 usec
TE 300.2 K
D1 1.00000000 sec
TD0 1
SFO1 500.1730885 MHz
NUC1 1H
P0 3.33 usec
P1 10.00 usec
PLW1 20.93000031 W

F2 - Processing parameters
SI 65536
SF 500.1700119 MHz
WDW EM
SSB 0
LB 0.30 Hz
GB 0
PC 1.00

CH-O-10
C13CPD CDCl3 {D:\Spectra} nmr 28

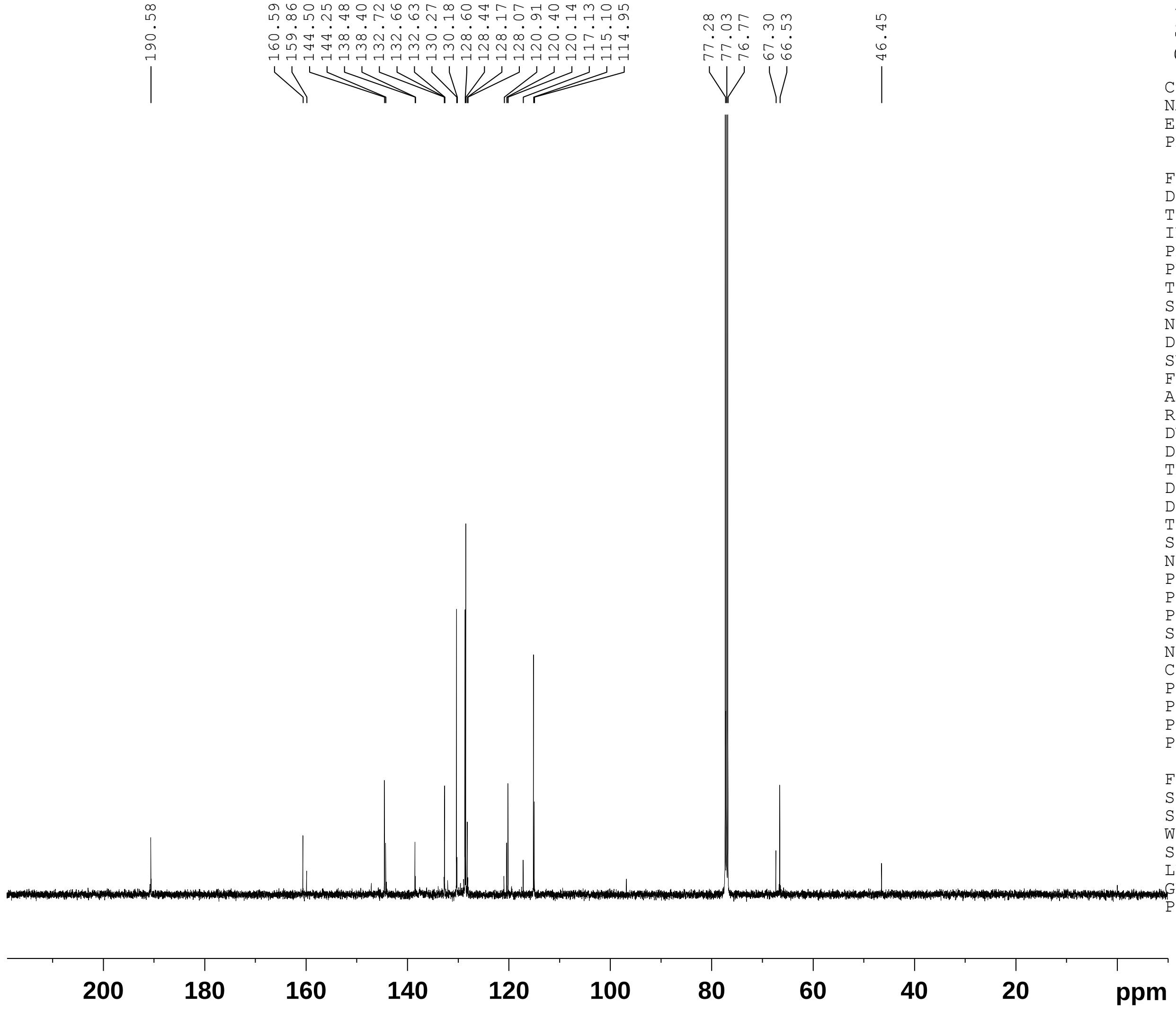

BRUKER
AVANCE NEO
500 MHz NMR SPECTROMETER
SAIF, PANJAB UNIVERSITY,
CHANDIGARH

Current Data Parameters

NAME	Mar31-2022	
EXPNO	281	
PROCNO	1	

F2 - Acquisition Parameters

Date_	20220331	
Time	20.15	h
INSTRUM	Avance Neo 500	
PROBHD	Z119470_0333 (	
PULPROG	zgpg30	
TD	65536	
SOLVENT	CDCl3	
NS	1024	
DS	4	
SWH	37037.035	Hz
FIDRES	1.130281	Hz
AQ	0.8847360	sec
RG	101	
DW	13.500	usec
DE	6.50	usec
TE	300.2	K
D1	2.00000000	sec
D11	0.03000000	sec
TD0	1	
SFO1	125.7804233	MHz
NUC1	13C	
P0	3.33	usec
P1	10.00	usec
PLW1	83.14099884	W
SFO2	500.1720007	MHz
NUC2	1H	
CPDPRG[2	waltz65	
PCPD2	80.00	usec
PLW2	20.93000031	W
PLW12	0.32703000	W
PLW13	0.16449000	W

F2 - Processing parameters

SI	32768	
SF	125.7678465	MHz
WDW	EM	
SSB	0	
LB	1.00	Hz
GB	0	
PC	1.40	

CH-O-10
C13CPD CDCl3 {D:\Spectra} nmr 28

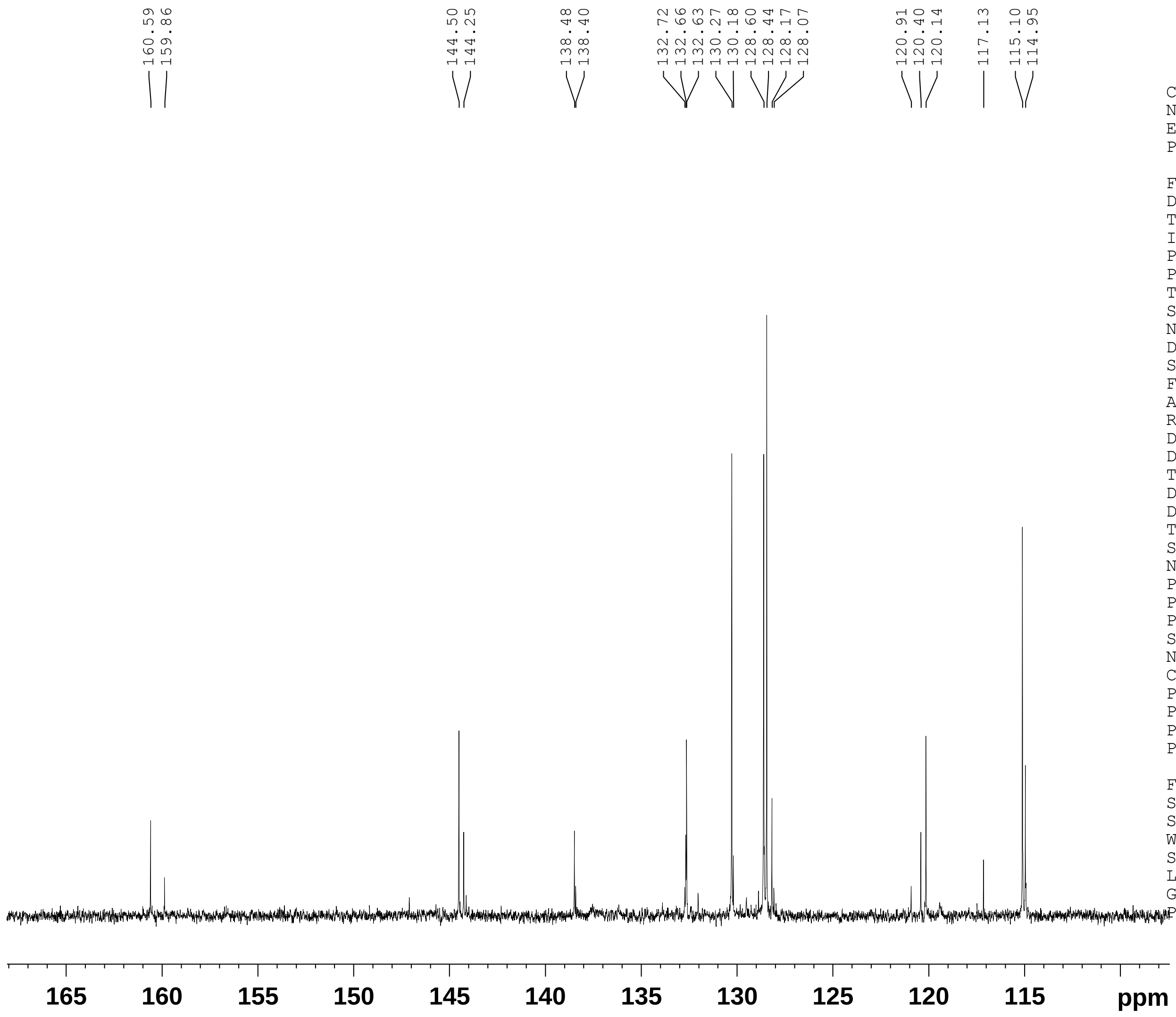

BRUKER
AVANCE NEO
500 MHz NMR SPECTROMETER
SAIF, PANJAB UNIVERSITY,
CHANDIGARH

Current Data Parameters

NAME	Mar31-2022	
EXPNO	281	
PROCNO	1	

F2 - Acquisition Parameters

Date_	20220331	
Time	20.15	h
INSTRUM	Avance Neo 500	
PROBHD	Z119470_0333 (	
PULPROG	zgpg30	
TD	65536	
SOLVENT	CDCl3	
NS	1024	
DS	4	
SWH	37037.035	Hz
FIDRES	1.130281	Hz
AQ	0.8847360	sec
RG	101	
DW	13.500	usec
DE	6.50	usec
TE	300.2	K
D1	2.00000000	sec
D11	0.03000000	sec
TD0	1	
SFO1	125.7804233	MHz
NUC1	13C	
P0	3.33	usec
P1	10.00	usec
PLW1	83.14099884	W
SFO2	500.1720007	MHz
NUC2	1H	
CPDPRG[2	waltz65	
PCPD2	80.00	usec
PLW2	20.93000031	W
PLW12	0.32703000	W
PLW13	0.16449000	W

F2 - Processing parameters

SI	32768	
SF	125.7678465	MHz
WDW	EM	
SSB	0	
LB	1.00	Hz
GB	0	
PC	1.40	

PS10

CH-07MP
1H_8scan CDCl3 {D:\Spectra} nmr 2

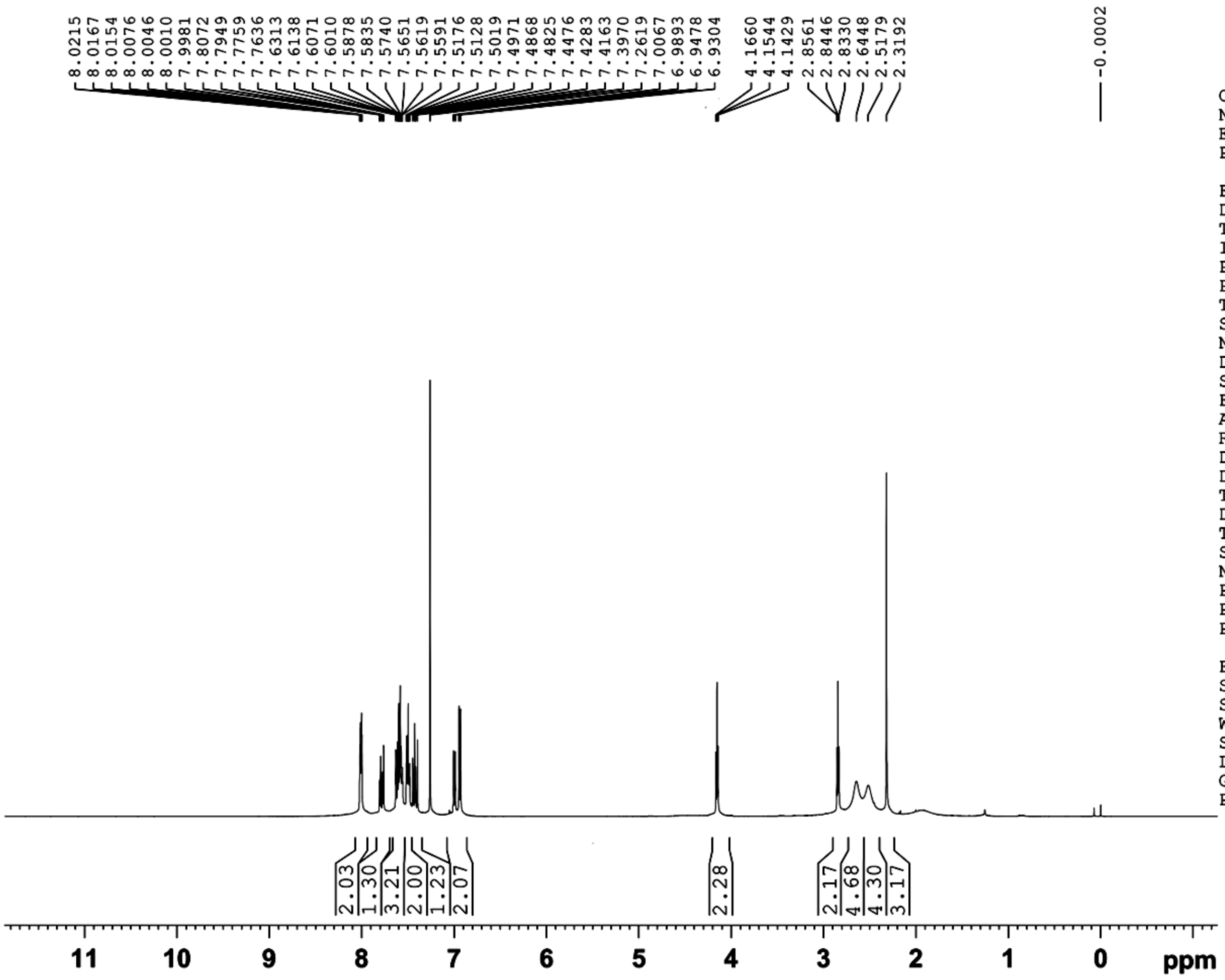

BRUKER
AVANCE NEO
500 MHz NMR
SPECTROMETER
SAIF, P.U.

Current Data Parameters
NAME Jan25-2022-nmr
EXPNO 21
PROCNO 1

F2 - Acquisition Parameters
Date_ 20220127
Time 7.32 h
INSTRUM Avance Neo 500
PROBHD Z119470_0333 (
PULPROG zg30
TD 65536
SOLVENT CDCl3
NS 16
DS 0
SWH 14705.883 Hz
FIDRES 0.448788 Hz
AQ 2.2282240 sec
RG 101
DW 34.000 usec
DE 6.79 usec
TE 300.2 K
D1 1.00000000 sec
TD0 1
SFO1 500.1730885 MHz
NUC1 1H
P0 3.33 usec
P1 10.00 usec
PLW1 20.93000031 W

F2 - Processing parameters
SI 65536
SF 500.1700113 MHz
WDW EM
SSB 0
LB 0.30 Hz
GB 0
PC 1.00

CH-O7MP
C13CPD CDCl3 {D:\Spectra} nmr 2

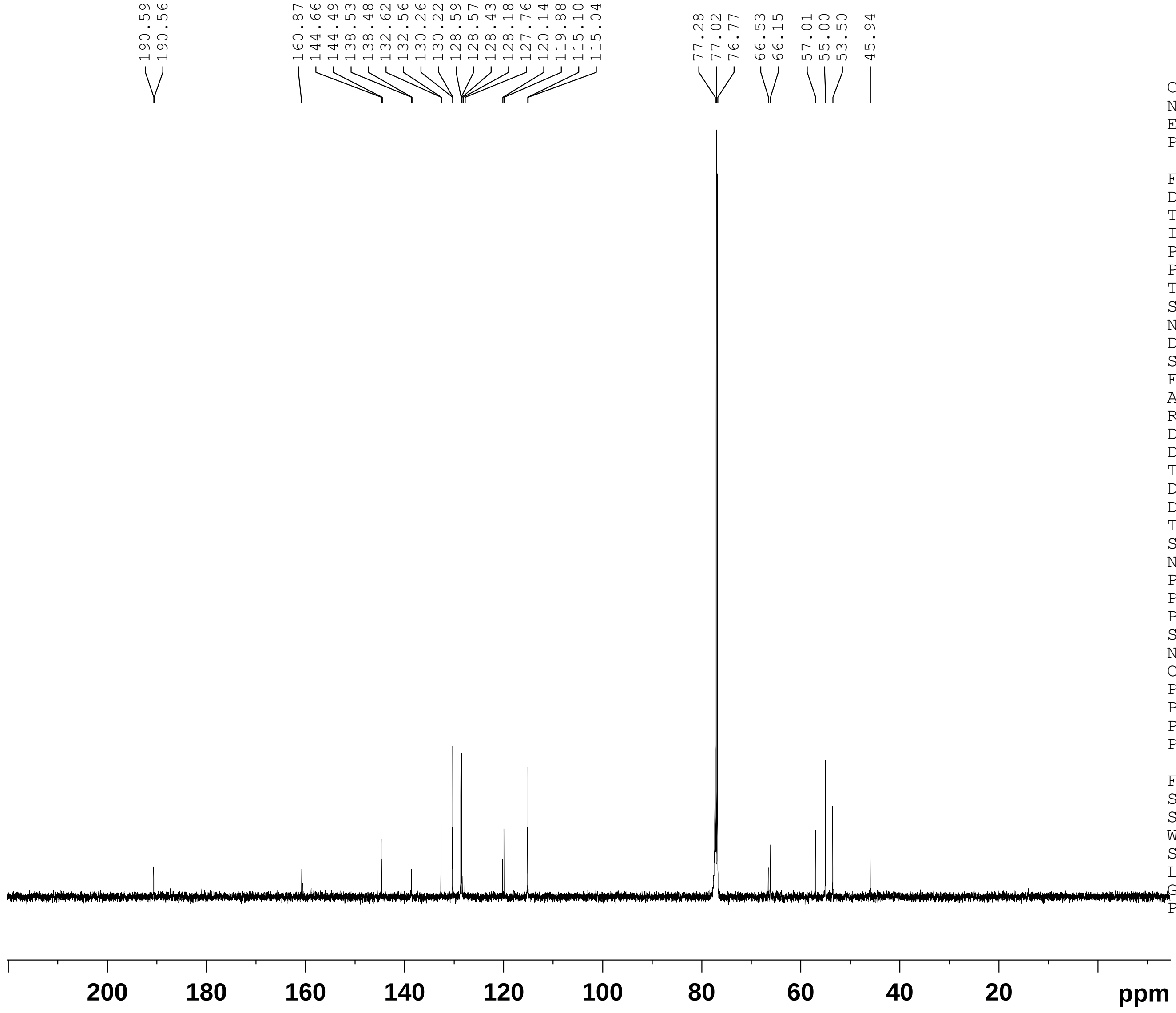

BRUKER
AVANCE NEO
500 MHz NMR SPECTROMETER
SAIF, PANJAB UNIVERSITY,
CHANDIGARH

Current Data Parameters

NAME	Jan25-2022-nmr	
EXPNO	22	
PROCNO	1	

F2 - Acquisition Parameters

Date_	20220127	
Time	7.58	h
INSTRUM	Avance Neo 500	
PROBHD	Z119470_0333 (	
PULPROG	zgpg30	
TD	65536	
SOLVENT	CDCl3	
NS	512	
DS	4	
SWH	37037.035	Hz
FIDRES	1.130281	Hz
AQ	0.8847360	sec
RG	101	
DW	13.500	usec
DE	6.50	usec
TE	300.2	K
D1	2.00000000	sec
D11	0.03000000	sec
TD0	1	
SFO1	125.7804233	MHz
NUC1	13C	
P0	3.33	usec
P1	10.00	usec
PLW1	83.14099884	W
SFO2	500.1720007	MHz
NUC2	1H	
CPDPRG[2	waltz65	
PCPD2	80.00	usec
PLW2	20.93000031	W
PLW12	0.32703000	W
PLW13	0.16449000	W

F2 - Processing parameters

SI	32768	
SF	125.7678465	MHz
WDW	EM	
SSB	0	
LB	1.00	Hz
GB	0	
PC	1.40	

CH-O7MP
C13CPD CDCl3 {D:\Spectra} nmr 2

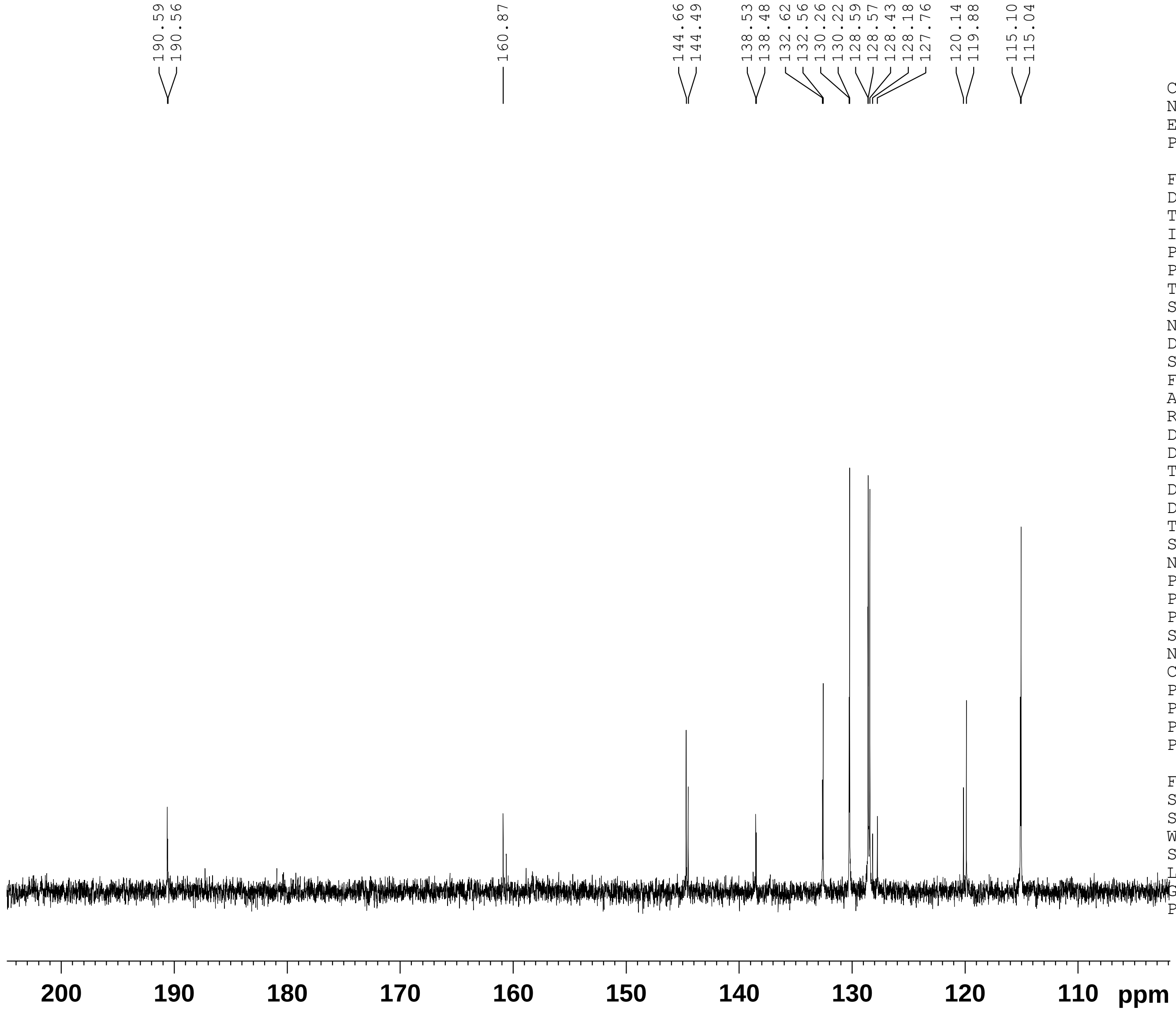

BRUKER
AVANCE NEO
500 MHz NMR SPECTROMETER
SAIF, PANJAB UNIVERSITY,
CHANDIGARH

Current Data Parameters
NAME Jan25-2022-nmr
EXPNO 22
PROCNO 1

F2 - Acquisition Parameters
Date_ 20220127
Time 7.58 h
INSTRUM Avance Neo 500
PROBHD Z119470_0333 (
PULPROG zgpg30
TD 65536
SOLVENT CDCl3
NS 512
DS 4
SWH 37037.035 Hz
FIDRES 1.130281 Hz
AQ 0.8847360 sec
RG 101
DW 13.500 usec
DE 6.50 usec
TE 300.2 K
D1 2.00000000 sec
D11 0.03000000 sec
TD0 1
SFO1 125.7804233 MHz
NUC1 13C
P0 3.33 usec
P1 10.00 usec
PLW1 83.14099884 W
SFO2 500.1720007 MHz
NUC2 1H
CPDPRG[2 waltz65
PCPD2 80.00 usec
PLW2 20.93000031 W
PLW12 0.32703000 W
PLW13 0.16449000 W

F2 - Processing parameters
SI 32768
SF 125.7678465 MHz
WDW EM
SSB 0
LB 1.00 Hz
GB 0
PC 1.40

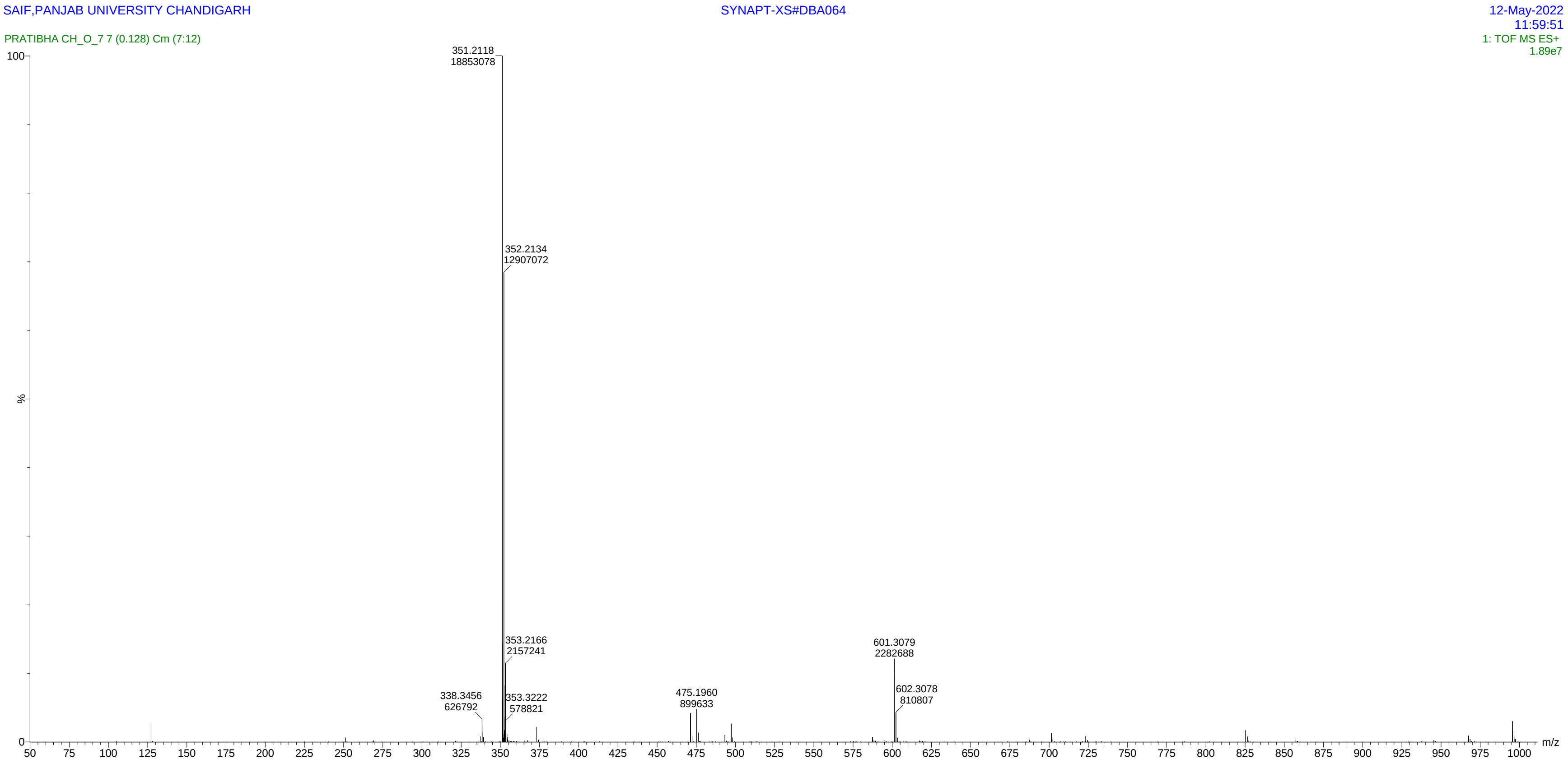
SAIF,PANJAB UNIVERSITY CHANDIGARH
SYNAPT-XS#DBA064
12-May-2022
11:59:51
PRATIBHA CH_O_7 7 (0.128) Cm (7:12)
1: TOF MS ES+
1.89e7
351.2118
18853078
352.2134
12907072
353.2166
2157241
338.3456
626792
353.3222
578821
475.1960
899633
601.3079
2282688
602.3078
810807
%
m/z

SAIF,PANJAB UNIVERSITY CHANDIGARH

SYNAPT-XS#DBA064

12-May-2022
11:59:51

PRATIBHA CH_O_7 7 (0.128) Cm (7:12)

1: TOF MS ES+
1.89e7

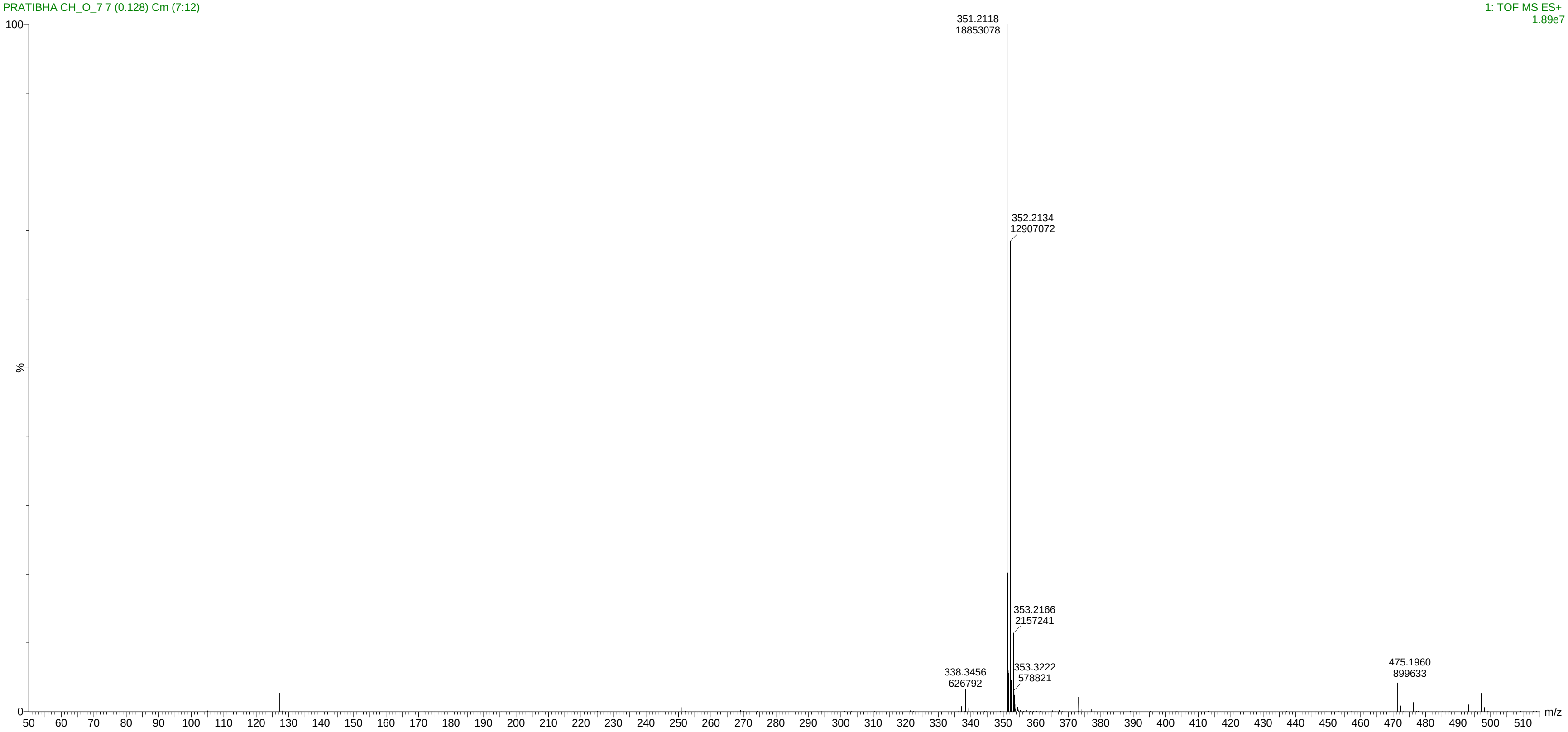

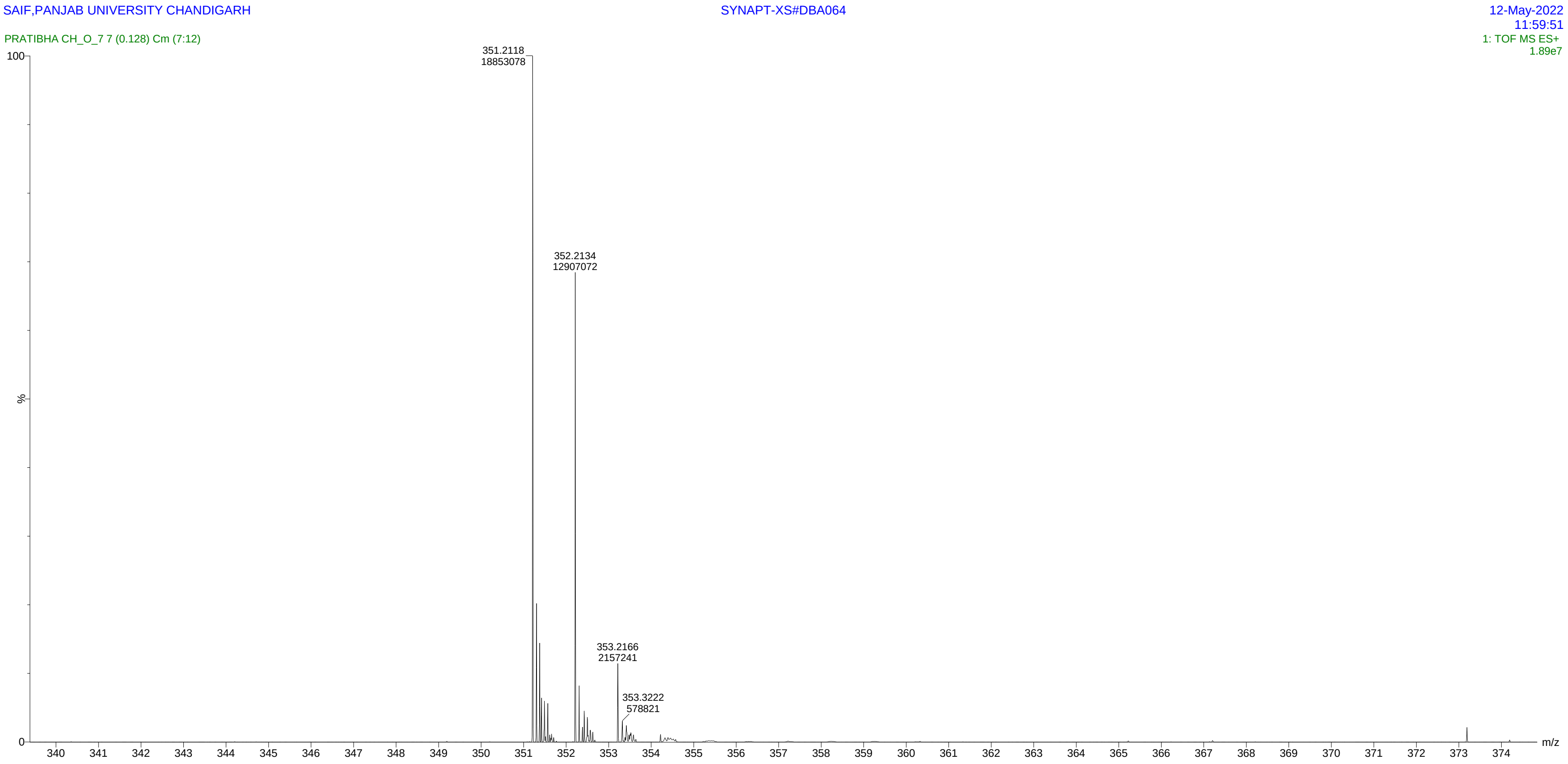

SAIF,PANJAB UNIVERSITY CHANDIGARH
SYNAPT-XS#DBA064
12-May-2022
11:59:51
PRATIBHA CH_O_7 7 (0.128) Cm (7:12)
1: TOF MS ES+
1.89e7
351.2118
18853078
352.2134
12907072
353.2166
2157241
353.3222
578821
100
%
0
340
341
342
343
344
345
346
347
348
349
350
351
352
353
354
355
356
357
358
359
360
361
362
363
364
365
366
367
368
369
370
371
372
373
374
m/z

S.No.	SMILES
1	CN(C)CCOC1CCC(C=CC(=O)C2CCCCC2)CC1
2	CCN(CC)CCOC1=CC=C(C=CC(C2=CC=CC=C2)=O)C=C1
3	CN(CCOC1=CC=C(C=CC(C2=CC=CC=C2)=O)C=C1)CC3=CC=CC=C3
4	O=C(C=CC1=CC=C(OCCN2CCCCC2)C=C1)C3=CC=CC=C3
5	O=C(C=CC1=CC=C(OCCN2CCNCC2)C=C1)C3=CC=CC=C3
6	O=C(C=CC1=CC=C(OCCN2CCOCC2)C=C1)C3=CC=CC=C3
7	O=C(C=CC1=CC=C(OCCN2CCCC2)C=C1)C3=CC=CC=C3
8	O=C(C=CC1=CC=C(OCCN2C=NC=C2)C=C1)C3=CC=CC=C3
9	CN1CCN(CCOC2CCC(C=CC(=O)C3CCCCC3)CC2)CC1
10	O=C(C1=CC=CC=C1)C=CC2=CC=C(OCCCN3CCN(C)CC3)C=C2
11	O=C(C1=CC=CC=C1)C=CC2=CC=C(OCCCN3CCCCC3)C=C2
12	O=C(C=CC1=CC=C(C=C1)OCCCCN2CCN(C)CC2)C3=CC=CC=C3
13	O=C(C=CC1=CC=C(C=C1)OCCCCN2CCCCC2)C3=CC=CC=C3
14	O=C(C=CC1=CC=C(C=C1)OCCCCCN2CCN(C)CC2)C3=CC=CC=C3
15	O=C(C=CC1=CC=C(C=C1)OCCCCCN2CCCCC2)C3=CC=CC=C3

16	O=C(C=CC1=CC=C(C=C1)OCCCCCCN2CCN(C)CC2)C3=CC=CC=C3
17	O=C(C=CC1=CC=C(C=C1)OCCCCCCN2CCCCC2)C3=CC=CC=C3
18	CN1CCN(CCOC2=CC=C(C=CC(C3=CC=C(OC)C=C3)=O)C=C2)CC1
19	CN1CCN(CCOC2=CC=C(C=CC(C3=CC=C(OC)C(OC)=C3)=O)C=C2)CC1
20	CN1CCN(CCOC2=CC=C(C=CC(C3=C(OC)C=C(OC)C=C3OC)=O)C=C2)CC1
21	COC1CCC(CC1)C(=O)C=CC1CCC(CC1)OCCN(C)C
22	CCN(CCOC1CCC(CC1)C=CC(=O)C1CCC(CC1)OC)C
23	COC1CCC(CC1)C(=O)C=CC1CCC(CC1)OCCCN(C)C
24	COC1CCC(CC1)C(=O)C=CC1CCC(CC1)OCN1CCCC1
25	CCCN(CCOC1CCC(CC1)C=CC(=O)C1CCC(CC1)OC)C
26	CCN(CCCOC1CCC(CC1)C=CC(=O)C1CCC(CC1)OC)C
27	CCN(CCOC1CCC(CC1)C=CC(=O)C1CCC(CC1)OC)CC
28	COC1CCC(CC1)C(=O)C=CC1CCC(CC1)OCCCCN(C)C
29	COC1CCC(CC1)C(=O)C=CC1CCC(CC1)OCCN1CCCC1
30	CCN(CCCOC1CCC(CC1)C=CC(=O)C1CCC(CC1)OC)CC
31	COC1CCC(CC1)C(=O)C=CC1CCC(CC1)OCCCN1CCCC1

32	COC1CCC(CC1)C(=O)C=CC1CCC(CC1)OCCN1CCNCC1
33	COC1CCC(CC1)C(=O)C=CC1CCC(CC1)OCCN1CCOCC1
34	COC1CCC(CC1)C(=O)C=CC1CCC(CC1)OCCN1CCCCC1
35	COC1CCC(CC1)C(=O)C=CC1CCC(CC1)OCCCCN1CCCC1
36	COC1CCC(CC1)C(=O)C=CC1CCC(CC1)OCCCN1CCCCC1
37	COC1CCC(CC1)C(=O)C=CC1CCC(CC1)OCCCN1CCOCC1
38	COC1CCC(CC1)C(=O)C=CC1CCC(CC1)OCCN1CCC(CC1)C
39	COC1CCC(CC1)C(=O)C=CC1CCC(CC1)OCCN1CCN(CC1)C
40	COC1CCC(C(C=CC2CCC(OCCN(C)C3CCCCC3)CC2)=O)CC1
41	O=C(C=CC1CCC(OCC2CCCCC2)CC1)C1CCCCC1
42	O=C(C=CC1CCC(OC2CCCCC2)CC1)C1CCCCC1
43	O=C(C=CC1CCC(CC2CCCCC2)CC1)C1CCCCC1
44	CCCCOC1CCC(C=CC(=O)C2CCCCC2)CC1
45	CCCCOC1CCC(C=CC(=O)C2CCCCC2)CC1
46	O=C(C=CC1CCC(NCC2CCCCC2)CC1)C1CCCCC1
47	CC1CCC(OC2CCC(C=CC(=O)C3CCCCC3)CC2)CC1

48 CC1CCC(COC2CCC(C=CC(=O)C3CCCCC3)CC2)CC1

49 O=C(C=CC1CCC(OC2CCC(CL)CC2)CC1)C1CCCCC1

50 CCCCCOC1CCC(C=CC(=O)C2CCCCC2)CC1

51 CCCCCOC1CCC(C=CC(=O)C2CCCCC2)CC1

52 CCCCCOC1CCC(C=CC(=O)C2CCCCC2)CC1

53 COC1CCC(OC2CCC(C=CC(=O)C3CCCCC3)CC2)CC1

54 O=C(C=CC1CCC(OCCC2CCCCC2)CC1)C1CCCCC1

55 O=C(C=CC1CCC(OC2CCC(F)CC2)CC1)C1CCCCC1

56 O=C(C=CC1CCC(OCC2CCC(CL)CC2)CC1)C1CCCCC1

57 O=C(C=CC1CCC(OCC2CCC(F)CC2)CC1)C1CCCCC1

58 COC1CCC(COC2CCC(C=CC(=O)C3CCCCC3)CC2)CC1

59 O=C(C=CC1CCC(OC(=O)C2CCCCC2)CC1)C1CCCCC1

60 COCCOC1CCC(C=CC(=O)C2CCCCC2)CC1

61 CC(=O)C1CCC(-C2CCC(C=CC(=O)C3CCCCC3)CC2)CC1

62 CCCCCCOC1CCC(C=CC(=O)C2CCCCC2)CC1

63 CCCCCCOC1CCC(C=CC(=O)C2CCCCC2)CC1

64 COC1CCCCC1OC1CCC(C=CC(=O)C2CCCCC2)CC1

65 CCCCCC(=O)NC1CCC(C=CC(=O)C2CCCCC2)CC1

66 O=C(C=CC1CCC(OCCOC2CCCCC2)CC1)C1CCCCC1

67 CC1CCCC(OC2CCC(C=CC(=O)C3CCCCC3)CC2)C1

68 CC1CCCC(COC2CCC(C=CC(=O)C3CCCCC3)CC2)C1

69 CC(C)C1CCC(-C2CCC(C=CC(=O)C3CCCCC3)CC2)CC1

70 O=C(C=CC1CCC(CN2CCCCC2)CC1)C1CCCCC1

71 O=C(C=CC1CCC(OC2CCC(BR)CC2)CC1)C1CCCCC1

72 NC(=O)C1CCC(-C2CCC(C=CC(=O)C3CCCCC3)CC2)CC1

73 CC1CCCCC1COC1CCC(C=CC(=O)C2CCCCC2)CC1

74 O=C(C=CC1CCC(OC2CCCCC2F)CC1)C1CCCCC1

75 O=C(O)CCCC1CCC(C=CC(=O)C2CCCCC2)CC1

76 O=C(C=CC1CCC(OCC2CCCC(CL)C2)CC1)C1CCCCC1

77 COC1CCCC(OC2CCC(C=CC(=O)C3CCCCC3)CC2)C1

78 CC(C)CNC(=O)C1CCC(C=CC(=O)C2CCCCC2)CC1

79 O=C(C=CC1CCC(OCC(=O)C2CCCCC2)CC1)C1CCCCC1

80	COC1CCCC(COC2CCC(C=CC(=O)C3CCCCC3)CC2)C1
81	O=C(C=CC1CCC(OCC2CCCC(F)C2)CC1)C1CCCCC1
82	COC(=O)COC1CCC(C=CC(=O)C2CCCCC2)CC1
83	O=C(C=CC1CCC(OCC2CCCCC2F)CC1)C1CCCCC1
84	CC1CCC(CCOC2CCC(C=CC(=O)C3CCCCC3)CC2)CC1
85	COCCCOC1CCC(C=CC(=O)C2CCCCC2)CC1
86	CC(C)CCOC1CCC(C=CC(=O)C2CCCCC2)CC1
87	COC1CCCCC1COC1CCC(C=CC(=O)C2CCCCC2)CC1
88	O=C(C=CC1CCC(SCC2CCCCC2)CC1)C1CCCCC1
89	O=C(O)CCOC1CCC(C=CC(=O)C2CCCCC2)CC1
90	O=C(C=CC1CCC(OCCC2CCC(CL)CC2)CC1)C1CCCCC1
91	COC1CCC(C(=O)OC2CCC(C=CC(=O)C3CCCCC3)CC2)CC1
92	CCOC(=O)COC1CCC(C=CC(=O)C2CCCCC2)CC1
93	O=C(C=CC1CCC(OCCC2CCC(F)CC2)CC1)C1CCCCC1
94	O=C(C=CC1CCC(OCCCCO)CC1)C1CCCCC1
95	COCCCCOC1CCC(C=CC(=O)C2CCCCC2)CC1

96 NC1CCC(OC2CCC(C=CC(=O)C3CCCCC3)CC2)CC1

97 CCOCCOC1CCC(C=CC(=O)C2CCCCC2)CC1

98 CNC(=O)COC1CCC(C=CC(=O)C2CCCCC2)CC1

99 NCCCOC1CCC(C=CC(=O)C2CCCCC2)CC1

100 NCCNC(=O)C1CCC(C=CC(=O)C2CCCCC2)CC1

101 O=C(C=CC1CCC(OCCNC2CCCCC2)CC1)C1CCCCC1

102 O=C(O)CCCCOC1CCC(C=CC(=O)C2CCCCC2)CC1

103 NCCC(=O)NC1CCC(C=CC(=O)C2CCCCC2)CC1

104 CC(C)CCCOC1CCC(C=CC(=O)C2CCCCC2)CC1

105 CN(C)CCCC1CCC(C=CC(=O)C2CCCCC2)CC1

106 CN(C)CCCC1CCC(C=CC(=O)C2CCCCC2)CC1

107 NCCCNC(=O)C1CCC(C=CC(=O)C2CCCCC2)CC1

108 CC1CCCC(CCOC2CCC(C=CC(=O)C3CCCCC3)CC2)C1

109 O=C(C=CC1CCC(CN2CCOCC2)CC1)C1CCCCC1

110 O=C(C=CC1CCC(OCC2CCCNC2)CC1)C1CCCCC1

111 O=C(C=CC1CCC(OCCCCCO)CC1)C1CCCCC1

112	CNCCCC1CCC(C=CC(=O)C2CCCCC2)CC1
113	COCCNCC1CCC(C=CC(=O)C2CCCCC2)CC1
114	COC(=O)CCOC1CCC(C=CC(=O)C2CCCCC2)CC1
115	O=C(C=CC1CCC(SC2CCCCC2)CC1)C1CCCCC1
116	CC(=O)NCCOC1CCC(C=CC(=O)C2CCCCC2)CC1
117	O=C(C=CC1CCC(OC(=O)C2CCCCC2CL)CC1)C1CCCCC1
118	CCOC(=O)CCCC1CCC(C=CC(=O)C2CCCCC2)CC1
119	NC(=O)CCOC1CCC(C=CC(=O)C2CCCCC2)CC1
120	CNCCOC1CCC(C=CC(=O)C2CCCCC2)CC1
121	CN(C)CC(=O)NC1CCC(C=CC(=O)C2CCCCC2)CC1
122	CN(C)CC(=O)NC1CCC(C=CC(=O)C2CCCCC2)CC1
123	CC(C)NC(=O)COC1CCC(C=CC(=O)C2CCCCC2)CC1
124	CC(C)CCCOC1CCC(C=CC(=O)C2CCCCC2)CC1
125	CN(C)CC1CCC(-C2CCC(C=CC(=O)C3CCCCC3)CC2)CC1
126	O=C(C=CC1CCC(OCC2CCCN2)CC1)C1CCCCC1
127	COC1CCCC(C(=O)OC2CCC(C=CC(=O)C3CCCCC3)CC2)C1

128	CCC(=O)OCC1CCC(C=CC(=O)C2CCCCC2)CC1
129	NCCCCCOC1CCC(C=CC(=O)C2CCCCC2)CC1
130	CN1CCN(CC2CCC(C=CC(=O)C3CCCCC3)CC2)CC1
131	O=C(CCC1CCCCC1)OC1CCC(C=CC(=O)C2CCCCC2)CC1
132	COC(=O)CCCOC1CCC(C=CC(=O)C2CCCCC2)CC1
133	CN(C)CCCOC1CCC(C=CC(=O)C2CCCCC2)CC1
134	CN(C)CCCOC1CCC(C=CC(=O)C2CCCCC2)CC1
135	CN(C)CCCOC1CCC(C=CC(=O)C2CCCCC2)CC1
136	CN(C)CCCOC1CCC(C=CC(=O)C2CCCCC2)CC1
137	CN1CCN(C2CCC(C=CC(=O)C3CCCCC3)CC2)CC1
138	CC(=O)C1CCCC(OC2CCC(C=CC(=O)C3CCCCC3)CC2)C1
139	NCCCCNC(=O)C1CCC(C=CC(=O)C2CCCCC2)CC1
140	O=C(C=CC1CCC(-C2CCC(C(F)(F)F)CC2)CC1)C1CCCCC1
141	O=C(C=CC1CCC(OC2CCCNC2)CC1)C1CCCCC1
142	CN(C)CCNC(=O)C1CCC(C=CC(=O)C2CCCCC2)CC1
143	CNCCCOC1CCC(C=CC(=O)C2CCCCC2)CC1

144 CN(C)CCNC(=O)C1CCC(C=CC(=O)C2CCCCC2)CC1

145 CC(=O)OCCOC1CCC(C=CC(=O)C2CCCCC2)CC1

146 CN(C)CCCCC1CCC(C=CC(=O)C2CCCCC2)CC1

147 CN(C)CCCCC1CCC(C=CC(=O)C2CCCCC2)CC1

148 CN(C)CCCCC1CCC(C=CC(=O)C2CCCCC2)CC1

149 CCCC(=O)OC1CCC(C=CC(=O)C2CCCCC2)CC1

150 CCNCCOC1CCC(C=CC(=O)C2CCCCC2)CC1

151 CN(C)C1CCC(C(=O)NC2CCC(C=CC(=O)C3CCCCC3)CC2)CC1

152 CCNCCOC1CCC(C=CC(=O)C2CCCCC2)CC1

153 CCOCCCOC1CCC(C=CC(=O)C2CCCCC2)CC1

154 CCCCC(=O)OC1CCC(C=CC(=O)C2CCCCC2)CC1

155 CC1CCCC(CCCOC2CCC(C=CC(=O)C3CCCCC3)CC2)C1

156 CCOC(=O)CCOC1CCC(C=CC(=O)C2CCCCC2)CC1

157 CC(=O)CCOC1CCC(C=CC(=O)C2CCCCC2)CC1

158 NC1CCCC(OC2CCC(C=CC(=O)C3CCCCC3)CC2)C1

159 COC(=O)C1CCCC(OC2CCC(C=CC(=O)C3CCCCC3)CC2)C1

160 O=C(C=CC1CCC(NCCCO)CC1)C1CCCCC1

161 O=C(C=CC1CCC(CNC2CCCC2)CC1)C1CCCCC1

162 O=C(C=CC1CCC(OCCC2CCC(O)CC2)CC1)C1CCCCC1

163 CNCCCCC1CCC(C=CC(=O)C2CCCCC2)CC1

164 CC(=O)CCCOC1CCC(C=CC(=O)C2CCCCC2)CC1

165 CN(C)C(=O)COC1CCC(C=CC(=O)C2CCCCC2)CC1

166 CNCC1CCC(OC2CCC(C=CC(=O)C3CCCCC3)CC2)CC1

167 CCCCCC(=O)OC1CCC(C=CC(=O)C2CCCCC2)CC1

168 O=C(C=CC1CCC(OCCN2CCCCC2)CC1)C1CCCCC1

169 CC1CCCCC1CCCOC1CCC(C=CC(=O)C2CCCCC2)CC1

170 O=C(C=CC1CCC(OC2CCC([N+](=O)[O-])CC2)CC1)C1CCCCC1

171 CCCNCCOC1CCC(C=CC(=O)C2CCCCC2)CC1

172 CN(C)C1CCC(OC2CCC(C=CC(=O)C3CCCCC3)CC2)CC1

173 O=C(C=CC1CCC(OCCCN2CCCC2)CC1)C1CCCCC1

174 O=C(C=CC1CCC(NCC2CCCNC2)CC1)C1CCCCC1

175 O=C(C=CC1CCC(OC2CCCCN2)CC1)C1CCCCC1

176 N#CC1CCC(OC2CCC(C=CC(=O)C3CCCCC3)CC2)CC1

177 CN(C)CCC(=O)NC1CCC(C=CC(=O)C2CCCCC2)CC1

178 CN(C)CCC(=O)NC1CCC(C=CC(=O)C2CCCCC2)CC1

179 O=C(C=CC1CCC(OCC2CCC([N+](=O)[O-])CC2)CC1)C1CCCCC1

180 CN(C)C1CCCC(-C2CCC(C=CC(=O)C3CCCCC3)CC2)C1

181 CN(C)C1CCC(COC2CCC(C=CC(=O)C3CCCCC3)CC2)CC1

182 O=C(C=CC1CCC(NC2CCCNC2)CC1)C1CCCCC1

183 CN(C)CCCCOC1CCC(C=CC(=O)C2CCCCC2)CC1

184 CN(C)CCCCOC1CCC(C=CC(=O)C2CCCCC2)CC1

185 CN(C)CCCCOC1CCC(C=CC(=O)C2CCCCC2)CC1

186 CC(C)(C)C1CCC(OC2CCC(C=CC(=O)C3CCCCC3)CC2)CC1

187 CCNCCCOC1CCC(C=CC(=O)C2CCCCC2)CC1

188 CN(C)CCCNC(=O)C1CCC(C=CC(=O)C2CCCCC2)CC1

189 O=C(C=CC1CCC(CCCCN2CCCC2)CC1)C1CCCCC1

190 NCCCNC1CCC(C=CC(=O)C2CCCCC2)CC1

191 CNCCCCOC1CCC(C=CC(=O)C2CCCCC2)CC1

192	O=C(C=CC1CCC(C(=O)NCCN2CCCC2)CC1)C1CCCCC1
193	CN(C)CCCCCC1CCC(C=CC(=O)C2CCCCC2)CC1
194	NC(=O)C1CCCC(NC2CCC(C=CC(=O)C3CCCCC3)CC2)C1
195	CN(C)CCCCCC1CCC(C=CC(=O)C2CCCCC2)CC1
196	CNC(=O)CCOC1CCC(C=CC(=O)C2CCCCC2)CC1
197	CC(=O)CCCCOC1CCC(C=CC(=O)C2CCCCC2)CC1
198	O=C(C=CC1CCC(NC2CCNCC2)CC1)C1CCCCC1
199	CNC(=O)OCC1CCC(C=CC(=O)C2CCCCC2)CC1
200	CN(C)CC1CCCC(-C2CCC(C=CC(=O)C3CCCCC3)CC2)C1
201	NCCCC(=O)C1CCC(C=CC(=O)C2CCCCC2)CC1
202	CSCCOC1CCC(C=CC(=O)C2CCCCC2)CC1
203	CN(C)CC1CCC(OC2CCC(C=CC(=O)C3CCCCC3)CC2)CC1
204	CN(C)CCNC1CCC(C=CC(=O)C2CCCCC2)CC1
205	CN(C)CCNC1CCC(C=CC(=O)C2CCCCC2)CC1
206	O=C(C=CC1CCC(OCCN2CCCCCC2)CC1)C1CCCCC1
207	CNC(=O)CCCOC1CCC(C=CC(=O)C2CCCCC2)CC1

208 O=C(C=CC1CCC(OC(=O)C2CCC2)CC1)C1CCCCC1

209 O=C(C=CC1CCC(OCCCN2CCCCC2)CC1)C1CCCCC1

210 O=C(C=CC1CCC(OCC2CCCS2)CC1)C1CCCCC1

211 CCNC(=O)OC1CCC(C=CC(=O)C2CCCCC2)CC1

212 O=C(C=CC1CCC(OCCC2CCCNC2)CC1)C1CCCCC1

213 CC(C)OCCOC1CCC(C=CC(=O)C2CCCCC2)CC1

214 O=C(C=CC1CCC(OCCCCN2CCCC2)CC1)C1CCCCC1

215 CN(C)C1CCC(C(=O)C2CCC(C=CC(=O)C3CCCCC3)CC2)CC1

216 O=C(CCN1CCCCC1)NC1CCC(C=CC(=O)C2CCCCC2)CC1

217 O=C(C=CC1CCC(C(=O)NCCN2CCCCC2)CC1)C1CCCCC1

218 O=C(C=CC1CCC(OCC2CCC(C(F)(F)F)CC2)CC1)C1CCCCC1

219 COCC(=O)OC1CCC(C=CC(=O)C2CCCCC2)CC1

220 CN(C)CCNCC1CCC(C=CC(=O)C2CCCCC2)CC1

221 CN(C)C1CCC(CCC2CCC(C=CC(=O)C3CCCCC3)CC2)CC1

222 O=C(C=CC1CCC(NCCN2CCCC2)CC1)C1CCCCC1

223 O=C(C=CC1CCC(OC(=O)C2CCCNC2)CC1)C1CCCCC1

224 O=C(C=CC1CCC(OC2CCCCCC2)CC1)C1CCCCC1

225 CN1CCN(CC2CCCC(C=CC(=O)C3CCCCC3)C2)CC1

226 CSCCCOC1CCC(C=CC(=O)C2CCCCC2)CC1

227 CCN(CC)CCOC1CCC(C=CC(=O)C2CCCCC2)CC1

228 COC(=O)CCCNC1CCC(C=CC(=O)C2CCCCC2)CC1

229 CN(C)CC1CCCCC1-C1CCC(C=CC(=O)C2CCCCC2)CC1

230 CN(C)CCCNC1CCC(C=CC(=O)C2CCCCC2)CC1

231 O=C(C=CC1CCC(OCCOCCO)CC1)C1CCCCC1

232 CC(C)(C)CCOC1CCC(C=CC(=O)C2CCCCC2)CC1

233 CN(C)CCC(=O)C1CCC(C=CC(=O)C2CCCCC2)CC1

234 CC(C)(C)OC(=O)COC1CCC(C=CC(=O)C2CCCCC2)CC1

235 CNCC1CCCCC1OC1CCC(C=CC(=O)C2CCCCC2)CC1

236 CN1CCN(C(=O)C2CCCC(C=CC(=O)C3CCCCC3)C2)CC1

237 O=C(C=CC1CCC(OC2CCC(N3CCCCC3)CC2)CC1)C1CCCCC1

238 CN(C)CCCOC(=O)C1CCC(C=CC(=O)C2CCCCC2)CC1

239 CN(C)C1CCCC(OC2CCC(C=CC(=O)C3CCCCC3)CC2)C1

240 CCN(C)CCOC1CCC(C=CC(=O)C2CCCCC2)CC1

241 N#CCCOC1CCC(C=CC(=O)C2CCCCC2)CC1

242 CN(C)C1CCCC(OC2CCC(C=CC(=O)C3CCCCC3)CC2)C1

243 CCN(C)CCOC1CCC(C=CC(=O)C2CCCCC2)CC1

244 COC(=O)CSC1CCC(C=CC(=O)C2CCCCC2)CC1

245 CN(C)CCCC(=O)C1CCC(C=CC(=O)C2CCCCC2)CC1

246 CN(C)C(=O)CCOC1CCC(C=CC(=O)C2CCCCC2)CC1

247 O=C(C=CC1CCC(NCCCN2CCCC2)CC1)C1CCCCC1

248 CN(C)CCOCC1CCC(C=CC(=O)C2CCCCC2)CC1

249 CN(CCOC1CCC(C=CC(=O)C2CCCCC2)CC1)C1CCCCC1

250 CN(C)CC1CCCC(OC2CCC(C=CC(=O)C3CCCCC3)CC2)C1

251 CNCCCNC1CCC(C=CC(=O)C2CCCCC2)CC1

252 O=C(C=CC1CCC(NCCCCL)CC1)C1CCCCC1

253 CCN(C)CCCC1CCC(C=CC(=O)C2CCCCC2)CC1

254 CN1CCC(C2CCC(C=CC(=O)C3CCCCC3)CC2)CC1

255 O=C(C=CC1CCC(NCCC2CCNCC2)CC1)C1CCCCC1

256 O=C(C=CC1CCC(NC(C2CCCCC2)C2CCCCC2)CC1)C1CCCCC1

257 CC(C)NC(=O)OC1CCC(C=CC(=O)C2CCCCC2)CC1

258 O=C(C=CC1CCC(OCCNCCO)CC1)C1CCCCC1

259 CN1CCN(CCOC2CCC(C=CC(=O)C3CCCCC3)CC2)CC1

260 COC1CCCC(CC(=O)OC2CCC(C=CC(=O)C3CCCCC3)CC2)C1

261 CCN(CC)C(=O)COC1CCC(C=CC(=O)C2CCCCC2)CC1

262 CCN(CC)CCCOC1CCC(C=CC(=O)C2CCCCC2)CC1

263 O=C(C=CC1CCC(NCCC2CCCCN2)CC1)C1CCCCC1

264 CN(C)CCCCNC1CCC(C=CC(=O)C2CCCCC2)CC1

265 CN(C)CCCCNC1CCC(C=CC(=O)C2CCCCC2)CC1

266 CN(C)CCCCNC1CCC(C=CC(=O)C2CCCCC2)CC1

267 CC1CCN(CCOC2CCC(C=CC(=O)C3CCCCC3)CC2)CC1

268 CC1CCC(S(=O)(=O)OC2CCC(C=CC(=O)C3CCCCC3)CC2)CC1

269 CN(C)CC1CCCC(COC2CCC(C=CC(=O)C3CCCCC3)CC2)C1

270 CN(C)CCCCC(=O)C1CCC(C=CC(=O)C2CCCCC2)CC1

271 CN(C)CCCCC(=O)C1CCC(C=CC(=O)C2CCCCC2)CC1

272 O=C(C=CC1CCC(OCCCN2CCOCC2)CC1)C1CCCCC1

273 CC(C)(C)CCCOC1CCC(C=CC(=O)C2CCCCC2)CC1

274 O=C(C=CC1CCCCC1)OC1CCC(C=CC(=O)C2CCCCC2)CC1

275 CN(C)CC1CCCCC1OC1CCC(C=CC(=O)C2CCCCC2)CC1

276 CCN(C)CCCOC1CCC(C=CC(=O)C2CCCCC2)CC1

277 CCN(C)CCCOC1CCC(C=CC(=O)C2CCCCC2)CC1

278 CCN(C)CCCOC1CCC(C=CC(=O)C2CCCCC2)CC1

279 O=C(C=CC1CCC(NCCCN2CCCCC2)CC1)C1CCCCC1

280 CS(=O)(=O)CCCOC1CCC(C=CC(=O)C2CCCCC2)CC1

281 CCC(=O)CCCOC1CCC(C=CC(=O)C2CCCCC2)CC1

282 O=C(C=CC1CCC(OCC(=O)N2CCOCC2)CC1)C1CCCCC1

283 O=C(C=CC1CCC(OCN2CCCC2)CC1)C1CCCCC1

284 O=C(C=CC1CCC(CCCCN2CCOCC2)CC1)C1CCCCC1

285 O=C(C=CC1CCC(OC(=O)C2CCCO2)CC1)C1CCCCC1

286 CCCN(C)CCOC1CCC(C=CC(=O)C2CCCCC2)CC1

287 O=C(C=CC1CCC(C(=O)NCCN2CCOCC2)CC1)C1CCCCC1

288 CN(C)CCCC1CCCC(-C2CCC(C=CC(=O)C3CCCCC3)CC2)C1

289 O=C(C=CC1CCC(OCC2CCC3C(C2)OCO3)CC1)C1CCCCC1

290 CC(C)=CCOC1CCC(C=CC(=O)C2CCCCC2)CC1

291 CN(C)C1CCCCC1OC1CCC(C=CC(=O)C2CCCCC2)CC1

292 CN(C)C1CCCCC1OC1CCC(C=CC(=O)C2CCCCC2)CC1

293 O=C(C=CC1CCC(SC2CCC(O)CC2)CC1)C1CCCCC1

294 CC(C)(C)NCCOC1CCC(C=CC(=O)C2CCCCC2)CC1

295 O=C(C=CC1CCC(-C2CNN(CC3CCCCC3)C2)CC1)C1CCCCC1

296 O=C(C=CC1CCC(OC2CCC(N3CCOCC3)CC2)CC1)C1CCCCC1

297 CC1CCNC(OC2CCC(C=CC(=O)C3CCCCC3)CC2)C1

298 C=C(C)CCCOC1CCC(C=CC(=O)C2CCCCC2)CC1

299 CSCCCNC1CCC(C=CC(=O)C2CCCCC2)CC1

300 CC(C)N(C)CCOC1CCC(C=CC(=O)C2CCCCC2)CC1

301 CC(C)N(C)CCOC1CCC(C=CC(=O)C2CCCCC2)CC1

302 NC1CCN(C2CCC(C=CC(=O)C3CCCCC3)CC2)CC1

303 O=C(C=CC1CCC(OCCS(=O)(=O)O)CC1)C1CCCCC1

304	CN(C)CCOC(=O)NC1CCC(C=CC(=O)C2CCCCC2)CC1
305	CCN(CC)CCNC1CCC(C=CC(=O)C2CCCCC2)CC1
306	CN(C)CCC(=O)OC1CCC(C=CC(=O)C2CCCCC2)CC1
307	CN(C)CCC(=O)OC1CCC(C=CC(=O)C2CCCCC2)CC1
308	CN(C)CCC(=O)OC1CCC(C=CC(=O)C2CCCCC2)CC1
309	CN(C)CCC(=O)OC1CCC(C=CC(=O)C2CCCCC2)CC1
310	O=C(C=CC1CCC(-C2CCC(CO)O2)CC1)C1CCCCC1
311	CN1CCN(CCOC2CCCC(C=CC(=O)C3CCCCC3)C2)CC1
312	CN1CCC(COC2CCC(C=CC(=O)C3CCCCC3)CC2)CC1
313	O=C(C=CC1CCC(NCCN2CCOCC2)CC1)C1CCCCC1
314	CC(C)NCCCNC1CCC(C=CC(=O)C2CCCCC2)CC1
315	CN(C)CCCC(=O)OC1CCC(C=CC(=O)C2CCCCC2)CC1
316	O=C(C=CC1CCC(NCC(C2CCCCC2)C2CCCCC2)CC1)C1CCCCC1
317	CN(C)CCCC1CCCCC1-C1CCC(C=CC(=O)C2CCCCC2)CC1
318	CCN(CC)C(=O)OC1CCC(C=CC(=O)C2CCCCC2)CC1
319	CN1CCC(OC2CCC(C=CC(=O)C3CCCCC3)CC2)CC1

320 O=C(C=CC1CCC(OC(=O)N2CCOCC2)CC1)C1CCCCC1

321 O=C(C=CC1CCC(C(=O)NC2CCC(O)CC2)CC1)C1CCCCC1

322 CN(C)CC(=O)OC1CCC(C=CC(=O)C2CCCCC2)CC1

323 CC(C)(O)CCCOC1CCC(C=CC(=O)C2CCCCC2)CC1

324 COC(=O)C1CC(OC2CCC(C=CC(=O)C3CCCCC3)CC2)CCC1O

325 CN(C)CC(=O)OC1CCC(C=CC(=O)C2CCCCC2)CC1

326 CN(C)CC(=O)OC1CCC(C=CC(=O)C2CCCCC2)CC1

327 CN(C)CC(=O)OC1CCC(C=CC(=O)C2CCCCC2)CC1

328 CN(C)CCC1CCCCC1OC1CCC(C=CC(=O)C2CCCCC2)CC1

329 CCN1CCC(OC2CCC(C=CC(=O)C3CCCCC3)CC2)CC1

330 COC(=O)C1CCC(OC2CCC(C=CC(=O)C3CCCCC3)CC2)CC1O

331 CCN1CCC(OC2CCC(C=CC(=O)C3CCCCC3)CC2)CC1

332 O=C(C=CC1CCC(OCCN2CCCC2=O)CC1)C1CCCCC1

333 CN(CCO)CCOC1CCC(C=CC(=O)C2CCCCC2)CC1

334 O=C(C=CC1CCC(NC2CCOCC2)CC1)C1CCCCC1

335 CCSCCCOC1CCC(C=CC(=O)C2CCCCC2)CC1

336	CN1CCN(C(=O)OC2CCC(C=CC(=O)C3CCCCC3)CC2)CC1
337	CN1CCN(C(=O)OC2CCC(C=CC(=O)C3CCCCC3)CC2)CC1
338	CCN(CC)CCCNC1CCC(C=CC(=O)C2CCCCC2)CC1
339	CN(C)C1CCCCC1CNC1CCC(C=CC(=O)C2CCCCC2)CC1
340	CC(=O)OC1CCCCC1C(=O)OC1CCC(C=CC(=O)C2CCCCC2)CC1
341	CC(C)N(C)CCCOC1CCC(C=CC(=O)C2CCCCC2)CC1
342	CC(C)N(C)CCCOC1CCC(C=CC(=O)C2CCCCC2)CC1
343	CN(C)CCN1CCN(C2CCC(C=CC(=O)C3CCCCC3)CC2)CC1
344	CC(=O)N(C)CCCOC1CCC(C=CC(=O)C2CCCCC2)CC1
345	CN1CC(COC2CCC(C=CC(=O)C3CCCCC3)CC2)C1
346	CN(CCCOC1CCC(C=CC(=O)C2CCCCC2)CC1)C1CC1
347	CC(=O)N(C)CCCOC1CCC(C=CC(=O)C2CCCCC2)CC1
348	O=C(C=CC1CCC(NCCCN2CCOCC2)CC1)C1CCCCC1
349	CN(C)C(=O)NCCOC1CCC(C=CC(=O)C2CCCCC2)CC1
350	CN(C)CC1CCC(-C2CCC(C=CC(=O)C3CCCCC3)CC2)NC1
351	CN1CCN(CCCOC2CCCC(C=CC(=O)C3CCCCC3)C2)CC1

352 CN(C)C1CCCCC1C(=O)OC1CCC(C=CC(=O)C2CCCCC2)CC1

353 O=C(C=CC1CCC(OC2CCOCC2)CC1)C1CCCCC1

354 O=C(C=CC1CCC(OCCC(=O)N2CCOCC2)CC1)C1CCCCC1

355 CN(C)CCSC1CCC(C=CC(=O)C2CCCCC2)CC1

356 CN(C)CCSC1CCC(C=CC(=O)C2CCCCC2)CC1

357 CN(C)CCNC(=O)OC1CCC(C=CC(=O)C2CCCCC2)CC1

358 O=C(C=CC1CCC(OCCN2CCNCC2)CC1)C1CCCCC1

359 CN(C)CCNC(=O)OC1CCC(C=CC(=O)C2CCCCC2)CC1

360 CN1CCN(CCCCC=CC(=O)C2CCCCC2)CC1

361 CN(C)CCN(C)C1CCC(C=CC(=O)C2CCCCC2)CC1

362 CN(C)CCN(C)C1CCC(C=CC(=O)C2CCCCC2)CC1

363 O=C(C=CC1CCC(OCCCN2CCCC2=O)CC1)C1CCCCC1

364 O=C(C=CC1CCC(-C2CN(CC3CCCCC3)NN2)CC1)C1CCCCC1

365 O=C(C=CC1CCC(OC2CNCCN2)CC1)C1CCCCC1

366 CN(C)C1CCN(C(=O)C2CCC(C=CC(=O)C3CCCCC3)CC2)CC1

367 O=C(C=CC1CCC(OS(=O)(=O)CC2CCCCC2)CC1)C1CCCCC1

368 CC=CCCCOC1CCC(C=CC(=O)C2CCCCC2)CC1

369 CN(CCCOC1CCC(C=CC(=O)C2CCCCC2)CC1)C(=O)O

370 CNCCSC1CCC(C=CC(=O)C2CCCCC2)CC1

371 O=C(C=CC1CCC(SCCCN2CCCC2)CC1)C1CCCCC1

372 O=C(C=CC1CCC(OCCCN2CCCN2)CC1)C1CCCCC1

373 CC(=O)N1CCC(COC2CCC(C=CC(=O)C3CCCCC3)CC2)CC1

374 O=C(C=CC1CCC(OCCN2CCNC2)CC1)C1CCCCC1

375 CN1CCC(CCOC2CCC(C=CC(=O)C3CCCCC3)CC2)CC1

376 CN(C)CCCSC1CCC(C=CC(=O)C2CCCCC2)CC1

377 CNCCCSC1CCC(C=CC(=O)C2CCCCC2)CC1

378 CN1CCN(C2CCCC(OC3CCC(C=CC(=O)C4CCCCC4)CC3)C2)CC1

379 CN1CCN(CCCCCC=CC(=O)C2CCCCC2)CC1

380 O=C(CC1CCCS1)OC1CCC(C=CC(=O)C2CCCCC2)CC1

381 O=C(C=CC1CCC(NCCC(C2CCCCC2)C2CCCCC2)CC1)C1CCCCC1

382 O=C(C=CC1CCC(OCN2CCOCC2)CC1)C1CCCCC1

383 CC(=O)N1CCC(OC2CCC(C=CC(=O)C3CCCCC3)CC2)CC1

384 CC1CCC(CCCOC2CCC(C=CC(=O)C3CCCCC3)CC2)O1

385 O=C(C=CC1CCC(OCCCN2CCNCC2)CC1)C1CCCCC1

386 CN(CCCOC1CCC(C=CC(=O)C2CCCCC2)CC1)C1CCC1

387 CN(CCCOC1CCC(C=CC(=O)C2CCCCC2)CC1)C1CCC1

388 CN(C)CNC(=O)C1CCC(C=CC(=O)C2CCCCC2)CC1

389 CN(C)C1CCC(SC2CCC(C=CC(=O)C3CCCCC3)CC2)CC1

390 O=C(C=CC1CCC(OCC2CCNCC2)CC1)C1CCCCC1

391 CN(C)CCCCSC1CCC(C=CC(=O)C2CCCCC2)CC1

392 CN(C)CC1CCNC(-C2CCC(C=CC(=O)C3CCCCC3)CC2)C1

393 CN(C)CCCCSC1CCC(C=CC(=O)C2CCCCC2)CC1

394 CN(C)CCCCSC1CCC(C=CC(=O)C2CCCCC2)CC1

395 CC(C)(N)CCCOC1CCC(C=CC(=O)C2CCCCC2)CC1

396 CN1CCN(COC2CCC(C=CC(=O)C3CCCCC3)CC2)CC1

397 CN(C)COC1CCC(-C2CCC(C=CC(=O)C3CCCCC3)CC2)CC1

398 CN(C)CCSCC1CCC(C=CC(=O)C2CCCCC2)CC1

399 O=C(C=CC1CCC(CNC2CCC(O)CC2)CC1)C1CCCCC1

400	O=C(C=CC1CCC(OC(=O)C(C2CCCCC2)C2CCCCC2)CC1)C1CCCCC1
401	O=C(C=CC1CCC(OC2CCNCC2)CC1)C1CCCCC1
402	CN(C)CCCN(C)C1CCC(C=CC(=O)C2CCCCC2)CC1
403	CC(C)N1CCC(COC2CCC(C=CC(=O)C3CCCCC3)CC2)CC1
404	CN(C)C1CCC(C2CCC(C=CC(=O)C3CCCCC3)CC2)CC1
405	CN(C)CC1CCC(-C2CCC(C=CC(=O)C3CCCCC3)CC2)O1
406	CC(C)(C)NS(=O)(=O)C1CCC(-<br>C2CCC(C=CC(=O)C3CCCCC3)CC2)CC1
407	CN(CCCOC1CCC(C=CC(=O)C2CCCCC2)CC1)C(=O)C1CC1
408	CN(C)CCCS(=O)(=O)C1CCC(C=CC(=O)C2CCCCC2)CC1
409	CN(CCCOC1CCC(C=CC(=O)C2CCCCC2)CC1)C(=O)C1CC1
410	CN(C)CC1CCN(C2CCC(C=CC(=O)C3CCCCC3)CC2)CC1
411	CN(C)CCOCOC1CCC(C=CC(=O)C2CCCCC2)CC1
412	CC1CC(C)N(CCCOC2CCC(C=CC(=O)C3CCCCC3)CC2)N1
413	CN(CCCOC1CCC(C=CC(=O)C2CCCCC2)CC1)C(N)=O
414	CN(CCCOC1CCC(C=CC(=O)C2CCCCC2)CC1)C(N)=O
415	CC(C)N1CCC(OC2CCC(C=CC(=O)C3CCCCC3)CC2)CC1

416 O=C(C=CC1CCC(SC(C2CCCCC2)C2CCCCC2)CC1)C1CCCCC1

417 O=C(C=CC1CCC(OCCCCN2CCNC2)CC1)C1CCCCC1

418 CN(CCCOC1CCC(C=CC(=O)C2CCCCC2)CC1)C1CCCC1

419 CC(C)(C)NCCCNC1CCC(C=CC(=O)C2CCCCC2)CC1

420 CN(CCCOC1CCC(C=CC(=O)C2CCCCC2)CC1)C1CCCC1

421 O=C(C=CC1CCC(OC2CCC(O)CC2)CC1)C1CCCCC1

422 CN(C)CC=CC1CCC(C=CC(=O)C2CCCCC2)CC1

423 CN(C)CC=CC1CCC(C=CC(=O)C2CCCCC2)CC1

424 CC(C)(C)C(=O)OCOC1CCC(C=CC(=O)C2CCCCC2)CC1

425 C[SI](C)(C)CCCOC1CCC(C=CC(=O)C2CCCCC2)CC1

426 CCC(C)(C)CCCOC1CCC(C=CC(=O)C2CCCCC2)CC1

427 CCC(C)(C)CCCOC1CCC(C=CC(=O)C2CCCCC2)CC1

428 CN(C)CCCCN(C)C1CCC(C=CC(=O)C2CCCCC2)CC1

429 O=C(C=CC1CCC(C#CCCO)CC1)C1CCCCC1

430 CC1NCCN1CCCOC1CCC(C=CC(=O)C2CCCCC2)CC1

431 O=C(C=CC1CCC(NCCCN2CCCC2=O)CC1)C1CCCCC1

432 O=C(C=CC1CCC(NCCN2CCNCC2)CC1)C1CCCCC1

433 CN(O)CCCOC1CCC(C=CC(=O)C2CCCCC2)CC1

434 CC(F)(F)CCCOC1CCC(C=CC(=O)C2CCCCC2)CC1

435 CN(O)CCCOC1CCC(C=CC(=O)C2CCCCC2)CC1

436 O=C(C=CC1CCC(OCCC2CCNCC2)CC1)C1CCCCC1

437 CCN(CC)COC1CCC(C=CC(=O)C2CCCCC2)CC1

438 CC(C)N1CC(OC2CCC(C=CC(=O)C3CCCCC3)CC2)C1

439 CCN(CCCN(C)C)C1CCC(C=CC(=O)C2CCCCC2)CC1

440 CN(C)CC(=O)N(C)C1CCC(C=CC(=O)C2CCCCC2)CC1

441 O=C(C=CC1CCC(C(=O)N2CCC(N3CCCCC3)CC2)CC1)C1CCCCC1

442 CN(C)CC(=O)N(C)C1CCC(C=CC(=O)C2CCCCC2)CC1

443 CC(C)CN1CCC(OC2CCC(C=CC(=O)C3CCCCC3)CC2)CC1

444 CC=C(C)CCCOC1CCC(C=CC(=O)C2CCCCC2)CC1

445 C=CCN(C)CCOC1CCC(C=CC(=O)C2CCCCC2)CC1

446 CC1CSC(CCCOC2CCC(C=CC(=O)C3CCCCC3)CC2)N1

447 CN(N)CCCOC1CCC(C=CC(=O)C2CCCCC2)CC1

448 CN(N)CCCOC1CCC(C=CC(=O)C2CCCCC2)CC1

449 CN(C)COCC1CCC(C=CC(=O)C2CCCCC2)CC1

450 CC1CNN(CCCOC2CCC(C=CC(=O)C3CCCCC3)CC2)C1

451 CN(C)COC1CCCC(-C2CCC(C=CC(=O)C3CCCCC3)CC2)C1

452 COC(=O)C=CCOC1CCC(C=CC(=O)C2CCCCC2)CC1

453 O=C(C=CC1CCC(NCCCN2CCNCC2)CC1)C1CCCCC1

454 CC1CCN(CCCOC2CCC(C=CC(=O)C3CCCCC3)CC2)N1

455 CC(C)(CO)CCCOC1CCC(C=CC(=O)C2CCCCC2)CC1

456 CN(C)CC1CCC(OC2CCC(C=CC(=O)C3CCCCC3)CC2)CC1O

457 COC1CCC2[NH]CC(CCOC3CCC(C=CC(=O)C4CCCCC4)CC3)C2C1

458 CN(C)CCCC=CC1CCC(C=CC(=O)C2CCCCC2)CC1

459 COC(C)(C)CCCOC1CCC(C=CC(=O)C2CCCCC2)CC1

460 COC(C)(C)CCCOC1CCC(C=CC(=O)C2CCCCC2)CC1

461 O=C(C=CC1CCC(SCCCN2CCOCC2)CC1)C1CCCCC1

462 CN(C)S(=O)(=O)CCOC1CCC(C=CC(=O)C2CCCCC2)CC1

463 CC1NOC(C)C1CCCOC1CCC(C=CC(=O)C2CCCCC2)CC1

464 CN(C)CCC1CCN(C2CCC(C=CC(=O)C3CCCCC3)CC2)CC1

465 CN(C)CCC1CCN(C2CCC(C=CC(=O)C3CCCCC3)CC2)CC1

466 CN(C)CCC=CC1CCC(C=CC(=O)C2CCCCC2)CC1

467 CC1NOC(CCCOC2CCC(C=CC(=O)C3CCCCC3)CC2)N1

468 CN(C)CC1CSC(-C2CCC(C=CC(=O)C3CCCCC3)CC2)N1

469 CC#CCCCOC1CCC(C=CC(=O)C2CCCCC2)CC1

470 CC1(CCCOC2CCC(C=CC(=O)C3CCCCC3)CC2)CC1

471 CN(C)CC1NC(-C2CCC(C=CC(=O)C3CCCCC3)CC2)NO1

472 CN(C)C1CCCC(SC2CCC(C=CC(=O)C3CCCCC3)CC2)C1

473 CC(C)(CN)CCCOC1CCC(C=CC(=O)C2CCCCC2)CC1

474 CC(CCCOC1CCC(C=CC(=O)C2CCCCC2)CC1)=NO

475 CC1CC(CCCOC2CCC(C=CC(=O)C3CCCCC3)CC2)ON1

476 CN(C)COC(=O)NC1CCC(C=CC(=O)C2CCCCC2)CC1

477 CN(C)CCCC(=O)N(C)C1CCC(C=CC(=O)C2CCCCC2)CC1

478 CC1CC(OC2CCC(C=CC(=O)C3CCCCC3)CC2)NO1

479 CN(C)CCCC1CCCC(-C2CCC(C=CC(=O)C3CCCCC3)CC2)N1

480 CNOCCCOC1CCC(C=CC(=O)C2CCCCC2)CC1

481 O=C(C=CC1CCC(OCCCN2CCSCC2)CC1)C1CCCCC1

482 CN(C)CCC(=O)N(C)C1CCC(C=CC(=O)C2CCCCC2)CC1

483 CN(C)CCCC(C)(C)C1CCC(C=CC(=O)C2CCCCC2)CC1

484 O=C(C=CC1CCC(OCCN(CCO)CCO)CC1)C1CCCCC1

485 CC(=O)N(CCCOC1CCC(C=CC(=O)C2CCCCC2)CC1)C1CC1

486 C[N+](C)(C)CCCOC1CCC(C=CC(=O)C2CCCCC2)CC1

487 CN(C)C1CCC(OC2CCC(C=CC(=O)C3CCCCC3)CC2)CC1

488 C[N+](C)(C)CCCCOC1CCC(C=CC(=O)C2CCCCC2)CC1

489 CC(=O)N(O)CCCOC1CCC(C=CC(=O)C2CCCCC2)CC1

490 O=CNCCCNC1CCC(C=CC(=O)C2CCCCC2)CC1

491 CCC1NCCN1CCCOC1CCC(C=CC(=O)C2CCCCC2)CC1

492 CC1(CCCOC2CCC(C=CC(=O)C3CCCCC3)CC2)CCC1

493 CC(C)(C#N)CCCOC1CCC(C=CC(=O)C2CCCCC2)CC1

494 CN(CCCOC1CCC(C=CC(=O)C2CCCCC2)CC1)C(C)(C)C

495 CN(C)NC(=O)OC1CCC(C=CC(=O)C2CCCCC2)CC1

496 CN(C)CC1CC(-C2CCC(C=CC(=O)C3CCCCC3)CC2)NO1

497 O=C(C=CC1CCC(C#CCCCO)CC1)C1CCCCC1

498 CN(C)CCCCC(C)(C)C1CCC(C=CC(=O)C2CCCCC2)CC1

499 CN(C)CCCC1CCC(-C2CCC(C=CC(=O)C3CCCCC3)CC2)O1

500 CN(C)CCNCOC1CCC(C=CC(=O)C2CCCCC2)CC1

501 CC(=O)N(CCCN(C)C)C1CCC(C=CC(=O)C2CCCCC2)CC1

502 CCN1CCNC1CCCOC1CCC(C=CC(=O)C2CCCCC2)CC1

503 CN(C)C1CC(OC2CCC(C=CC(=O)C3CCCCC3)CC2)C1

504 CNC(C)(C)CCCOC1CCC(C=CC(=O)C2CCCCC2)CC1

505 CCN(C(=O)CCN(C)C)C1CCC(C=CC(=O)C2CCCCC2)CC1

506 CN(C)CCN(CCO)C1CCC(C=CC(=O)C2CCCCC2)CC1

507 CC1CN(CCCOC2CCC(C=CC(=O)C3CCCCC3)CC2)CN1

508 COC(=N)CCCOC1CCC(C=CC(=O)C2CCCCC2)CC1

509 CN1CCN(NC2CCC(C=CC(=O)C3CCCCC3)CC2)CC1

510 CN(C)CC1CC(-C2CCC(C=CC(=O)C3CCCCC3)CC2)ON1

511 CSC(=O)CCCOC1CCC(C=CC(=O)C2CCCCC2)CC1

512 CN(C)CCC(C)(C)C1CCC(C=CC(=O)C2CCCCC2)CC1

513 CN(CL)CCCOC1CCC(C=CC(=O)C2CCCCC2)CC1

514 CN(C)CC(=O)NCOC1CCC(C=CC(=O)C2CCCCC2)CC1

515 CN(C)CC(C)(C)COC1CCC(C=CC(=O)C2CCCCC2)CC1

516 CN(C)CC(C)(C)COC1CCC(C=CC(=O)C2CCCCC2)CC1

517 C=C(CCCN(C)C)C1CCC(C=CC(=O)C2CCCCC2)CC1

518 CC(C)=NOCCOC1CCC(C=CC(=O)C2CCCCC2)CC1

519 CN(C)NCCCC1CCC(C=CC(=O)C2CCCCC2)CC1

520 CN(CCCOC1CCC(C=CC(=O)C2CCCCC2)CC1)C(=N)N

521 CN(CCCOC1CCC(C=CC(=O)C2CCCCC2)CC1)C(=N)N

522 CN(C)CC#CC1CCC(C=CC(=O)C2CCCCC2)CC1

523 CN(C)OCCCC1CCC(C=CC(=O)C2CCCCC2)CC1

524 CN(C)OC1CCC(OC2CCC(C=CC(=O)C3CCCCC3)CC2)CC1

525 CC(=S)N(C)CCCOC1CCC(C=CC(=O)C2CCCCC2)CC1

526 CN(C)CCCC1CSC(-C2CCC(C=CC(=O)C3CCCCC3)CC2)N1

527 CN(C)CC(C)(C)OC1CCC(C=CC(=O)C2CCCCC2)CC1

528 CC1CNCN1CCCOC1CCC(C=CC(=O)C2CCCCC2)CC1

529 CN(C)CC(C)(C)OC1CCC(C=CC(=O)C2CCCCC2)CC1

530 CC(C)C1NCCN1CCCOC1CCC(C=CC(=O)C2CCCCC2)CC1

531 CC1NNNN1CCCOC1CCC(C=CC(=O)C2CCCCC2)CC1

532 CN=C(N)CCCOC1CCC(C=CC(=O)C2CCCCC2)CC1

533 CN(C)C(C)(C)COC1CCC(C=CC(=O)C2CCCCC2)CC1

534 CCN(O)CCCOC1CCC(C=CC(=O)C2CCCCC2)CC1

535 CON(C)CCCOC1CCC(C=CC(=O)C2CCCCC2)CC1

536 CC1CNC(C)N1CCCOC1CCC(C=CC(=O)C2CCCCC2)CC1

537 CSSCCCOC1CCC(C=CC(=O)C2CCCCC2)CC1

538 O=C(C=CC1CCC(ON2CCCCC2)CC1)C1CCCCC1

539 CC(C)(C)N1CC(OC2CCC(C=CC(=O)C3CCCCC3)CC2)C1

540 CON(C)CCCOC1CCC(C=CC(=O)C2CCCCC2)CC1

541 C=C(C)N(C)CCCOC1CCC(C=CC(=O)C2CCCCC2)CC1

542 CN1CNC(CCOC2CCC(C=CC(=O)C3CCCCC3)CC2)C1

543 CN(C=O)CCCOC1CCC(C=CC(=O)C2CCCCC2)CC1

544 CN(C=O)CCCOC1CCC(C=CC(=O)C2CCCCC2)CC1

545 CN(CCCOC1CCC(C=CC(=O)C2CCCCC2)CC1)C1CNC1

546 CN(C)CCCC1CNN(-C2CCC(C=CC(=O)C3CCCCC3)CC2)C1

547 CN1CNCC1CCOC1CCC(C=CC(=O)C2CCCCC2)CC1

548 CN(C)CCCC#CC1CCC(C=CC(=O)C2CCCCC2)CC1

549 CN(C)CC=CCOC1CCC(C=CC(=O)C2CCCCC2)CC1

550 CN(C)CC=CCOC1CCC(C=CC(=O)C2CCCCC2)CC1

551 CC(C)=NCCOC1CCC(C=CC(=O)C2CCCCC2)CC1

552 CN(C)C(C)(C)CCCOC1CCC(C=CC(=O)C2CCCCC2)CC1

553 CN(C)C(C)(C)CCCOC1CCC(C=CC(=O)C2CCCCC2)CC1

554 CN(C)CC1COC(-C2CCC(C=CC(=O)C3CCCCC3)CC2)N1

555 CN(C)C=CC(=O)OC1CCC(C=CC(=O)C2CCCCC2)CC1

556 CN(C)C=CC(=O)OC1CCC(C=CC(=O)C2CCCCC2)CC1

557 O=C(C=CC1CCC(NCCCC2NCC[NH]2)CC1)C1CCCCC1

558 CC(=N)N(C)CCCOC1CCC(C=CC(=O)C2CCCCC2)CC1

559 CN=C(C)CCCOC1CCC(C=CC(=O)C2CCCCC2)CC1

560 CN(C)CCCN(C1CCC(C=CC(=O)C2CCCCC2)CC1)S(C)(=O)=O

561 CN(C)CC1CNC(-C2CCC(C=CC(=O)C3CCCCC3)CC2)CN1

562 CC(C)N(CCCN(C)C)C1CCC(C=CC(=O)C2CCCCC2)CC1

563 CN(C)CCCC1NOC(-C2CCC(C=CC(=O)C3CCCCC3)CC2)N1

564 CN(F)CCCOC1CCC(C=CC(=O)C2CCCCC2)CC1

565 CN(C)CCC#CC1CCC(C=CC(=O)C2CCCCC2)CC1

566 CN(C)CCCN(C1CCC(C=CC(=O)C2CCCCC2)CC1)C1CC1

567 CN(C)CNC(=O)OC1CCC(C=CC(=O)C2CCCCC2)CC1

568 CN(C)COCOC1CCC(C=CC(=O)C2CCCCC2)CC1

569 CN(CCCOC1CCC(C=CC(=O)C2CCCCC2)CC1)N=O

570 CC(=CCCCN(C)C)C1CCC(C=CC(=O)C2CCCCC2)CC1

571 CN(C)CCCSC(=O)C1CCC(C=CC(=O)C2CCCCC2)CC1

572 CC(C)=COCCOC1CCC(C=CC(=O)C2CCCCC2)CC1

573 CN(C)CC=CCC1CCC(C=CC(=O)C2CCCCC2)CC1

574 CN(C)CCCC(=NO)C1CCC(C=CC(=O)C2CCCCC2)CC1

575 CN(C)CCC(C)(C)OC1CCC(C=CC(=O)C2CCCCC2)CC1

576 CO[SI](C)(C)CCCOC1CCC(C=CC(=O)C2CCCCC2)CC1

577 CN(C)CCCNC(C)(C)C1CCC(C=CC(=O)C2CCCCC2)CC1

578 CN(C)CCCC1CC(-C2CCC(C=CC(=O)C3CCCCC3)CC2)ON1

579 CN(BR)CCCOC1CCC(C=CC(=O)C2CCCCC2)CC1

580 CN(C)CCCOOC1CCC(C=CC(=O)C2CCCCC2)CC1

581 CN(C)OC1CCCC(OC2CCC(C=CC(=O)C3CCCCC3)CC2)C1

582 CN(C)CCCOOC1CCC(C=CC(=O)C2CCCCC2)CC1

583 CN(C)CCOOC1CCC(C=CC(=O)C2CCCCC2)CC1

584 CN(C)CCOOC1CCC(C=CC(=O)C2CCCCC2)CC1

585 CC=NCCCOC1CCC(C=CC(=O)C2CCCCC2)CC1

586 CN(C)OCCNC1CCC(C=CC(=O)C2CCCCC2)CC1

587 O=C(C=CC1CCC(OCCCN2CC=CCC2)CC1)C1CCCCC1

588 CN(C)CCCC1CCN(-C2CCC(C=CC(=O)C3CCCCC3)CC2)N1

589 CC(C)CCNOC1CCC(C=CC(=O)C2CCCCC2)CC1

590 CN(S)CCCOC1CCC(C=CC(=O)C2CCCCC2)CC1

591 CC1(CCCOC2CCC(C=CC(=O)C3CCCCC3)CC2)COC1

592 CN(C)CCCC(F)(F)C1CCC(C=CC(=O)C2CCCCC2)CC1

593 CN(C)CCCC1NCCN1-C1CCC(C=CC(=O)C2CCCCC2)CC1

594 CN(C)CCCC1(C2CCC(C=CC(=O)C3CCCCC3)CC2)CC1

595 CNN(C)CCCOC1CCC(C=CC(=O)C2CCCCC2)CC1

596 CNN(C)CCCOC1CCC(C=CC(=O)C2CCCCC2)CC1

597 CN(C)CCCOC(=N)C1CCC(C=CC(=O)C2CCCCC2)CC1

598 CC1NOC(-C2CCC(C=CC(=O)C3CCCCC3)CC2)C1CN(C)C

599 CC[N+](C)(C)CCCOC1CCC(C=CC(=O)C2CCCCC2)CC1

600 CC[N+](C)(C)CCCOC1CCC(C=CC(=O)C2CCCCC2)CC1

601 CN(C)CCCC1COC(-C2CCC(C=CC(=O)C3CCCCC3)CC2)N1

602 CNCCCOOC1CCC(C=CC(=O)C2CCCCC2)CC1

603 CN(C)CCC1NCCN1-C1CCC(C=CC(=O)C2CCCCC2)CC1

604 CN(C)CCSCOC1CCC(C=CC(=O)C2CCCCC2)CC1

605 CN(C)CCCC1NNNN1-C1CCC(C=CC(=O)C2CCCCC2)CC1

606 CN(C)C=CCCOC1CCC(C=CC(=O)C2CCCCC2)CC1

607 CN1CCN(OC2CCC(C=CC(=O)C3CCCCC3)CC2)CC1

608 CN(C)OCOC1CCC(C=CC(=O)C2CCCCC2)CC1

609 C=CN(C)CCCOC1CCC(C=CC(=O)C2CCCCC2)CC1

610 CN(C)CC#CCOC1CCC(C=CC(=O)C2CCCCC2)CC1

611 CN(C)CC#CCOC1CCC(C=CC(=O)C2CCCCC2)CC1

612 CN(CCCOC1CCC(C=CC(=O)C2CCCCC2)CC1)N1CC1

613 CN(I)CCCOC1CCC(C=CC(=O)C2CCCCC2)CC1

614 CN(CCCOC1CCC(C=CC(=O)C2CCCCC2)CC1)C(F)F

615 CC1CCN(OC2CCC(C=CC(=O)C3CCCCC3)CC2)CC1

616 CP(C)(=O)CCCOC1CCC(C=CC(=O)C2CCCCC2)CC1

617 CN(C)C1CC(-C2CCC(C=CC(=O)C3CCCCC3)CC2)ON1

618 O=C(C=CC1CCC(OCCC2C[NH]CN2)CC1)C1CCCCC1

619 CN(C)N(C)CCOC1CCC(C=CC(=O)C2CCCCC2)CC1

620 CC(=CN(C)C)COC1CCC(C=CC(=O)C2CCCCC2)CC1

621 O=C(C=CC1CCC(SC2CCNCC2)CC1)C1CCCCC1

622 CN(C)CCCNOC1CCC(C=CC(=O)C2CCCCC2)CC1

623 CN(C)CCCNOC1CCC(C=CC(=O)C2CCCCC2)CC1

624	CN(C)CCCNOC1CCC(C=CC(=O)C2CCCCC2)CC1
625	CC1NNN(CCCOC2CCC(C=CC(=O)C3CCCCC3)CC2)N1
626	CN(C)CCNOC1CCC(C=CC(=O)C2CCCCC2)CC1
627	CN(C)CCNOC1CCC(C=CC(=O)C2CCCCC2)CC1
628	CN(CCCOC1CCC(C=CC(=O)C2CCCCC2)CC1)[N+](=O)[O-]
629	CN(CCCOC1CCC(C=CC(=O)C2CCCCC2)CC1)C1CCCS1
630	CC1CN(C)C(CCCOC2CCC(C=CC(=O)C3CCCCC3)CC2)N1
631	C=C(CCN(C)C)OC1CCC(C=CC(=O)C2CCCCC2)CC1
632	CN(C)CCCC(=O)C(C)(C)C1CCC(C=CC(=O)C2CCCCC2)CC1
633	C=C(CN(C)C)OC1CCC(C=CC(=O)C2CCCCC2)CC1
634	CN(C)CC(=O)NOC1CCC(C=CC(=O)C2CCCCC2)CC1
635	CN(C)CCOC(C)(C)C1CCC(C=CC(=O)C2CCCCC2)CC1
636	CN(C)CC(=O)NOC1CCC(C=CC(=O)C2CCCCC2)CC1
637	CN(C)CCC(=O)NOC1CCC(C=CC(=O)C2CCCCC2)CC1
638	CN(C)N(C)CCCOC1CCC(C=CC(=O)C2CCCCC2)CC1
639	CN(C)N(C)CCCOC1CCC(C=CC(=O)C2CCCCC2)CC1

640 CN(CCCOC1CCC(C=CC(=O)C2CCCCC2)CC1)C1CCCO1

641 CSC(C)(C)CCCOC1CCC(C=CC(=O)C2CCCCC2)CC1

642 CN(C)CCCSC1NNNN1-C1CCC(C=CC(=O)C2CCCCC2)CC1

643 CN(CCCOC1CCC(C=CC(=O)C2CCCCC2)CC1)N1CCC1

644 CN(C)CCCOC(C)(C)C1CCC(C=CC(=O)C2CCCCC2)CC1

645 C[N+](C)([O-])CCCOC1CCC(C=CC(=O)C2CCCCC2)CC1

646 CN(CCCOC1CCC(C=CC(=O)C2CCCCC2)CC1)C(F)(F)F

647 CC1CCC(CCCN(C)C)N1-C1CCC(C=CC(=O)C2CCCCC2)CC1

648 CN(C)C=CC=CC1CCC(C=CC(=O)C2CCCCC2)CC1

649 CN(CCCOC1CCC(C=CC(=O)C2CCCCC2)CC1)C(C)(C)O

650 CN(C#N)CCCOC1CCC(C=CC(=O)C2CCCCC2)CC1

651 CN(C#N)CCCOC1CCC(C=CC(=O)C2CCCCC2)CC1

652 CN(C)CC1(COC2CCC(C=CC(=O)C3CCCCC3)CC2)CC1

653 O=C(C=CC1CCC(NCCC2C[NH]CN2)CC1)C1CCCCC1

654 CC1NC(CCCN(C)C)SC1-C1CCC(C=CC(=O)C2CCCCC2)CC1

655 CC1=NCCN1CCCOC1CCC(C=CC(=O)C2CCCCC2)CC1

656 COCC=COC1CCC(C=CC(=O)C2CCCCC2)CC1

657 CC(C)(CCCOC1CCC(C=CC(=O)C2CCCCC2)CC1)[N+](=O)[O-]

658 O=C([O-])CCCNC1CCC(C=CC(=O)C2CCCCC2)CC1

659 C[N+](C)(O)CCCOC1CCC(C=CC(=O)C2CCCCC2)CC1

660 CC1NC(C)C(CCCOC2CCC(C=CC(=O)C3CCCCC3)CC2)O1

661 C[S+](C)CCCOC1CCC(C=CC(=O)C2CCCCC2)CC1

662 CN(C)C(=O)COOC1CCC(C=CC(=O)C2CCCCC2)CC1

663 CN(CCCOC1CCC(C=CC(=O)C2CCCCC2)CC1)NN

664 CN(CCCOC1CCC(C=CC(=O)C2CCCCC2)CC1)NO

665 CC(=CN(C)C)OC1CCC(C=CC(=O)C2CCCCC2)CC1

666 CC[N+](C)(CC)CCOC1CCC(C=CC(=O)C2CCCCC2)CC1

667 CNC(=N)CCCOC1CCC(C=CC(=O)C2CCCCC2)CC1

668 CN(CCCOC1CCC(C=CC(=O)C2CCCCC2)CC1)C(CL)CL

669 CN(C)CCCC1CN(-C2CCC(C=CC(=O)C3CCCCC3)CC2)CN1

670 CN(CCCOC1CCC(C=CC(=O)C2CCCCC2)CC1)C(=O)[O-]

671 CC(O)CCOC1CCC(C=CC(=O)C2CCCCC2)CC1

672 CNC(=O)C=COC1CCC(C=CC(=O)C2CCCCC2)CC1

673 CCN(C)CCOOC1CCC(C=CC(=O)C2CCCCC2)CC1

674 O=C(C=CC1CCC(OCCC2NNN[NH]2)CC1)C1CCCCC1

675 CN(C)C=NCCOC1CCC(C=CC(=O)C2CCCCC2)CC1

676 CN(C)SCCCC1CCC(C=CC(=O)C2CCCCC2)CC1

677 C#CN(C)CCCOC1CCC(C=CC(=O)C2CCCCC2)CC1

678 CN(CCCOC1CCC(C=CC(=O)C2CCCCC2)CC1)C(C)(F)F

679 O=C(C=CC1CCC(OCC(O)CO)CC1)C1CCCCC1

680 CN(C)SCCC1CCC(C=CC(=O)C2CCCCC2)CC1

681 CC[N+](C)(CC)CCCOC1CCC(C=CC(=O)C2CCCCC2)CC1

682 C[SE]CCCOC1CCC(C=CC(=O)C2CCCCC2)CC1

683 CN(C)CC(F)(F)COC1CCC(C=CC(=O)C2CCCCC2)CC1

684 CN1CCN=C1COC1CCC(C=CC(=O)C2CCCCC2)CC1

685 CNC1NCCN1CCCOC1CCC(C=CC(=O)C2CCCCC2)CC1

686 CN(CCCOC1CCC(C=CC(=O)C2CCCCC2)CC1)C(C)(C)N

687 CC1ONC(CCCN(C)C)C1-C1CCC(C=CC(=O)C2CCCCC2)CC1

688 CN(C)CCCNC(=N)C1CCC(C=CC(=O)C2CCCCC2)CC1

689 CN(C)CCC=COC1CCC(C=CC(=O)C2CCCCC2)CC1

690 CN(C)C=CCNC1CCC(C=CC(=O)C2CCCCC2)CC1

691 CC(O)CCCOC1CCC(C=CC(=O)C2CCCCC2)CC1

692 CN(C)CC=COC1CCC(C=CC(=O)C2CCCCC2)CC1

693 CN(CCCOC1CCC(C=CC(=O)C2CCCCC2)CC1)N1CCCC1

694 CN(CCCOC1CCC(C=CC(=O)C2CCCCC2)CC1)C(BR)BR

695 CN1CC=C(OC2CCC(C=CC(=O)C3CCCCC3)CC2)CC1

696 CN(C)CCCSSC1CCC(C=CC(=O)C2CCCCC2)CC1

697 O=C(C=CC1CCC(C2CCN(CCN3CCNC3=O)CC2)CC1)C1CCCCC1

698 CN(CCCOC1CCC(C=CC(=O)C2CCCCC2)CC1)[SI](C)(C)C

699 O=C(C=CC1CCC(OCC2CCCO2)CC1)C1CCCCC1

700 CN(C)CCCSOC1CCC(C=CC(=O)C2CCCCC2)CC1

701 CSN(C)CCCOC1CCC(C=CC(=O)C2CCCCC2)CC1

702 CN(C)CCCSOC1CCC(C=CC(=O)C2CCCCC2)CC1

703 CSN(C)CCCOC1CCC(C=CC(=O)C2CCCCC2)CC1

704 CC(N)CCCOC1CCC(C=CC(=O)C2CCCCC2)CC1

705 CN(C)CCCN(C=O)C1CCC(C=CC(=O)C2CCCCC2)CC1

706 CC(=CCN(C)C)OC1CCC(C=CC(=O)C2CCCCC2)CC1

707 CCC(C)CCCOC1CCC(C=CC(=O)C2CCCCC2)CC1

708 CCC(C)CCCOC1CCC(C=CC(=O)C2CCCCC2)CC1

709 CN(CCCOC1CCC(C=CC(=O)C2CCCCC2)CC1)NC1CC1

710 CN(C)CCCC1CNCN1-C1CCC(C=CC(=O)C2CCCCC2)CC1

711 CN(C)CCCC=NC1CCC(C=CC(=O)C2CCCCC2)CC1

712 CNCCCSOC1CCC(C=CC(=O)C2CCCCC2)CC1

713 CN(CCCOC1CCC(C=CC(=O)C2CCCCC2)CC1)C1CCON1

714 CC(C)[N+](C)(C)CCCOC1CCC(C=CC(=O)C2CCCCC2)CC1

715 CN1CCC(OOC2CCC(C=CC(=O)C3CCCCC3)CC2)CC1

716 CN(CCCOC1CCC(C=CC(=O)C2CCCCC2)CC1)C(F)(F)CL

717 NCC(C1CCCCC1)C1CCC(C=CC(=O)C2CCCCC2)CC1

718 CN(C)C(=O)C=COC1CCC(C=CC(=O)C2CCCCC2)CC1

719 COCC(O)COC1CCC(C=CC(=O)C2CCCCC2)CC1

720 CN(C)CCCC1NCN(-C2CCC(C=CC(=O)C3CCCCC3)CC2)N1

721 CC(C)CC(C)OC1CCC(C=CC(=O)C2CCCCC2)CC1

722 CN(CCCOC1CCC(C=CC(=O)C2CCCCC2)CC1)C1NNCS1

723 CN(C)CCSOC1CCC(C=CC(=O)C2CCCCC2)CC1

724 CN(C)CCSOC1CCC(C=CC(=O)C2CCCCC2)CC1

725 CN(CCCOC1CCC(C=CC(=O)C2CCCCC2)CC1)C1CCC[NH]1

726 CNCC=COC1CCC(C=CC(=O)C2CCCCC2)CC1

727 CN(CCCOC1CCC(C=CC(=O)C2CCCCC2)CC1)C1=CCCC1

728 CC1CN1CCCOC1CCC(C=CC(=O)C2CCCCC2)CC1

729 CN(CCCOC1CCC(C=CC(=O)C2CCCCC2)CC1)C1CSCN1

730 NCC(O)COC1CCC(C=CC(=O)C2CCCCC2)CC1

731 CN(CCCOC1CCC(C=CC(=O)C2CCCCC2)CC1)C1CN[NH]C1

732 CN(CCCOC1CCC(C=CC(=O)C2CCCCC2)CC1)C1CC[NH]C1

733 CC1CCSC1N(C)CCCOC1CCC(C=CC(=O)C2CCCCC2)CC1

734 CN1CCC=C(OC2CCC(C=CC(=O)C3CCCCC3)CC2)C1

735 NCC(CC1CCCCC1)C1CCC(C=CC(=O)C2CCCCC2)CC1

736 CN(C)CCC[SI](C)(C)C1CCC(C=CC(=O)C2CCCCC2)CC1

737 CN(CCCOC1CCC(C=CC(=O)C2CCCCC2)CC1)C1CCCN1C

738 CNC(C)CCOC1CCC(C=CC(=O)C2CCCCC2)CC1

739 C[N+]1(CCOC2CCC(C=CC(=O)C3CCCCC3)CC2)CCCCC1

740 CN(C)CCCC1NNN(-C2CCC(C=CC(=O)C3CCCCC3)CC2)N1

741 CN1CCNC1C(C)(C)CCCOC1CCC(C=CC(=O)C2CCCCC2)CC1

742 C[N+]1(CCCOC2CCC(C=CC(=O)C3CCCCC3)CC2)CCCC1

743 CC1CCN(CCCOC2CCC(C=CC(=O)C3CCCCC3)CC2)C1

744 CN(CCCOC1CCC(C=CC(=O)C2CCCCC2)CC1)C1CCNN1C

745 CN(C)CC1(OC2CCC(C=CC(=O)C3CCCCC3)CC2)CC1

746 CC(CCCOC1CCC(C=CC(=O)C2CCCCC2)CC1)C(=O)O

747 CN(C)CNOC1CCC(C=CC(=O)C2CCCCC2)CC1

748 CN(CCCOC1CCC(C=CC(=O)C2CCCCC2)CC1)OC1CC1

749 CN(C)C#CCOC1CCC(C=CC(=O)C2CCCCC2)CC1

750 C[P+](C)(C)CCCOC1CCC(C=CC(=O)C2CCCCC2)CC1

751 CC1(C)CC(OC2CCC(C=CC(=O)C3CCCCC3)CC2)CC(C)(C)N1

752 CN(C)CCN(C)OC1CCC(C=CC(=O)C2CCCCC2)CC1

753 C[N+]1(CCCOC2CCC(C=CC(=O)C3CCCCC3)CC2)CCCCC1

754 CN(C)CCN(C)OC1CCC(C=CC(=O)C2CCCCC2)CC1

755 CN(C)CC(=O)N(C)OC1CCC(C=CC(=O)C2CCCCC2)CC1

756 CNC(C)CCCOC1CCC(C=CC(=O)C2CCCCC2)CC1

757 CN(C)C#CCCOC1CCC(C=CC(=O)C2CCCCC2)CC1

758 CN(C)CCCN(C)OC1CCC(C=CC(=O)C2CCCCC2)CC1

759 COC(C)CCOC1CCC(C=CC(=O)C2CCCCC2)CC1

760 CC(O)CNCCOC1CCC(C=CC(=O)C2CCCCC2)CC1

761 CCN(C)CC=COC1CCC(C=CC(=O)C2CCCCC2)CC1

762 O=C(C=CC1CCC(OCC(O)CN2CCCC2)CC1)C1CCCCC1

763 CN(C)CCCN=NC1CCC(C=CC(=O)C2CCCCC2)CC1

764 CNCC(O)COC1CCC(C=CC(=O)C2CCCCC2)CC1

765 CN(CCCOC1CCC(C=CC(=O)C2CCCCC2)CC1)C(CL)(CL)CL

766 CN(C)CSCOC1CCC(C=CC(=O)C2CCCCC2)CC1

767 CN1CCC(COC2CCC(C=CC(=O)C3CCCCC3)CC2)C1

768 CNC1=NCCN1CCCOC1CCC(C=CC(=O)C2CCCCC2)CC1

769 CC(CCC1CCC(C=CC(=O)C2CCCCC2)CC1)N(C)C

770 CC(COC1CCC(C=CC(=O)C2CCCCC2)CC1)N(C)C

771 CC(C)C(C)CCCOC1CCC(C=CC(=O)C2CCCCC2)CC1

772 CC1CCCN(CCCOC2CCC(C=CC(=O)C3CCCCC3)CC2)C1

773 CN(C)CCCSC(C)(C)C1CCC(C=CC(=O)C2CCCCC2)CC1

774 CN(CCCOC1CCC(C=CC(=O)C2CCCCC2)CC1)C1NCCO1

775 CN(CCCOC1CCC(C=CC(=O)C2CCCCC2)CC1)C1NN[NH]N1

776 CN=C(S)CCCOC1CCC(C=CC(=O)C2CCCCC2)CC1

777 COC(C)CCCOC1CCC(C=CC(=O)C2CCCCC2)CC1

778 CN1CCCC(OC2CCC(C=CC(=O)C3CCCCC3)CC2)CC1

779 CC1CCCN1CCCOC1CCC(C=CC(=O)C2CCCCC2)CC1

780 O=C(C=CC1CCC(OCC2CCOC2)CC1)C1CCCCC1

781 CN(C)CCC(O)C1CCC(C=CC(=O)C2CCCCC2)CC1

782 CC(=COC1CCC(C=CC(=O)C2CCCCC2)CC1)CN(C)C

783 CN(C)CC#COC1CCC(C=CC(=O)C2CCCCC2)CC1

784 CN(C)CCC(C1CCCCC1)C1CCC(C=CC(=O)C2CCCCC2)CC1

785 CCC(N)CCCOC1CCC(C=CC(=O)C2CCCCC2)CC1

786 CN1CCCC1COC1CCC(C=CC(=O)C2CCCCC2)CC1

787 CC(NCCCN(C)C)C1CCC(C=CC(=O)C2CCCCC2)CC1

788 CC(CN(C)C)OC1CCC(C=CC(=O)C2CCCCC2)CC1

789 CN1CCCC(COC2CCC(C=CC(=O)C3CCCCC3)CC2)C1

790 CC(CN(C)C)OC1CCC(C=CC(=O)C2CCCCC2)CC1

791 C[N+]1(C)CCC(OC2CCC(C=CC(=O)C3CCCCC3)CC2)CC1

792 CN1CCC(OC2CCC(C=CC(=O)C3CCCCC3)CC2)C1

793 CC(C)NCC(O)COC1CCC(C=CC(=O)C2CCCCC2)CC1

794 CC1CC(=O)N(C)N1CCCOC1CCC(C=CC(=O)C2CCCCC2)CC1

795 C[N+](C)(C)CCOOC1CCC(C=CC(=O)C2CCCCC2)CC1

796 O=C(C=CC1CCC(OCCCN2CCC(O)C2)CC1)C1CCCCC1

797 CN(CCCOC1CCC(C=CC(=O)C2CCCCC2)CC1)C1CCN[NH]1

798 CN(C)C=CCN(C)C1CCC(C=CC(=O)C2CCCCC2)CC1

799 CC1CCCCN1CCCOC1CCC(C=CC(=O)C2CCCCC2)CC1

800 CC(C)N(C)CC=COC1CCC(C=CC(=O)C2CCCCC2)CC1

801 CC(C)N(C)CC=COC1CCC(C=CC(=O)C2CCCCC2)CC1

802 CN(C)CC=NOC1CCC(C=CC(=O)C2CCCCC2)CC1

803 CN1CCCCC1COC1CCC(C=CC(=O)C2CCCCC2)CC1

804 CN(CCCOC1CCC(C=CC(=O)C2CCCCC2)CC1)C1CCNO1

805 O=C(C=CC1CCC(OCC2CCNC2)CC1)C1CCCCC1

806 CCN1CCCC(OC2CCC(C=CC(=O)C3CCCCC3)CC2)C1

807 CN1CCCC1CCOC1CCC(C=CC(=O)C2CCCCC2)CC1

808 CC(CCCN(C)C)OC1CCC(C=CC(=O)C2CCCCC2)CC1

809 CN1CCCCC1CCOC1CCC(C=CC(=O)C2CCCCC2)CC1

810 CCN(CC)CC(O)COC1CCC(C=CC(=O)C2CCCCC2)CC1

811 CN(C)CCC(O)COC1CCC(C=CC(=O)C2CCCCC2)CC1

812 CN1CC[N+](CCCOC2CCC(C=CC(=O)C3CCCCC3)CC2)C1

813 CC1CN(CCCOC2CCC(C=CC(=O)C3CCCCC3)CC2)CCO1

814 CC(OCCN(C)C)C1CCC(C=CC(=O)C2CCCCC2)CC1

815 CN(CCCOC1CCC(C=CC(=O)C2CCCCC2)CC1)C1CC[NH]N1

816 CC(OCCCN(C)C)C1CCC(C=CC(=O)C2CCCCC2)CC1

817 CC1C[N+](C)CN1CCCOC1CCC(C=CC(=O)C2CCCCC2)CC1

818 O=C(C=CC1CCC(OCCC2CCCCN2)CC1)C1CCCCC1

819 CN(C)CC(OC1CCC(C=CC(=O)C2CCCCC2)CC1)C1CCCCC1

820 O=C(C=CC1CCC(OCCCN2CCC(F)C2)CC1)C1CCCCC1

821 CCC(CN(C)C)OC1CCC(C=CC(=O)C2CCCCC2)CC1

822 CN(C)C=CC=COC1CCC(C=CC(=O)C2CCCCC2)CC1

823 CC1COCCN1CCCOC1CCC(C=CC(=O)C2CCCCC2)CC1

824 CN(CCCOC1CCC(C=CC(=O)C2CCCCC2)CC1)C1CC2CCC1C2

825 O=C(C=CC1CCC(OC2CC3CCC(C2)N3)CC1)C1CCCCC1

826 CCN(CCCOC1CCC(C=CC(C2CCCCC2)=O)CC1)C

In vitro inhibition of AChE

The inhibitory potency of target compounds on AChE was determined using spectroscopic method of Ellman *et al.*, (1961) [43] with slight modification and was expressed as IC_{50}, rat cortex homogenate was used as the resource of AChE. For assay of AChE activity, a reaction mixture containing 100µl acetylthiocholine iodide 0.075M/L, 100µl sodium phosphate buffer (0.1M/L, pH 7.4), 20µl homogenate or serum and different concentrations of test compounds 20µl was incubated at 37°C for 15 min. The reaction was terminated by adding 50µl 3% sodium lauryl sulfate, then, 50µl, 0.2% of 5,5'–dithio-bis-(2-nitrobenzoic acid) (DTNB, 10 mg DTNB in 100 mL of Sorenson phosphate buffer, pH 8.0) was added to produce the yellow anion of 5-thio-2-nitro-benzoic acid. Assays were carried out with a blank containing all components except AChE in order to account for non-enzymatic reaction. The values of IC_{50} were calculated by UV spectroscopy from the absorbance changes at 450 nm. A positive control of donepezil was used in the same range of concentrations. Each concentration was analyzed in triplicate. Data from concentration–inhibition experiments of the inhibitors were calculated by nonlinear regression analysis, using the GraphPad Prism 5 program.

In vitro advanced glycation end-product (AGEs) formation inhibitory activity

The assay for the ability of the synthesized compounds to inhibit the glucose-mediated protein glycation and the development of fluorescent AGEs was performed. Different concentrations of various compounds were prepared by dissolving in DMSO. Anti-glycation assay was performed according to the methods reported by Matsuura and colleagues with slight modification. In all experiments, about 500 µl of Bovine serum albumin (1mg/ml final concentration) was incubated with 400 µl of glucose (500 mM) in the presence of 100 µl of test compounds, aminoguanidine or PBS as control buffer at different concentrations, The reaction was allowed to proceed at 60°C for 24hrs and thereafter reaction was stopped by adding 10 µl of 100% (w/v) trichloroacetic acid (TCA). Then the mixture was kept at 4°C for 10 min before subjected to centrifugation at 15,000 rpm. The precipitate was redissolved in 1ml alkaline PBS (pH10) and immediately quantified for relative amount of glycated BSA based on fluorescence intensity by spectrofluorometer LS-55 (Perkin Elmer) at 370 nm (excitation) and 440 nm (emission). Aminoguanidine was used as a positive control. Percentage inhibition was calculated. All experiments were performed in triplicate.

Radical scavenging activity (DPPH Method)

The stable 1, 1-diphenyl-2-picryl hydrazyl radical (DPPH) was used for determination of free radical-scavenging activity of the test compounds. The 0.1 mM solution of DPPH in methanol (39.4 mg in 1000 ml) was freshly prepared. Different concentrations of test compounds were added with an equal volume to methanol solution of DPPH. After 30 min at room temperature, the absorbance was recorded at 517 nm. Ascorbic acid was used as standard. IC_{50} values denote the concentration of sample, which is required to scavenge 50% of DPPH free radicals. IC_{50} value was determined from the plotted graph of scavenging activity against the different concentrations of test compounds. Ascorbic acid was applied as positive drug (Blois, 1958) [75].
